# Shrimp allergen extract immunotherapy induces prolonged immune tolerance in a gastro-food allergy mouse model

**DOI:** 10.1371/journal.pone.0315312

**Published:** 2024-12-27

**Authors:** Honey Dzikri Marhaeny, Lutfiatur Rohmah, Yusuf Alif Pratama, Salsabilla Madudari Kasatu, Andang Miatmoko, Rafi Addimaysqi, Geert van den Bogaart, Franz Y. Ho, Muhammad Taher, Junaidi Khotib

**Affiliations:** 1 Department of Pharmacy Practice, Faculty of Pharmacy, Airlangga University, Surabaya, Indonesia; 2 Department of Pharmaceutical Science, Faculty of Pharmacy, Airlangga University, Surabaya, Indonesia; 3 Faculty of Medicine, Airlangga University, Surabaya, Indonesia; 4 Department of Molecular Immunology and Microbiology, Groningen Biomolecular Sciences and Biotechnology Institute, Faculty of Science Engineering, University of Groningen, Groningen, The Netherlands; 5 GBB Proteomics, Groningen Biomolecular Sciences and Biotechnology Institute, Faculty of Science Engineering, University of Groningen, Groningen, The Netherlands; 6 Department of Pharmaceutical Technology, Kulliyyah of Pharmacy, International Islamic University Malaysia, Kuantan, Pahang, Malaysia; University of Maryland School of Medicine, UNITED STATES OF AMERICA

## Abstract

Food allergies are a global health problem that continues to grow annually, with a prevalence of more than 10%. Shrimp allergy is the most common and life-threatening allergy. There is no cure for food allergies, but shrimp allergen extract (SAE) offers promise as a treatment through allergen-specific immunotherapy (AIT). However, whether SAE induces immunological tolerance in seafood allergies remains to be established. This study aimed to determine the effectiveness of SAE in inducing immunological tolerance in a gastro-food allergy mouse model. For the immunotherapy evaluation, mice (n = 24) were intraperitoneally (i.p.) sensitized with 1 mg alum and 100 μg SAE in PBS on days 0, 7, and 14 and randomly divided into four groups of six: a negative control (NC) and high- to low-dose immunotherapy (HI, MI, and LI). The untreated group (n = 6) only received 1 mg alum in PBS (i.p.). All groups were challenged with 400 μg SAE (i.g.) on days 21, 22, 23, 53, and 58. Following the challenge, SAE-sensitized mice from the immunotherapy group were treated (i.p.) with 10 μg SAE for LI, 50 μg SAE for MI, and 100 μg SAE for HI on days 32, 39, and 46. The untreated and NC groups only received PBS (i.p.). All mice were euthanized on day 59. As the results, we found that SAE immunotherapy reduced systemic allergy symptom scores, serum IL-4 levels, IL-4 and *FcεR1α* mRNA relative expression, and mast cell degranulation in ileum tissue in allergic mice while increasing *Foxp3* and IL-10 mRNA relative expression. Notably, we observed an increased ratio of IL-10 to IL-4 mRNA expression, demonstrating the efficacy of SAE immunotherapy in promoting desensitization. Thus, SAE can be developed as an immunotherapeutic agent for food allergies by inducing prolonged allergy tolerance with a wide range of allergen targets.

## Introduction

Food allergies have surged in recent decades, affecting over 10% of the global population, driven by food accessibility and dietary habits [1]. Seafood, particularly shrimp, allergies are the most common causes of anaphylaxis and can be life-threatening for food allergy sufferers, with 7.7% and 2.3% prevalence in Asia and the USA, respectively [2, 3]. Tropomyosin (TPM) is a major cross-reactive allergen reported in 23–83% of allergy sufferers. Moreover, allergen-specific IgE against whole shrimp extract was detected in 94% of allergic sufferers, implying that other shellfish allergens may be implicated [4]. For individuals sensitive to food allergens, even small amounts of allergenic foods can trigger severe reactions. Consequently, nearly 50% of patients with shrimp allergies require at least one emergency treatment due to accidental allergen exposure [5–8].

In allergic reactions, ingesting food allergens triggers alarmin cytokines, initiating a Th2 response by producing type-2 cytokines, which drive allergen-specific IgE production by B cells to induce sensitization. Subsequently, re-exposure to allergens can trigger mast cell degranulation and release inflammatory mediators like cytokines, histamine, and prostaglandins, leading to hypersensitivity reactions [5–7]. Unfortunately, no cure for food allergies is currently available. The only management strategy that can be used is to avoid food allergens and treat symptoms with adrenaline and antihistamines [5, 8]. Recently, allergen immunotherapy (AIT) has emerged as a promising therapeutic strategy for treating food allergies through allergic desensitization. The desensitization phase aims to limit allergic reactions by inducing allergen tolerance through a reduction in the Th2/Th1 ratio, concomitant with a reduction in Th2-cytokines, such as IL-4. Immune tolerance is accompanied by an increase in the number of T regulatory (Treg) cells, which play an essential role in maintaining immune homeostasis and are regulated by the transcription factor forkhead box P3 (*Foxp3*). Treg cell activation results in the production of IL-10 and TGF-β, which stimulate IgG and IgA production and suppress IgE [5, 7, 9].

An increasing number of studies show that AIT could be an effective therapy for allergies. For example, oral immunotherapy (OIT) with ovalbumin suppressed allergic reactions in diarrhea-allergic mice by increasing the Treg population [10], which suppressed allergen-specific IgE, basophil cells, MCs, and IL-4-produced by MCs. Moreover, peanut sublingual immunotherapy (SLIT) can induce desensitization, which may be mediated by decreased MC reactivity and increased IgG4 and IgA responses [11].

Shrimp allergen extract (SAE) is a potential immunotherapy agent for treating shrimp allergies. Though previous studies have used purified TPM or recombinant shrimp allergen [12–14], in this study, we used crude shrimp extract as an immunotherapeutic agent. The crude extract base in SAE is expected to extend the target for allergy tolerance induction, considering that TPM is not the sole allergen in shrimp [15]. In our prior study, we successfully established a gastro-food allergy model, and our findings showed that administering SAE in three different doses effectively achieved early desensitization in allergic mice. However, the mechanisms by which SAE induces immune tolerance are still not well established. Therefore, considering the need for an effective allergy cure, we evaluated the cellular and molecular changes in a gastro-food allergy mouse model owing to SAE desensitization. In this study, we found that SAE can induce immune tolerance even after the cessation of AIT administration, suggesting promising long-term effects. These findings support the emerging concept that SAE has the potential to cure seafood allergies.

## Methods

### Animals

Thirty female BALB/c mice (6–8 weeks) were obtained from the Animal Laboratory of the Faculty of Pharmacy at Airlangga University, Surabaya, Indonesia. All mice were fed a shrimp-free diet and maintained in a pathogen-free environment.

### SAE preparation

Vannamei shrimp (90–120 days) were used as the raw material in this study. Briefly, 500 g shrimp muscle was cut into pieces and ground. Next, 1:1 (w/v) acetone was used to completely dissolve the lipids and pigments. The extracts were then filtered and dried overnight in a fume hood (25°C) until the acetone was completely evaporated. Finally, the defatted samples were extracted in 1:10 (w/v) PBS pH 7.4 at 25°C. The total protein and TPM concentrations were determined at the end of the extraction process using the Coomassie Plus–Bradford Protein Assay Kit (Thermo Scientific #23236, Rockford, IL, USA) and Shrimp Tropomyosin 2.0 ELISA kit (InBio #EPC-TPM-1, Charlottesville, VA, USA), respectively, according to the manufacturer’s protocol. The extract was dissolved in 200 μL PBS by the dose specified for each animal group, containing 400, 100, 50, and 10 μg SAE.

### Proteomics sample preparation

100 μg shrimp protein extract was incubated with 2 M Urea and 10 mM TCEP in 50 mM ammonium bicarbonate (AmBic) for 1 h at 37°C and then alkylated with 15 mM iodoacetamide at R.T. in dark for 30 min. Subsequently, the protein solution was diluted with an equal volume of 50 mM AmBic and digested by 2 μg sequencing grade modified trypsin (Promega Corporation #V5111, Fitchburg, WI, USA) at 37°C overnight with agitation. After digestion, trifluoroacetic acid (TFA) was added to reach 1% (v/v) final concentration, and the resulting peptides were desalted by solid phase extraction using Pierce C18 Tips (Thermo Scientific #84850, Rockford, IL, USA) according to the manufacturer’s instructions. The purified peptides were dried by centrifugation under vacuum and reconstituted in 2% acetonitrile and 0.1% formic acid (FA). Peptide content was measured by the absorbance of 280 nm.

### LC-MS/MS data acquisition

1 μg of reconstituted peptides were loaded into Thermo Acclaim PepMap C18 Reversed Phase Trap Cartridge, with a particle size of 5 μm and dimensions of 0.3 mm I.D. x 5 mm length (Thermo Scientific #164560, Sunnyvale, CA, USA) and then separated by reverse phase chromatography using nano-LC column from PepSep ReproSil-Pur 120 C18-AQ a particle size of 1.9 μm and dimensions of 75 μm I.D. x 40 cm length (Dr. Maisch #r119.aq.s0740, Baden-Wuerttemberg, Germany). Peptides were eluted from 95% solvent A (0.1% FA) and 5% solvent B (80% acetonitrile, 0.1% FA) to 60% solvent A and 40% solvent B over a 90 min gradient at a flow rate of 300 nl/min by Ultimate 3000 RSLC chromatography system (Thermo Scientific, Germering, Germany). The eluted peptides were ionized by online nano-electrospray and measured by Orbitrap Exploris 480 Mass Spectrometer (Thermo Scientific, San Jose, CA, USA). Data dependent acquisition (DDA) mode was performed to obtain full MS1 scans from 385 m/z to 1540 m/z at a resolution of 120,000 at m/z 200. The cycle time between two MS^1^ full scans were set at less than 2 seconds. Precursor ions at +2 to +6 charge states were selected for fragmentation by HCD at 30% normalized collision energy and the product ions were analysed at a resolution of 15,000 at m/z 200. Ions with unassigned charge state were excluded. Dynamic exclusion of the same precursor ions was set to 20 s. Ion accumulation time for MS^1^ scan was set to auto and a maximum of 45 ms was set for data dependent MS^2^ scan.

### MS database search analysis

The MS data acquired were processed by PEAKS 11 (Bioinformatics Solutions Inc., Waterloo, ON, Canada). The reference proteome of *Litopenaeus vannamei* also known as Whiteleg shrimp (Proteome ID UP000283509) was sourced from the UniProt database on September 13^th^, 2023, and a total of 25,399 entries were acquired with genome accession QCYY01000000. The search criteria were set as follows: Mass error tolerance at 10 ppm for precursor ions, 0.02 Da for fragment ions; one-end tryptic specificity was required (semi-specific) with maximum 3 missed cleavages were allowed; carbamidomethylation at cysteines was set as fixed modification; acetylation at N-termini of the peptides, oxidation at methionines and deamidation at asparagines and glutamines were set as variable modifications. Peptide Spectral Matches (PSMs) were validated using a Target Decoy PSM Validator node, with q-values at a False Discovery Rate (FDR) of ≤1%. FDR for protein groups identified was set at 1%. Allergen visualization was built using SWISS-MODEL (https://swissmodel.expasy.org/interactive, accessed on July 17^th^, 2024). Allergen nomenclatures were obtained from the WHO/IUIS Allergen Nomenclature Sub-Committee (https://allergen.org/search.php?allergensource=Litopenaeus+vannamei, accessed on July 17^th^, 2024).

### Experimental design

The development of the gastro-food allergy mouse model has been described in previous studies [16, 17]. **Fig 3A** depicts the protocols for sensitization, challenge, and immunotherapy. The mice were divided into five groups (n = 6 per group). In the sensitization stage, the mice (n = 24) were sensitized intraperitoneally (i.p.) with 1 mg aluminum hydroxide (alum) and 100 μg SAE in PBS on days 0, 7, and 14 and randomly divided into four groups of six: a negative control (NC) and high- to low-dose immunotherapy. The non-sensitized (untreated) group (n = 6) received 1 mg alum in PBS without SAE (i. p.). To validate the development of a gastro-food allergy model in mice, a repeat intragastric (i.g.) challenge using 400 μg SAE was administered to all groups on days 21, 23, and 25, followed by systemic allergy symptom observation for 30 min. In the immunotherapy stage, SAE-sensitized mice from the immunotherapy groups were treated (i.p.) with 100 μg SAE for high-dose (HI), 50 μg SAE for moderate-dose (MI), and 10 μg SAE for low-dose (LI) on days 32, 39, and 46. Untreated and NC groups were administered PBS without SAE (i.p.). All mice were challenged with 400 μg SAE (i.g.) on days 53 and 58, followed by systemic allergy symptom observation, to assess the success of AIT in mice. Finally, all mice were euthanized using ketamine hydrochloride (PT. Guardian Pharmatama, Bogor, Indonesia) on day 59, and blood serum and ileum tissues were collected.

### Systemic allergy symptom assessment

The following scoring system was used to assess systemic allergy symptoms for 30 minutes after the challenge [16, 17]: 0 = no symptoms; 1 = scratching and rubbing around the nose and head; 2 = puffiness around the eyes, reduced activity with or without increased respiratory rate; 3 = wheezing, labored respiration, cyanosis around the mouth and tail; 4 = no activity after producing tremors and convulsions; and 5 = death.

### Serum IL-4 level analysis using ELISA

After mice were euthanized, blood was collected intracardially (open blood collection). Serum samples were obtained after centrifugation for 10 min at 4°C and 10,000 rcf, and the supernatant was collected. Each serum sample was analyzed for IL-4 levels using an ELISA Mouse IL-4 Kit (Elabscience #E-EL-M0043, San Diego, CA, USA) according to the manufacturer’s instructions.

### FcεR1α,Foxp3, IL-4, and IL-10 mRNA relative expression analysis using RT-qPCR

The ileal specimens were collected, immediately frozen in liquid nitrogen, and stored at –80°C until use. Total RNA was extracted from the ileum using an RNA Purification Kit (Jena Bioscience #PP-210S, Jena, Germany) and cDNA was synthesized using the GoScript Reverse Transcription System (Promega Corporation #A2791, Madison, WI, USA). RT-qPCR was carried out using the MyGo Mini device at 95°C for 3 min, followed by 40 cycles of 95°C to 63°C for 30 s and 60°C to 97°C for 1 min. In addition, the housekeeping gene β-actin was amplified in each sample for normalization between samples. The following primers were used (all Thermo Fischer Scientific, Waltham, MA, USA) [18–22]: *FcεR1α* (Forward: 5′-TGAATGACAGTGGCACCTACCA-3′, Reverse: 5′-CAGAATCGCCACCAACAATG-3′); *Foxp3* (Forward: 5′-CCCATCCCCAGGAGTCTTG-3′, Reverse: 5′-ACCATGACTAGGGGCACTGTA-3′); IL-4 (Forward: 5′-TCGGCATTTTGAACGAGGTC-3′, Reverse: 5′-CTGTGGTGTTCTTCGTTGCTG-3′); IL-10 (Forward: 5′-CAGTACAGCCGGGAAGACAATA-3′, Reverse: 5′-GCATTAAGGAGTCGGTTAGCAG-3′); β-actin (Forward: 5′-TTCTTGGGTATGGAATCCTGT-3′, Reverse: 5′-AGCACTGTGTTGGCATAGAG-3′).

### Degranulated MC histopathology

The ileum tissue was fixed with Carnoy’s solution, prepared as described in previous studies [23]. The tissues were initially submerged in paraffin and washed twice for 3 min with xylene. Next, the paraffin blocks were sliced to 5 μm thick sections. MCs were stained with 0.5% toluidine blue (TB) (Merck Millipore #104172, Darmstadt, Germany) to detect degranulation. Two lab members independently scored and compared the results for an objective assessment. The number of MCs in the ileum was measured in 10 microscopic fields randomly selected from six mice per group. The TB-positive cells with blurred cell membrane boundaries, increased cell membrane shrinkage, and five or more stained granules entirely scattered around the cells were considered degranulated MCs. In contrast, intact MCs have several viscous intracellular granules that stain intensely with TB and appear violet in the cytoplasm [24]. The percentage of degranulated MCs was calculated as follows:

$$Percentage\;of\;degranulated\;MCs=\frac{total\;number\;of\;MCs-number\;of\;normal\;MCs}{total\;number\;of\;MCs}\times 100$$

All observations were made under a 400× magnification light microscope and images were captured with a digital microscope camera at 1000× magnification (OPTIKA, B-190TBPL; Digital Binocular Microscope, Italy).

### Statistics

All data are presented as the mean ± SEM and statistically analyzed using one-way analysis of variance (ANOVA) or Kruskal–Wallis test, followed by a post hoc test using GraphPad Prism version 10.3.1 (GraphPad Software, San Diego, CA, USA). A p-value≤0.05 was considered the limit of statistical significance.

### Animal ethics approval

All animal protocols were approved by the Ethics Committee of the Faculty of Veterinary Medicine, Airlangga University, Surabaya, Indonesia, in April 2022 (Approval number 2.KEH.034.04.2022).

## Results

### SAE characterization

**Fig 1:**
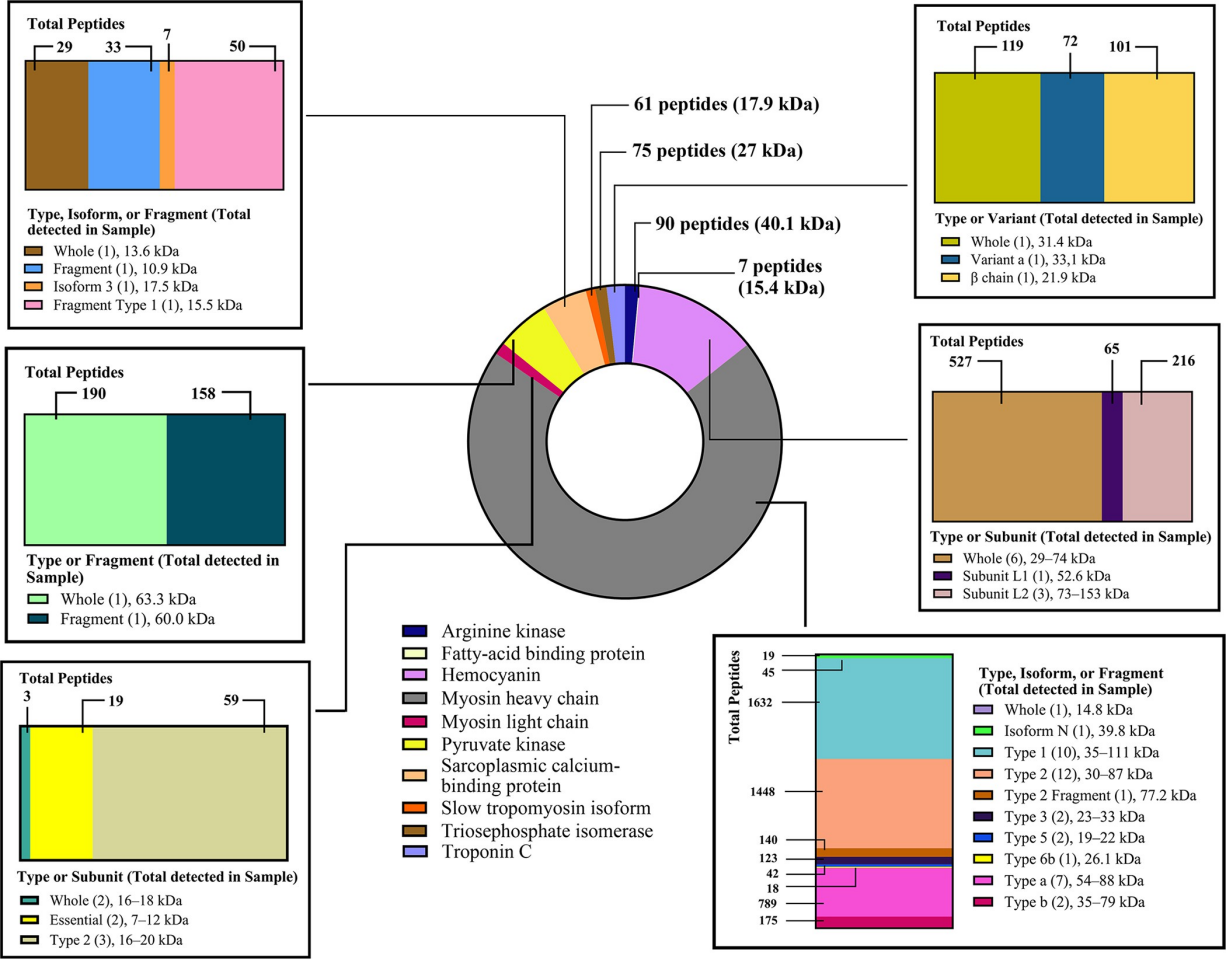
Allergen identification in shrimp allergen extract (SAE). A total of ten known allergens were identified in SAE. TPM, the primary shrimp allergen, is recognized in its isoforms.

Using MS analysis, ten known allergens (0.94%) out of a total of 1059 proteins were identified in SAE (**S1 Dataset**). These allergens include arginine kinase (AK or *Lit v 2*), fatty-acid binding protein (FAP or *Lit v 13*), hemocyanin (Hc), myosin heavy chains (MHCs), myosin light chains (MLCs, *Lit v 3* for type 2), sarcoplasmic calcium-binding protein (SCP or *Lit v 4*), pyruvate kinase (PK), slow TPM isoform (*Lit v 1*), triosephosphate isomerase (TPI), and troponin C (TnC). The most crucial allergen in shrimp, TPM, was found to have an allergenic potential of 22.5–82.8% [25]. Therefore, we conducted a comprehensive analysis of TPM content in SAE. We demonstrated that the TPM isoform in SAE had an average mass of 17.9 kDa and was composed of 61 peptides (**Fig 1**). We also found that 1 mL of the shrimp extract contains 3.5–4.5 mg of protein and 100–300 ng of TPM.

Post-translational modification (PTM) refers to modifications of amino acid side chains that occur to one or more amino acids in a protein following its biosynthesis [26, 27]. In the present study, we identified that TPM in SAE underwent PTMs at seven specific sites, involving three amino acids (AAs), in **Table 1**. The AAs involved were asparagine (N), glutamine (Q), leucine (L), and methionine (M). The identified PTMs included acetylation (N-Term), deamidation (NQ), and oxidation (M). The most prevalent modification observed on the TPM of SAE was deamidation (NQ), which is linked to protein degradation. Specifically, NQ, an irreversible and non-enzymatic PTM [26], was found at sites N55, N68, N97, Q94, and Q103. However, a complicating factor is that deamidation can occur as an experimental artifact during sample processing, making the detection of this PTM potentially unreliable. Similarly, acetylation (N-Term), which relates to protein-protein interactions [27], was found at site L95, and this might be a spontaneous modification after proteolysis of TPM. Additionally, oxidation (M), a spontaneous PTM associated with oxidative stress [26], was found at site M50.

**Table 1:** Sites and types of TPM modifications in SAE identified using proteomic approach.

PTM	Δ Mass (Da)	AA	AA Position
Acetylation (N-Term)	42.01	Leucine (Leu)	L95
Deamidation (NQ)	15.99	Asparagine (Asn)	N97
			N55
			N68
		Glutamine (Gln)	Q94
			Q103
Oxidation (M)	0.98	Methionine (Met)	M50

### SAE immunotherapy reduced systemic allergy symptoms in the allergic mice

**Fig 2:**
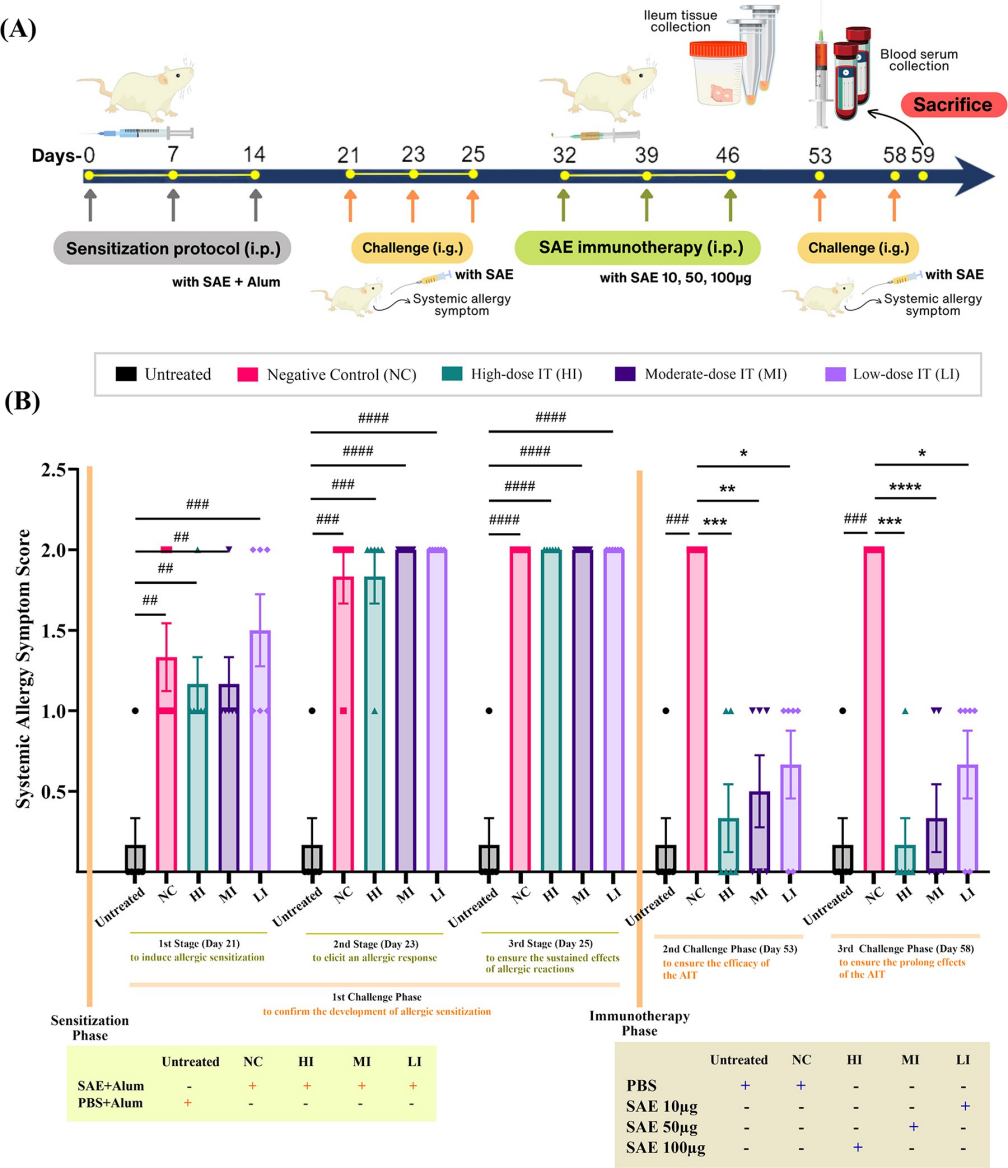
Shrimp allergen extract (SAE) immunotherapy affects systemic allergy reactions in allergic mice. (A) Experimental protocol. (B) The desensitized mice after SAE immunotherapy on days 53 and 58. Systemic allergy symptoms were observed for 30 minutes following challenge with 400 μg SAE (i.g.). SAE immunotherapy reduced systemic allergy symptoms in allergic mice on day 53 and showed a sustained effect until day 58. Each bar represents the mean ± SEM (n = 6 mice per group). p-values were derived from Kruskal-Wallis test (^####^, p≤0.0001; ^###^, p≤0.001; ^##^, p≤0.01 were significant against Untreated and ***, p≤0.001; **, p≤0.01; *, p≤0.05 were significant against NC).

Following the SAE challenge, systemic allergy symptoms were observed and scored (**Fig 2A**). **Fig 2B** shows that the sensitized mice experienced substantially stronger systemic allergy symptoms (p≤0.01 for the NC, HI, and MI groups; p≤0.001 for the LI groups) from the untreated (i.e. non-sensitized) mice after the first challenge on day 21. Repeated challenges also revealed a considerably higher incidence of systemic allergy symptoms in the sensitized mice than that in the untreated on day 23 (p≤0.001 for the NC and HI groups; p≤0.0001 for the HI and LI groups) and day 25 (p≤0.0001 for all sensitized mice). During the immunotherapy phase, the systemic allergic symptoms were significantly different (p≤0.001) between the negative control (i.e. sensitized but not treated with AIT) and untreated after challenge administration on days 53 and 58. Furthermore, systemic allergic symptoms were significantly reduced (p≤0.001, p≤0.01, and p≤0.05 for the high to low dosages) in all SAE immunotherapy-treated mice compared to the negative control mice on both days, indicating that the effects of SAE immunotherapy were sustained.

### SAE immunotherapy decreases FcεR1α mRNA relative expression in the ileum tissue

**Fig 3:**
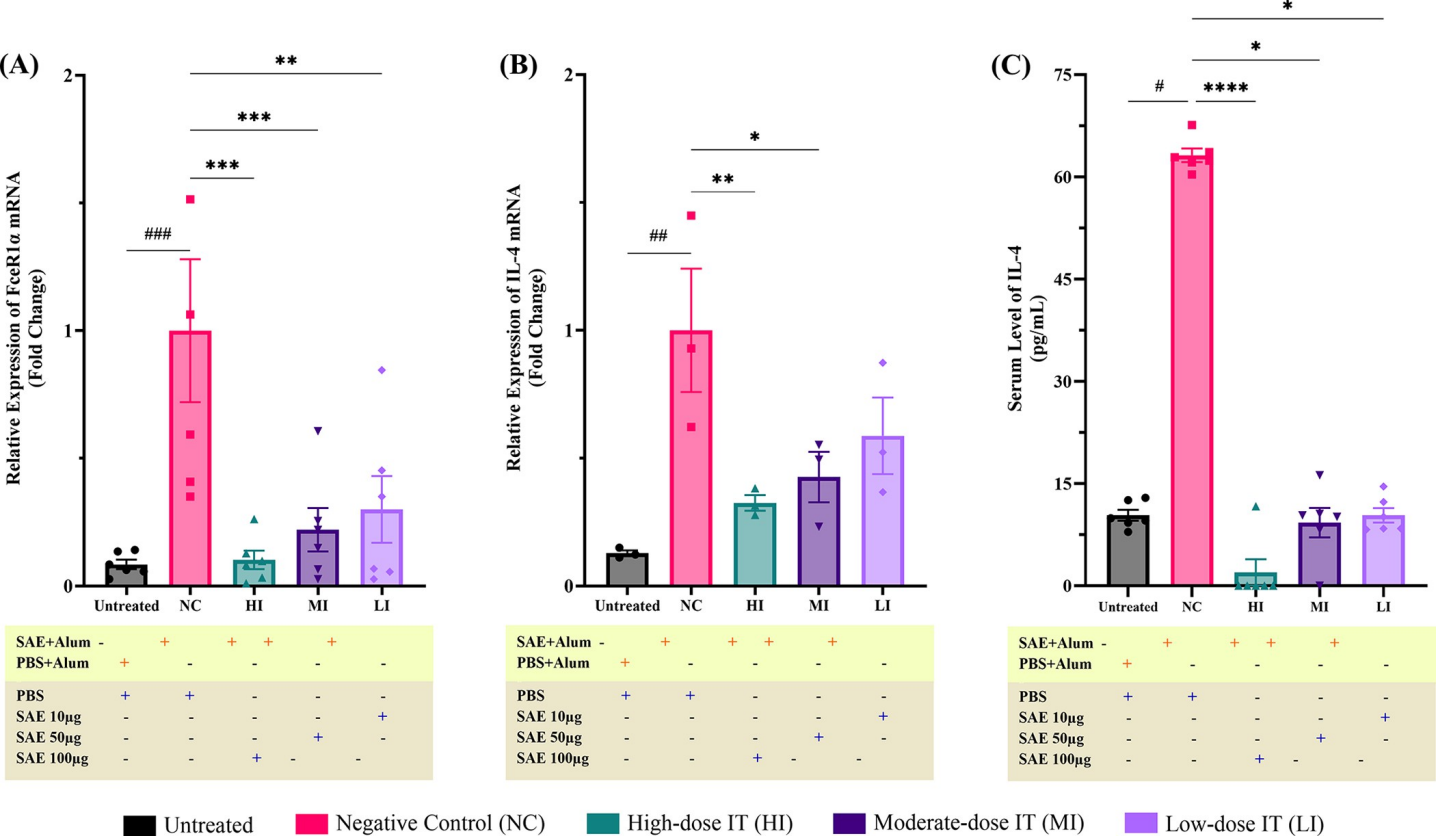
Shrimp allergen extract (SAE) immunotherapy preventing the development of allergies. Sensitized mice, but not the NC group, were treated (i.p.) with high- to low-dose immunotherapy (10 μg SAE for LI, 50 μg SAE for MI, and 100 μg SAE for HI). All mice were euthanized on day 59; blood serum and ileum tissues were collected and stored at −80°C for further analysis. SAE desensitization successfully decreased transcription of (A) *FcεR1α* mRNA (n = 6 mice per group) and (B) IL-4 mRNA—relative to NC—in the ileal tissue (n = 3 mice per group), and suppressed the production of the pro-inflammatory cytokine (C) IL-4 in serum (n = 6 mice per group). Each bar represents the mean ± SEM. p-values of *FcεR1α* and IL-4 mRNA expression were derived from one-way ANOVA test (^###^, p≤0.001; ^##^, p≤0.01; were significant against Untreated and ***, p≤0.001; **, p≤0.01; *, p≤0.05 were significant against NC) while p-values of IL-4 serum levels were derived from Kruskal-Wallis test (#, p≤0.05 were significant against Untreated and ****, p≤0.0001; *, p≤0.05 were significant against NC).

Cross-linking between *FcεR1* and the allergen–IgE complex initiates adverse allergic reactions because *FcεR1* activation induces MC degranulation and release of various inflammatory mediators [5–7]. Therefore, increased *FcεR1* mRNA expression in tissues has been associated with ongoing gastrointestinal mucosal inflammation [28]. The *FcεR1α* mRNA relative expression in ileum tissue from the negative control mice was significantly higher (p≤0.001) than that in the untreated (**Fig 3A**). However, the *FcεR1α* mRNA relative expression in the SAE immunotherapy-treated mice was significantly lower than in the negative control mice in a dose-dependent manner (**Fig 3A**).

### SAE immunotherapy reduces degranulated MCs in the ileum tissue

**Fig 4:**
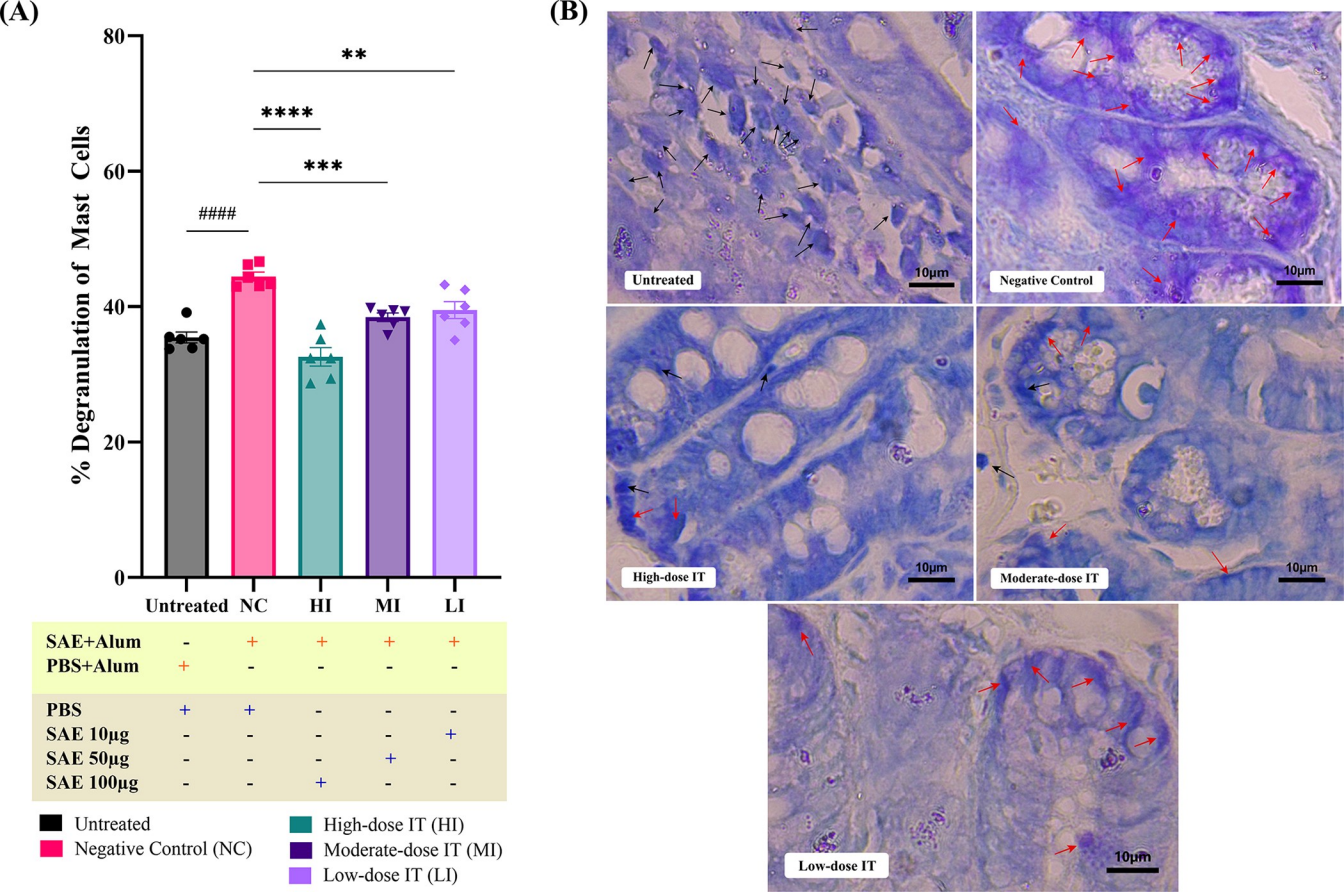
Shrimp allergen extract (SAE) immunotherapy affects mast cell (MC) degranulation in allergic mice. Sensitized mice, but not the NC group, were treated (i.p.) with high- to low-dose immunotherapy (10 μg SAE for LI, 50 μg SAE for MI, and 100 μg SAE for HI). All mice were euthanized on day 59; ileum tissues were collected and preserved in Carnoy’s solution. Degranulated MCs were stained using 0.5% toluidine blue (TB), and (A) the percentage of degranulated MCs in the intestinal tissues were determined. Each bar represents the mean ± SEM (n = 6 mice per group). p-values were derived from one-way ANOVA test (^####^, p≤0.0001 were significant against Untreated and ****, p≤0.0001; ***, p≤0.001; **, p≤0.01; were significant against NC). (B) Representative MCs in ileum specimens (1000× magnification). Degranulated MCs have blurred cell membrane boundaries and increased cell membrane shrinkage and granules scattered around the cells (red arrow). Intact MCs have several viscous intracellular granules that stain intensely with TB and appear violet in the cytoplasm (black arrow).

To demonstrate gastrointestinal mucosal inflammation, we examined the effect of SAE immunotherapy on the number of degranulated MCs (**Fig 4B**) in the ileal tissues of mice using histochemistry. The findings revealed that the number of degranulated MCs in the desensitized mice was significantly lower (p≤0.0001, p≤0.001, and p≤0.01 for the high to low dosages, respectively) than in the negative control mice (**Fig 4A**).

### SAE immunotherapy reduces IL-4 mRNA relative expression in the ileum tissue and IL-4 production in serum

IL-4 is the first inflammatory response-stimulating cytokine produced by MCs. IL-4 production by MCs has been extensively studied and is associated with IgE-mediated activation [5–7]. In line with our previous findings with immunoscoring (**Fig 2B**), the negative control mice had significantly higher (p≤0.001) IL-4 mRNA relative expression in ileum tissue (**Fig 3B**), followed by higher (p≤0.05) serum IL-4 levels (**Fig 3C**) than the untreated group. Conversely, SAE immunotherapy administration to allergic mice significantly decreased IL-4 mRNA relative expression (p≤0.001 for the HI and p≤0.05 for the MI) and IL-4 production (p≤0.0001, p≤0.05, and p≤0.05 for the high to low dosages) dose-dependently the negative control mice (**Fig 3B and 3C**).

### SAE immunotherapy increases Foxp3 mRNA relative expression in the ileum tissue

**Fig 5:**
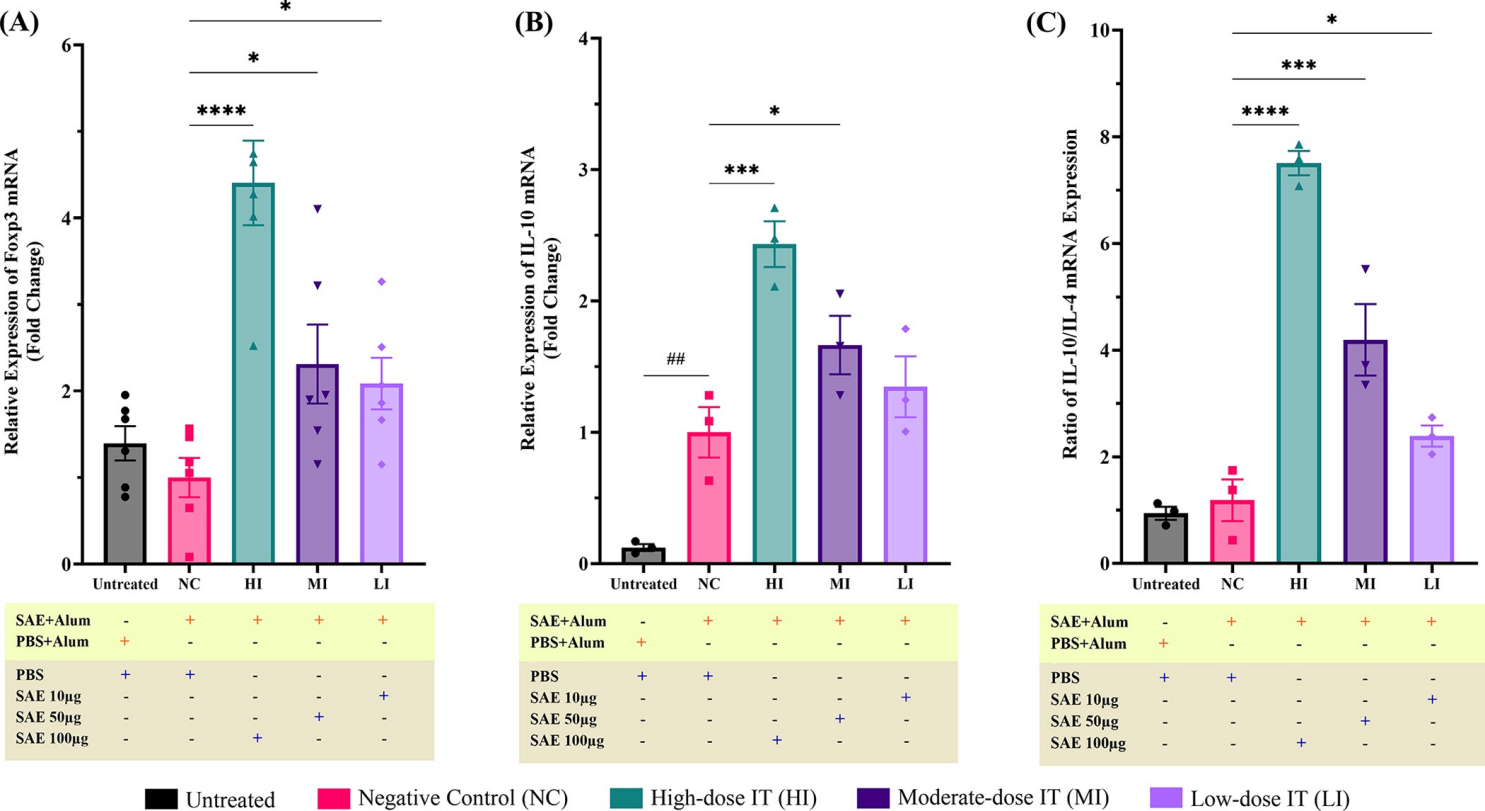
Shrimp allergen extract (SAE) immunotherapy inducing allergy tolerance. Sensitized mice, but not the NC group, were treated (i.p.) with high- to low-dose immunotherapy (10 μg SAE for LI, 50 μg SAE for MI, and 100 μg SAE for HI). All mice were euthanized on day 59; ileum tissues were collected and stored at −80°C for further analysis. SAE desensitization successfully increased transcription of (A) *Foxp3* mRNA (n = 6 mice per group) and (B) IL-10 mRNA (n = 3 mice per group)—relative to NC—in the ileal tissue. An increased (C) IL-10/ IL-4 mRNA ratio indicates a shift towards a stable tolerogenic phenotype following SAE desensitization. Each bar represents the mean ± SEM. p-values were derived from one-way ANOVA (^##^, p≤0.01; were significant against Untreated and ****, p≤0.0001; ***, p≤0.001; *, p≤0.05 were significant against NC).

*Foxp3* is an essential gene regulating Treg cell development. *Foxp3* mRNA expression levels are linked to Treg cell functions and immune tolerance [5, 9]. In this study, desensitized mice showed a significant dose-dependent increase (p≤0.0001, p≤0.05, and p≤0.05, respectively) in the *Foxp3* mRNA relative expression compared to the negative control mice (**Fig 5A**). The *Foxp3* mRNA relative expression in the negative control mice was lower than that in the untreated mice, although this difference was not statistically significant (p = 0.436).

### SAE immunotherapy increases IL-10 mRNA relative expression in the ileum tissue

IL-10 is a crucial cytokine produced by Treg cells, playing an essential role in developing immune tolerance during AIT [29, 30]. Increased IL-10 mRNA expression has been shown to correlate with the regulation of Th2 cell responses, leading to the suppression of type 2 pro-inflammatory cytokines, such as IL-4 [31–33]. In this study, we found that SAE immunotherapy in allergic mice significantly increased IL-10 mRNA relative expression (p≤0.001 for the HI and p≤0.05 for the MI) dose-dependently compared to the negative control mice (**Fig 5B**). Moreover, we also observed a significant increase in the IL-10/IL-4 ratio in all SAE immunotherapy-treated mice (p≤0.0001 for the HI, p≤0.001 for the MI, p≤0.05 for the LI) dose-dependently compared to the negative control mice (**Fig 5C**).

## Discussion

AIT is a promising treatment for IgE-mediated food allergies. This strategy provides a long-term cure for allergic conditions, including food allergies, with few side effects by inducing allergen-specific immune tolerance or desensitization [5, 34]. Therefore, AIT has emerged as a safe and effective alternative therapy for individuals with allergies. In principle, AIT targets specific allergens and efficiently induces allergen-neutralizing antibodies [35]. However, several studies mentioned that AIT can suppress allergic immune responses even in a non-specific manner under certain conditions, thereby preventing new allergies development to non-targeted allergens [36–38]. Interestingly, a study on OVA-sensitized mice found that exposure to novel allergens, such as house dust mites (HDM), led to severe intestinal allergy development by inducing intestinal barrier dysfunction [39]. This finding strengthens the AIT mechanism, relying on the role of allergen specificity in inducing allergic tolerance.

In this study, we developed a standardized SAE containing 3.5–4.5 mg protein and 100–300 ng TPM per mL SAE. Integrating SAE with a crude extract base in AIT development can contribute to broadening the target of inducing allergy tolerance. Previous studies have shown that individuals with shrimp allergies are often sensitive to multiple allergens [40]. The study found that TPM was not the only allergen bound to serum IgE [40]. Specifically, 42.9% (9 out of 21) of subjects were reported to have TPM-specific IgE sensitization, while 52.4% (11 out of 21) had sensitization to other shrimp allergens such as SCP, AK, and Hc [40].

Based on a bottom-up proteomic analysis, we successfully identified ten shrimp allergens as listed in the WHO/IUIS Allergen Nomenclature Sub-Committee. As the primary shrimp allergen, TPM was identified in its isoform with a molecular weight of 17.9 kDa. Furthermore, we also found PTMs on TPM isoforms, which could biologically influence protein behaviors and properties [27]. Deamidation, N-acetylation, and oxidation are PTMs that might occur during processing or spontaneously in “old” proteins [41, 42]. Even if these types of PTMs are introduced later during sample preparations, they could still be relevant, particularly deamidation and methionine oxidation, as they could potentially increase or reduce the vaccine’s immunogenicity [43, 44]. In addition, these PTMs might also be present in dietary foodstuffs containing TPM [15, 26].

The impact of PTM on immunological recognition has been widely reported. Basically, the deamidation of asparagine and glutamine residues are mostly spontaneous PTMs [45–47]. Asparagine deamidation on six specific AA sites of recombinant protective antigen (rPA) in anthrax vaccines were reported to affect antigen uptake efficiency by antigen-presenting cells (APCs), impacting the protease activities involved in antigen processing (MHC binding) and presentation (TCR recognition) [46, 48]. This modification ultimately diminishes vaccine efficacy by altering the T cell epitopes’ ability to cell-cell interactions or T cell activation [46]. In contrast, studies on the murine autoimmune model of cytochrome C (Cyt C) peptide 90–104 showed an increased immunogenic effect after immunization with asparagine deamidated peptide 90–104, eliciting strong B and T cell autoimmune responses [43]. Interestingly, previous studies suggest that the differential of T cell immune responses to deamidated epitopes depends on the binding affinity of deamidation sites to HLA alleles, which is a highly donor-specific response [44, 49].

Moreover, the role of protein oxidation on methionine residues has been reported to modulate both cellular and humoral immune responses [50–52]. A recent study revealed that the spontaneous methionine oxidation of the immunodominant peptide epitope 369-YMDGTMSQV-377 in melanoma enhances CD8^+^ T cell activation [52]. Another study indicated that methionine oxidation on Fc-dependent interactions of IgG decreases the binding affinity to protein A, protein G, and FcRn, which in turn decreases the IgG antibodies half-life and the effector function of therapeutic antibodies [50, 51]. Indeed, these studies suggest that peptide modification approaches hold promising implications in vaccine development, particularly allergy immunotherapy, by improving immune responses. Meanwhile, acetylation (N-Term) is a widely implicated PTM involved in many biological processes, such as gene transcription, metabolism, signal transduction, and autophagy [53]. The present study has demonstrated the presence of acetylation (N-Term) modification on the L95 residue, which might be an artifact of the sample processing following the proteolytic digestion.

Based on the effectiveness of the immunotherapy agent, our preliminary studies successfully developed a gastro-food allergy model and conducted an initial evaluation of SAE administration. We found that administering SAE immunotherapy significantly reduced systemic allergic symptom scores, accompanied by increased IgG2a levels and relative IL-10 mRNA expression [16]. In the present study, we investigated the cellular and molecular changes involved in SAE desensitization to provide scientific evidence supporting the efficacy of SAE in treating shrimp allergy. Consistent with our preliminary findings [16], we showed systemic allergic symptom scores in allergic mice decreased after SAE immunotherapy. These findings are also aligned with those of previous studies reporting that the administration of AIT-containing purified TPM (i.p.) and recombinant shrimp allergen (i.g., or intradermal; i.d.) can reduce systemic allergic symptoms in allergic mice [12–14]. Although we noted differences in the severity of systemic allergic symptoms in our allergy model compared to previous studies [12], this may be due to the type of allergen and adjuvant used during the sensitization induction [54]. Additionally, our results are also consistent with a prior clinical study [55], which showed significant reductions in systemic allergic symptoms in patients with shrimp allergies after six months of SLIT-containing shrimp extract.

We also performed two consecutive challenges to determine whether this immunotherapeutic effect was sustained over time. Our findings showed a sustained reduction in the systemic allergic symptom score at all doses. These results suggest prolonged systemic allergic symptom suppression. This is consistent with the finding that the desensitization effect of administering white egg OIT in the egg-allergic mouse model was also maintained two weeks after immunotherapy was discontinued [56].

Moreover, we investigated the levels of several molecular markers to confirm our scoring of symptoms and understand the mechanisms underlying the effectiveness of allergic desensitization using objective biochemical parameters. Fc-epsilon receptor 1 (*FcεR1*) is a high-affinity IgE receptor expressed on MC surface. The α chain of *FcεR1* binds to IgE [5–7]. Increased *FcεR1* expression occurs due to IgE binding to *FcεR1* on MCs, which has been shown to protect receptors from internalization and degradation. The previous study discovered that *FcεR1α*^−/−^ bone marrow mast cells (BMMCs) had no surface expression of *FcεR1* before or after IgE administration and were unresponsive to the survival-enhancing effects of IgE. In contrast, wild-type BMMCs treated with 10 μg/mL IgE showed increased *FcεR1* surface expression and cell survival. This condition continued to increase concurrently up to 100 μg/mL IgE and could remain for several days following the IgE withdrawal [57, 58]. Similarly, we found an increased *FcεR1α* mRNA relative expression in the ileum tissue of sensitized mice, whereas this was not increased in the immunotherapy-treated mice. Therefore, our data suggest that administering SAE immunotherapy induces immune tolerance by lowering *FcεR1α* expression, likely due to increased IgG production caused by a reduced Th2 response and/or suppression of IgE production by Treg [5–7]. IgG can inhibit IgE effects through receptor-mediated inhibition and steric blockade. In receptor-mediated inhibition, the allergen binds to both *FCγRIIb*-bound IgG and *FcεR1*-bound IgE on the surface of MCs simultaneously. This interaction promotes the *FcγRIIb* cytosolic immunoreceptor tyrosine-based inhibition motifs (ITIMs) phosphorylation, neutralizing Syk and PIP3 intermediate signalings induced by *FcεR1* activation. Meanwhile, IgG covers IgE epitopes in the steric blockade by binding to the allergens before they reach *FcεR1*-bound IgE [59, 60].

Cross-linking between *FcεR1* and the allergen–IgE complex activates the MCs, which triggers degranulation accompanied by various inflammatory mediator release, eventually developing immunity-related allergic reactions [5–7]. Based on the histopathological analysis, we discovered that the percentage of degranulated MC was reduced in desensitized mice. The following findings support the results of a previous study [14], which demonstrated that subcutaneous immunotherapy (SCIT) with recombinant TPM reduced MC degranulation in TPM-sensitized mice. Another study also found that desensitized mice had a lower percentage of degranulated MC after ovalbumin OIT [10]. Therefore, SAE immunotherapy likely induces immune tolerance, leading to unresponsive MCs to allergens and decreasing allergic symptoms. Previous studies have shown that actin cytoskeleton shifts in desensitized MCs can inhibit IgE-dependent calcium influx, leading to increased internalization of IgE bound to the cell surface. This, in turn, prevents MC activation and reduces MC degranulation [61, 62].

To confirm the effect of MC activation on systemic immune responses, we evaluated the IL-4 mRNA relative expression in the ileum tissue and the IL-4 serum levels. The up-regulation of IL-4 is positively linked to the induction of *FcεR1* expression, a rise in MC granule content, and the accelerated growth of mature MCs and their committed progenitors [7, 63, 64]. Previous studies have indicated that AIT administration suppresses IL-4 mRNA expression and IL-4 production [65, 66]. Likewise, we observed a reduction in local IL-4 mRNA expression in desensitized mice, which was associated with lower IL-4 serum levels compared to sensitized mice. These findings correspond with our earlier results showing increased *FcεR1α* mRNA relative expression and MC activation in allergic conditions. The changes reflect the immune response switch from a Th2 profile to a balanced Th2/Treg profile, leading to the induction of allergic tolerance. Consequently, the observed decrease in IL-4 mRNA relative expression and IL-4 serum levels after SAE immunotherapy may be due to the role of Treg cells in diminishing proliferative and cytokine responses to allergens [5–7, 67–69].

Previous studies have reported that peripheral blood mononuclear cells (PBMCs) from patients with active cow’s milk allergy had considerably stronger proliferative activity against β-lactoglobulin after milk challenge than outgrown allergy patients with higher circulating CD4^+^CD25^+^ Tregs [68]. Additionally, comparable findings were observed in patients with allergic rhinitis and asthma [69]. Regarding those findings, we also investigated the levels of Treg cells as reflected by *Foxp3* expression, the main transcription factor of the Treg cell, after administering SAE immunotherapy.

AIT primarily aims to induce peripheral T cell tolerance to allergens by involving allergen-specific Treg cells. Several studies have shown that low Treg numbers are associated with the severity of food allergies [70–72]. *Foxp3* mRNA relative expression increases in Tregs and causes immunosuppression [73, 74]. The *Foxp3* mRNA relative expression increased in the mice that received SAE immunotherapy. This finding is consistent with previous research using an egg allergy model [75]. The study found that ovalbumin peptide-based immunotherapy effectively increased the *Foxp3* mRNA and TGF-β expression in the intestinal tissue [75]. Similarly, administration of HDM immunotherapy induced an antigen-specific suppressive activity in CD4^+^CD25^+^ Tregs of allergic individuals by involving TGF-β and IL-10 as the responsible suppressive pro-inflammatory cytokines [69]. In its suppressive role, TGF-β has a different action duration than IL-10. TGF-β exerts its suppressive effects later, typically between 12 and 16 hours post-stimulation. In contrast, IL-10 impressively acts much earlier -—approximately 3 hours post-stimulation—by promoting the degradation of cytokine mRNA [76]. In the context of allergy treatment, elevated IL-10 levels are crucial for mediating allergy tolerance by regulating Th2-driven allergic diseases.

Subsequently, we explored the involvement of IL-10 in mediating the induction of allergic tolerance by determining the relative expression of IL-10 mRNA in ileal tissue. Our findings showed that the elevated IL-10 expression persisted in all desensitized mice, even after the completion of immunotherapy. This highlights the encouraging success of achieving allergic tolerance through SAE immunotherapy. Similar efficacy of AIT has been reported in individuals with allergic rhinitis who were treated with received birch and timothy allergens [77]. This study reported that all patients experienced an increase in allergen-specific IL-10 mRNA expression after a year of SCIT [77]. Another study also reported that administering SLIT-containing Bet v 1, the major birch pollen allergen, for one month could cause an increase in the frequency of circulating CD4^+^CD25^+^ Treg cells along with an increase in *Foxp3* and IL-10, followed by a decrease in IL-4 and IFN-γ mRNA expression levels [31]. In this case, IL-10 may act directly on the Treg cells to help maintain *Foxp3* expression [78].

Moreover, IL-10 not only induces early T cell tolerance but also regulates specific antibody isotype formation, shifting the immune response from IgE to IgG4 in humans or IgG2a in mice [29–32, 79, 80]. This shift leads to the inhibition of MC activation and pro-inflammatory cytokine release by MCs [31, 33, 79, 81]. Previous studies indicated that IL-10 dramatically inhibited the expression of both germline and productive *ε* transcripts derived from IL-4 in PBMCs [79, 82]. These studies reported that IL-10 indirectly downregulates IgE by diminishing its accessory cells, which are responsible for IL-4-induced production of IgE and the expression of C*ε* germline transcripts (GLTs). Importantly, this effect does not impact IgG4 production or the expression of C*γ*4 GLTs in PBMCs. Additionally, IL-10 directly stimulates CD27^+^ B cells to enhance IgG4 production [82]. Furthermore, the autocrine secretion of IL-10 by myeloid dendritic cells (mDCs) has rapidly inhibited *FcεR1*-dependent pro-inflammatory responses [81]. Therefore, we evaluated the ratio of IL-10 mRNA to IL-4 mRNA after SAE immunotherapy to confirm its suppressive role in regulating inflammatory responses. We reported a significant increase in IL-10/IL-4 mRNA ratio in desensitized mice, suggesting a shift towards a stable tolerogenic phenotype of AIT.

**Fig 6:**
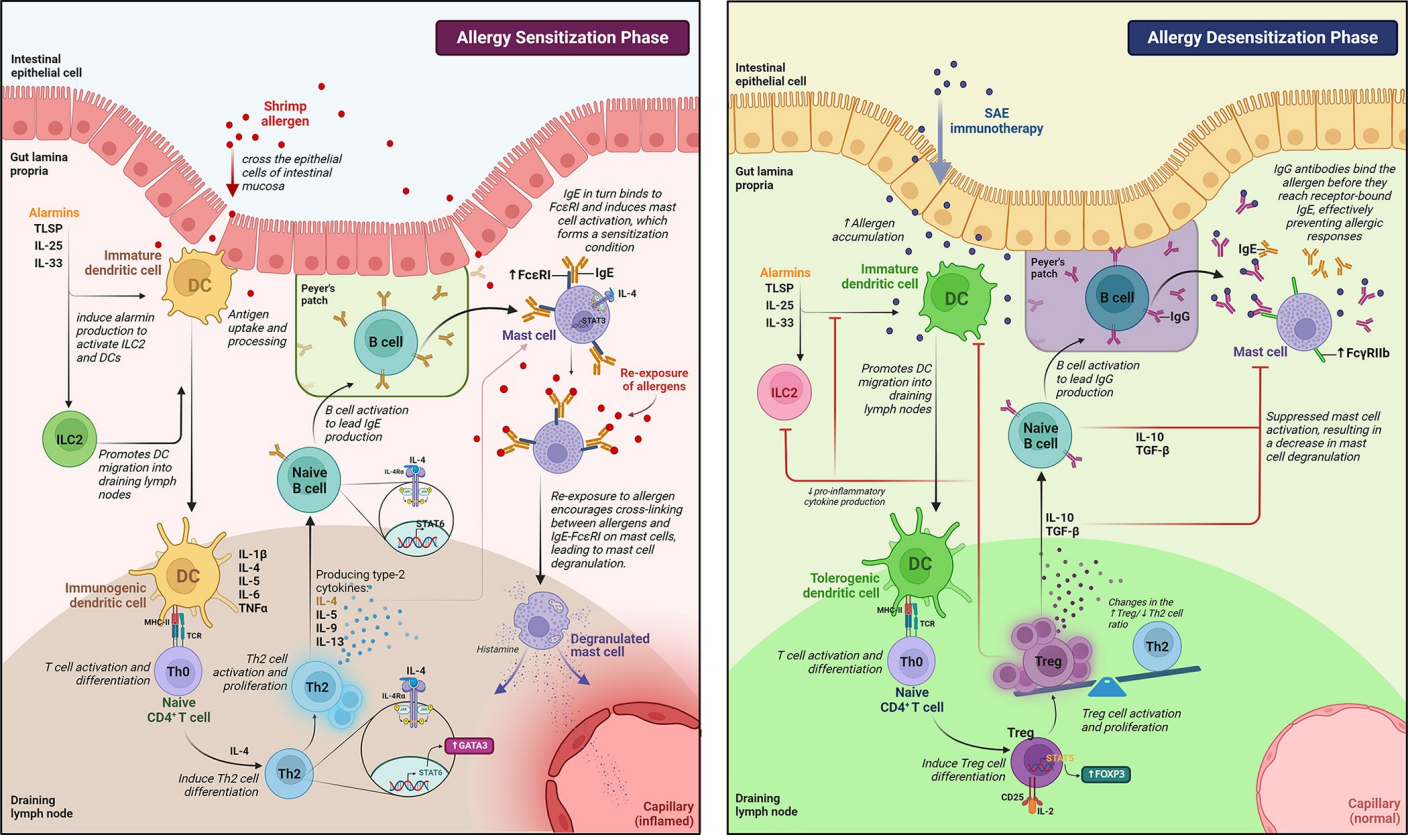
Schematic presentation of SAE immunotherapy-induced allergy tolerance in shrimp allergy. During the desensitization phase, SAE administration increases systemic allergen concentration, shifting the Treg/Th2 immune response. Subsequently, tolerogenic DC activates Treg cells through the transcription factor Foxp3. Activation of Treg cells induces IL-10 production, which inhibits IgE production from B cells, suppresses ILC2 activation via alarmins, and prevents cross-linking formation. This ultimately reduces MC degranulation and the release of pro-inflammatory cytokines by MCs.

In summary, SAE immunotherapy can desensitize a gastro-food allergy mouse model. This finding is based on the reduction in systemic allergic symptoms in allergic mice after SAE immunotherapy. The condition is subsequently confirmed by a decreased *FcεR1α* mRNA relative expression on the MC surface, which decreased the number of degranulated MCs and local and systemic IL-4 levels, followed by an increased *Foxp3* and IL-10 mRNA relative expression. These findings demonstrated that SAE immunotherapy has remarkable efficacy and safety in inducing prolonged immune tolerance with a wide range of allergen targets. The mechanism by which SAE immunotherapy induces immune tolerance is illustrated in **Fig 6**. Moreover, since AIT is not used in the standard care of food allergy therapy owing to the risk of anaphylaxis, this study provides scientific data establishing SAE as a promising therapeutic approach that can be used prophylactically to prevent food allergy development.

## Conclusion

This study concluded that SAE can effectively treat food allergies and has the potential to be developed as an immunotherapeutic agent by inducing allergy tolerance. Therefore, follow-up studies to further establish the safety and efficacy of SAE immunotherapy are warranted.

## Supporting information

S1 File (DOCX)

S1 Appendix Research method details. (DOCX)

S1 Dataset List of SAE proteins identified using a proteomic-based approach. (DOCX)

S2 Dataset **A.** Systemic Allergy Scores. **B.** Evaluation of FcεR1α mRNA Relative Expression. **C.** Degranulated Mast Cells Analysis. **D.** Evaluation of IL-4 mRNA Relative Expression. **E.** IL-4 Serum Level Analysis. **F.** Evaluation of Foxp3 mRNA Relative Expression. **G.** Evaluation of IL-10 mRNA Relative Expression. **H.** Evaluation of IL-10/IL-4 mRNA Ratio.
(DOCX)
