# Supplementary material for: Shrimp allergen extract immunotherapy induces prolonged immune tolerance in a gastro-food allergy mouse model
Source: PLoS One. 2024 Dec 27;19(12):e0315312. doi: 10.1371/journal.pone.0315312 (PMC11676511; doi:10.1371/journal.pone.0315312)
Supplement: S1 File — (DOCX) [file pone.0315312.s001.docx]

***S1 and S2 Figure***

**Shrimp allergen extract immunotherapy induces prolonged immune tolerance in a gastro-food allergy mouse model**

Honey Dzikri Marhaeny^1^, Lutfiatur Rohmah^1^, Yusuf Alif Pratama^1^, Salsabilla Madudari Kasatu^1^, Andang Miatmoko^2^, Rafi Addimaysqi^3^, Geert van den Bogaart^4^, Franz Y. Ho^5^, Muhammad Taher^6^, Junaidi Khotib^1*^

^1^Department of Pharmacy Practice, Faculty of Pharmacy, Airlangga University, Surabaya, Indonesia

^2^Department of Pharmaceutical Science, Faculty of Pharmacy, Airlangga University, Surabaya, Indonesia

^3^Faculty of Medicine, Airlangga University, Surabaya, Indonesia

^4^Department of Molecular Immunology and Microbiology, Groningen Biomolecular Sciences and Biotechnology Institute, Faculty of Science Engineering, University of Groningen, Groningen, The Netherlands

^5^GBB Proteomics, Groningen Biomolecular Sciences and Biotechnology Institute, Faculty of Science Engineering, University of Groningen, Groningen, The Netherlands

^6^Department of Pharmaceutical Technology, Kulliyyah of Pharmacy, International Islamic University Malaysia, Kuantan, Pahang, Malaysia

***Corresponding Author:**

Email: [junaidi-k@ff.unair.ac.id](mailto:junaidi-k@ff.unair.ac.id) (JK)

**S1 Fig.**


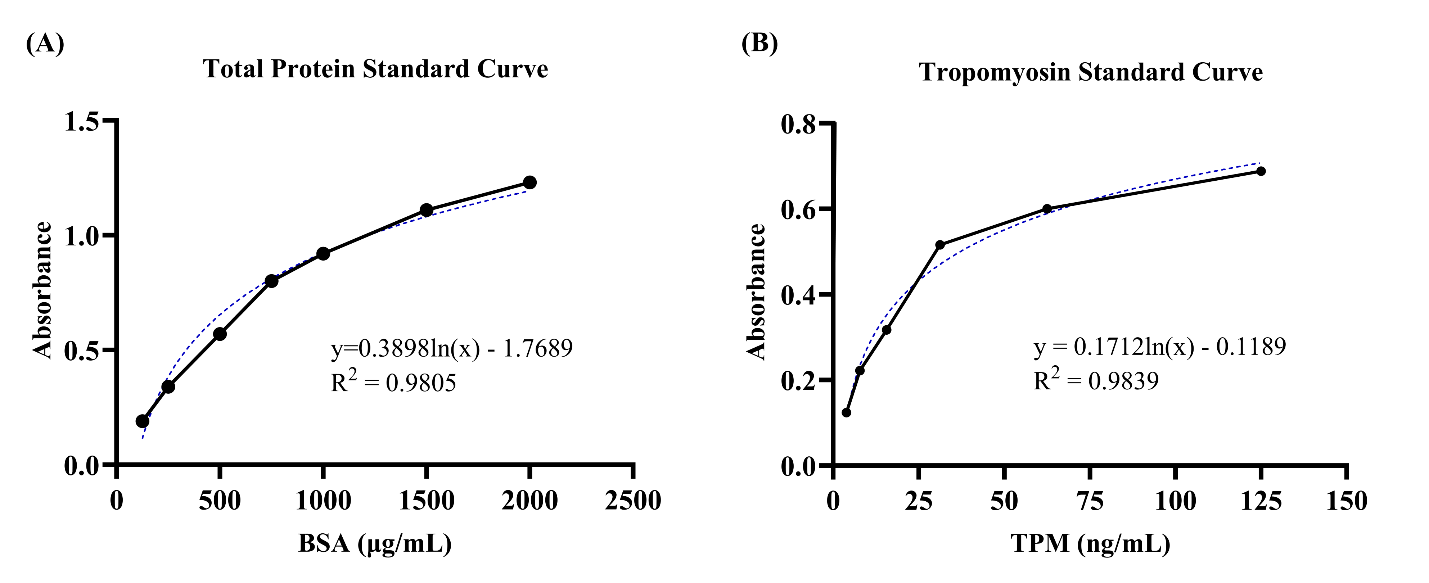


**S1 Fig.** Standard curve of (A) total protein (μg/mL) and (B) TPM (ng/mL). Total protein in shrimp allergen extract (SAE) was analyzed using the Bradford protein assay, while TPM was analyzed using ELISA.

**S2 Fig.**


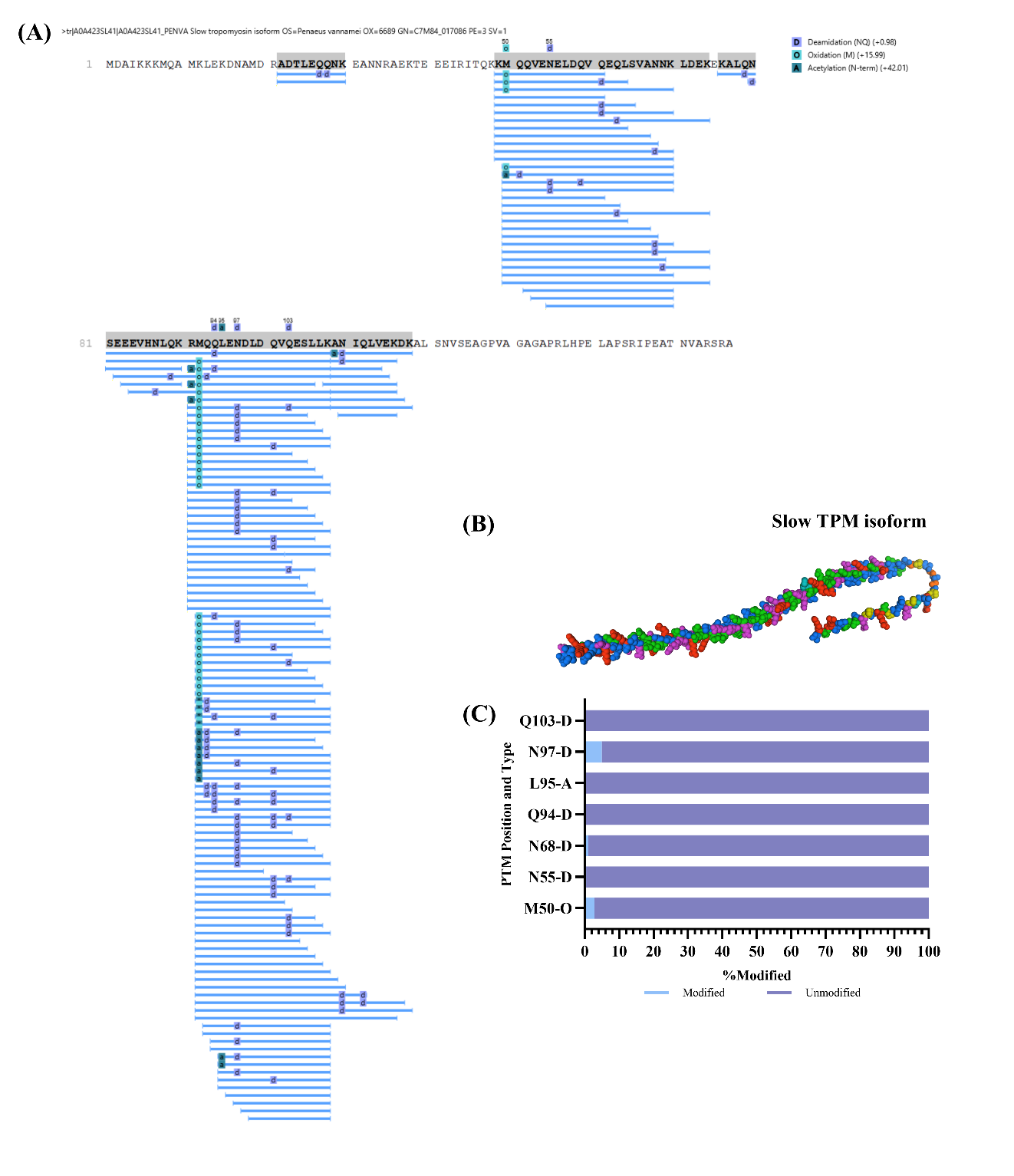


**S2 Fig.** PTM identification in TPM isoform of shrimp allergen extract (SAE). (A) Mapping of the AA confident modified sites in TPM isoform sequence. Accession number: A0A423SL41|A0A423SL41_PENVA. (B) Visualization of the slow TPM isoform structure using SWISS-MODEL. (C) Percentage of modified versus unmodified AAs at specific PTM sites. In this context, L, M, N, and Q denote the one-letter code for AAs, i.e., leucine, methionine, asparagine, and glutamine, respectively. A, D, and O denote the types of PTMs, i.e., acetylation (N-Term), deamidation (NQ), and oxidation (M), respectively.
