# Supplementary material for: Shrimp allergen extract immunotherapy induces prolonged immune tolerance in a gastro-food allergy mouse model
Source: PLoS One. 2024 Dec 27;19(12):e0315312. doi: 10.1371/journal.pone.0315312 (PMC11676511; doi:10.1371/journal.pone.0315312)
Supplement: S1 Appendix — (DOCX) [file pone.0315312.s002.docx]

***S1 Appendix***

**Shrimp allergen extract immunotherapy induces prolonged immune tolerance in a gastro-food allergy mouse model**

Honey Dzikri Marhaeny^1^, Lutfiatur Rohmah^1^, Yusuf Alif Pratama^1^, Salsabilla Madudari Kasatu^1^, Andang Miatmoko^2^, Rafi Addimaysqi^3^, Geert van den Bogaart^4^, Franz Y. Ho^5^, Muhammad Taher^6^, Junaidi Khotib^1*^

^1^Department of Pharmacy Practice, Faculty of Pharmacy, Airlangga University, Surabaya, Indonesia

^2^Department of Pharmaceutical Science, Faculty of Pharmacy, Airlangga University, Surabaya, Indonesia

^3^Faculty of Medicine, Airlangga University, Surabaya, Indonesia

^4^Department of Molecular Immunology and Microbiology, Groningen Biomolecular Sciences and Biotechnology Institute, Faculty of Science Engineering, University of Groningen, Groningen, The Netherlands

^5^GBB Proteomics, Groningen Biomolecular Sciences and Biotechnology Institute, Faculty of Science Engineering, University of Groningen, Groningen, The Netherlands

^6^Department of Pharmaceutical Technology, Kulliyyah of Pharmacy, International Islamic University Malaysia, Kuantan, Pahang, Malaysia

***Corresponding Author:**

Email: [junaidi-k@ff.unair.ac.id](mailto:junaidi-k@ff.unair.ac.id) (JK)

**Appendix A: Experimental Design**

***Animal Subjects***

Thirty female BALB/c mice (6–8 weeks) were obtained from the Animal Laboratory of the Faculty of Pharmacy at Airlangga University, Surabaya, Indonesia. The mice were housed in individually ventilated cages (IVCs) with a maximum of 2–3 mice per cage, and the cages were lined with corn cob bedding, which was changed every two or three days. The room temperature was maintained at 22 ± 2 °C, and the lighting followed a 12-hour light/dark cycle, starting from 06.00 AM to 06.00 PM. The facility was designed to minimize noise. All mice were provided with a shrimp-free diet, had access to fresh water *ad libitum*, and maintained in a pathogen-free environment. Before starting the experimental procedures, mice were allowed to acclimate to the environment for 14 days. During this period, they were monitored for signs of stress or illness. All personnel involved in handling and caring for the animals were trained according to institutional guidelines and regularly evaluated for competency. The animals' general health was observed at least once daily.

***Ethical Statement***

All procedures involving animals were conducted in accordance with the ethical standards of the Faculty of Veterinary Medicine, Airlangga University, Surabaya, Indonesia, in April 2022 (Approval number 2.KEH.034.04.2022). The study was designed to minimize animal suffering and use the fewest animals necessary for statistical significance.

***Induction of Food Allergy, Allergen Challenge and Monitoring, and Immunotherapy***

In this study, we used a 0.5 mL syringe for intraperitoneal (i.p.) treatment and a feeding tube for intragastric (i.g.) treatment. The mice were sensitized with 1 mg of aluminum hydroxide (alum) and 100 µg of shrimp allergen extract (SAE) in 200 µL of PBS pH 7.4 (i.p.) on days 0, 7, and 14. The untreated group received 1 mg of alum in 200 µL of PBS pH 7.4 without SAE (i.p.). Subsequently, the mice were challenged with 400 µg of SAE in 200 µL of PBS pH 7.4 (i.g.) on days 21, 23, and 25. After the challenge, the mice were monitored for clinical signs of an allergic reaction, such as scratching, decreased activity, and respiratory distress.

For the immunotherapy, we conducted an experiment with three groups of sensitized mice, each receiving a different dose of SAE in 200 µL of PBS pH 7.4 (i.p.) on days 32, 39, and 46. These groups, referred to as the immunotherapy groups, were administered high-dose (100 µg), moderate-dose (50 µg), and low-dose (10 µg) SAE. The untreated and negative control groups were given 200 µL of PBS pH 7.4 without SAE (i.p.). All mice were then challenged with 400 µg of SAE in 200 µL of PBS pH 7.4 (i.g.) on days 53 and 58, followed by observation of systemic allergy symptoms. Finally, all mice were euthanized on day 59, and blood serum and ileum tissues were collected.

**Appendix B: Systemic Allergy Symptoms and Scoring**

***Detailed Criteria for Systemic Allergy Symptoms***

1. **Scratching or rubbing** behavior is assessed when mice show several scratches for about 1 second. This series of behaviors is counted as one of the scratching or rubbing incidents. According to our validation results, each mouse is assigned a score of 1 if it has at least 15 incidents of scratches or rubs in the nose or head area within 5 minutes.
2. **Respiratory rate** is assessed by counting the times the chest rises and falls (1 full rise and fall equals one breath) in 15 seconds and multiplying by four to get the respiratory rate in breaths per minute. Healthy mice have a respiratory rate of around 80–230 breaths per minute.
3. The **shortness of breath** in mice is shown by observing their behavior of struggling to breathe or inhale and exhale normally. This condition can be marked by lowering their head, flaring their nostrils, and breathing with their mouth open. Mice also showed signs of gasping for air or rapid breathing. In addition, wheezing in mice sounds similar to wheezing in humans. When breathing, a high-pitched whistling sound typically occurs on an exhalation.

***Scoring System***

The scoring system is based on the most severe. For example, if the mouse exhibits scratching symptoms (score 1) and muzzle swelling (score 2), the systemic allergic symptoms score is 2.

**Appendix C: Efforts to Alleviate Suffering**

The following information describes the specific criteria, the time elapsed before euthanasia once criteria were met, and the occurrence of any unexpected deaths.

***Criteria for Humane Endpoints***

Animals should be euthanized if they meet one or more of the following criteria, which indicate severe distress or an irreversibly damaged condition:

1. **Weight Loss**

Animals were monitored daily, and any animal losing more than 20% of its initial body weight over a few days, despite supportive care, was considered for humane euthanasia.

1. **Clinical Signs of Severe Allergic Reaction**

Observed clinical signs included:

- - - **Respiratory Distress:** Labored breathing, wheezing, or gasping for air.
    - **Cyanosis:** Blue skin discoloration, particularly around the nose and extremities.
    - **Anaphylactic Shock:** Rapid onset of unresponsiveness, lethargy, or collapse.

1. **Inactivity and Mobility Issues**

Any animal showing prolonged immobility (e.g., unable to rise or move within its cage for more than 3 hours) or being unable to reach food and water sources was considered for euthanasia.

1. **General Health Deterioration**

Additional criteria included persistent diarrhea, dehydration, or a significant reduction in body temperature (0.5‐10°C).

***Time Elapsed Before Euthanasia***

Once animals met the humane endpoint criteria, they were euthanized within 30–45 minutes/hours to ensure timely alleviation of suffering. The time elapsed before euthanasia should be carefully recorded. In this study, we reported that **none of the experimental animals experienced severe symptoms that met the humane endpoint criteria.**

***Animals That Died Before Meeting Euthanasia Criteria***

In case an animal dies before meeting euthanasia criteria, the circumstances of the death should be thoroughly evaluated, and the monitoring protocols should be adjusted to minimize the risk of premature death in the future. In this study, we reported that **none of the experimental animals died unexpectedly.**

**Appendix D: Methods of Sacrifice and Sample Collection**

***Anesthesia and Euthanasia using Ketamine HCl***

Mice were anesthetized with 10 mg/100 µL of ketamine hydrochloride intraperitoneally. Anesthetic depth was confirmed by the absence of reflexes, such as the toe pinch and loss of the righting reflex. Once the animals were fully anesthetized and unresponsive to stimuli, a lethal dose of ketamine hydrochloride (200 mg/kg, intraperitoneally) was administered to induce euthanasia. Death was confirmed by the absence of a heartbeat, respiration, and lack of reflex response. Following euthanasia, animals were placed on ice for immediate sample collection. Blood samples and tissues were harvested under 7 minutes of euthanasia to ensure the integrity of the samples.

***Sample Collection***

Samples were collected at the designated time points as follows:

1. **Blood Collection:**

Blood samples were collected intracardially (open blood collection) using a 25-gauge needle under anesthesia. Approximately 1 mL of blood was collected into a 1.5 mL tube. Serum samples were obtained after centrifugation for 10 min at 4 °C and 10,000 rcf, and the supernatant was collected.

1. **Tissue Collection:**

Following euthanasia, the ileal tissues were collected, and the organ was carefully excised using sterile surgical instruments. The collected samples were rinsed with 0.9% NaCl and divided into two parts. The first part was immediately frozen in liquid nitrogen and stored at –80 °C until used for RT-qPCR analysis. Meanwhile, the other part was fixed and stored in a sealed container containing Carnoy's solution for histopathological evaluation.

**Appendix E:** **Statistical Analysis Validation**

***Normality Test and Decision of Selected Statistical Analysis Method***

The normality test was performed using the Shapiro-Wilk Test due to the relatively small sample size (<100). In the event of a normal distribution in the data, parametric statistical analysis (One-Way ANOVA) can be performed. Conversely, non-parametric statistical analysis (Kruskal-Wallis Test) will be used for non-normally distributed data. Systemic allergy symptom assessment data (ordinal data) were analyzed using the Kruskal-Wallis Test. All analyses were performed using GraphPad Prism version 10.3.1 (GraphPad Software, San Diego, CA, USA). A p-value < 0.05 was considered statistically significant.

***Validation Results of Data 1:***


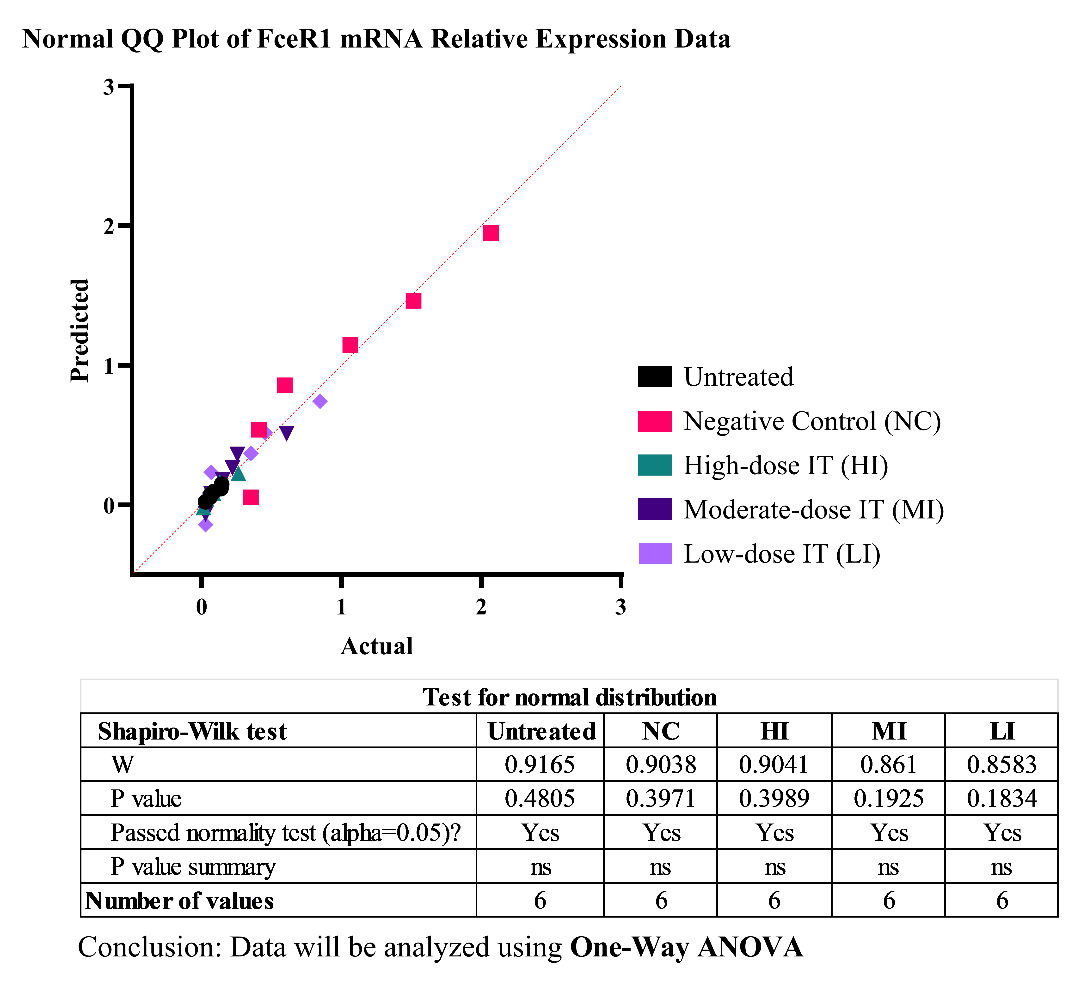


***Validation Results of Data 2:***

**
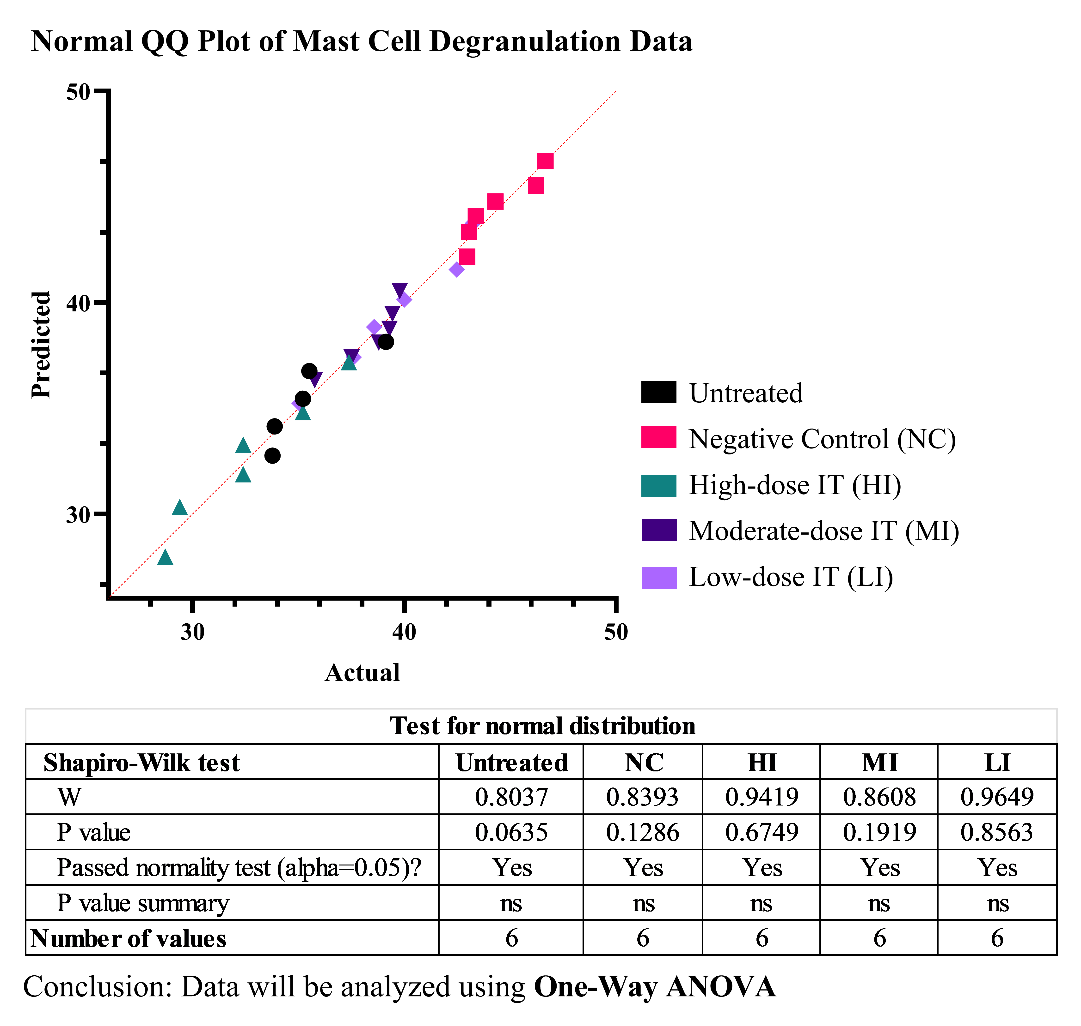
**

***Validation Results of Data 3:***

**
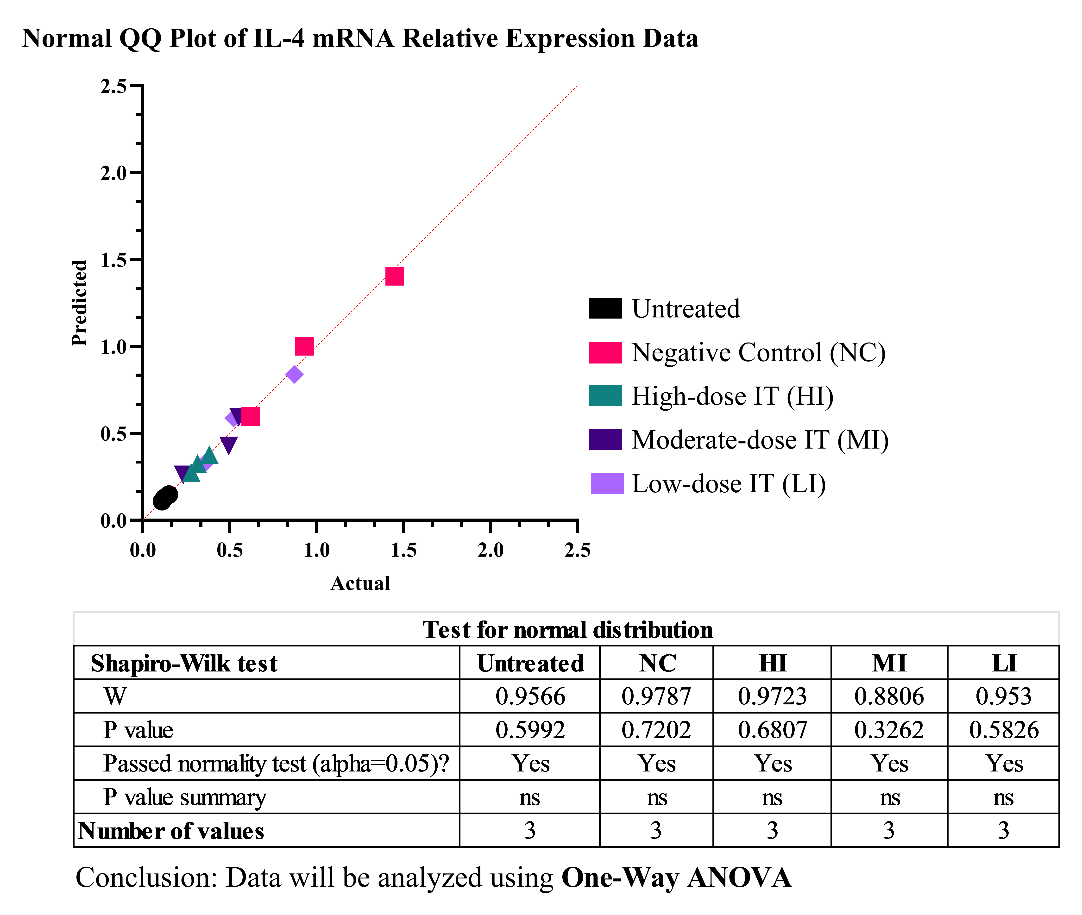
**

***Validation Results of Data 4:***


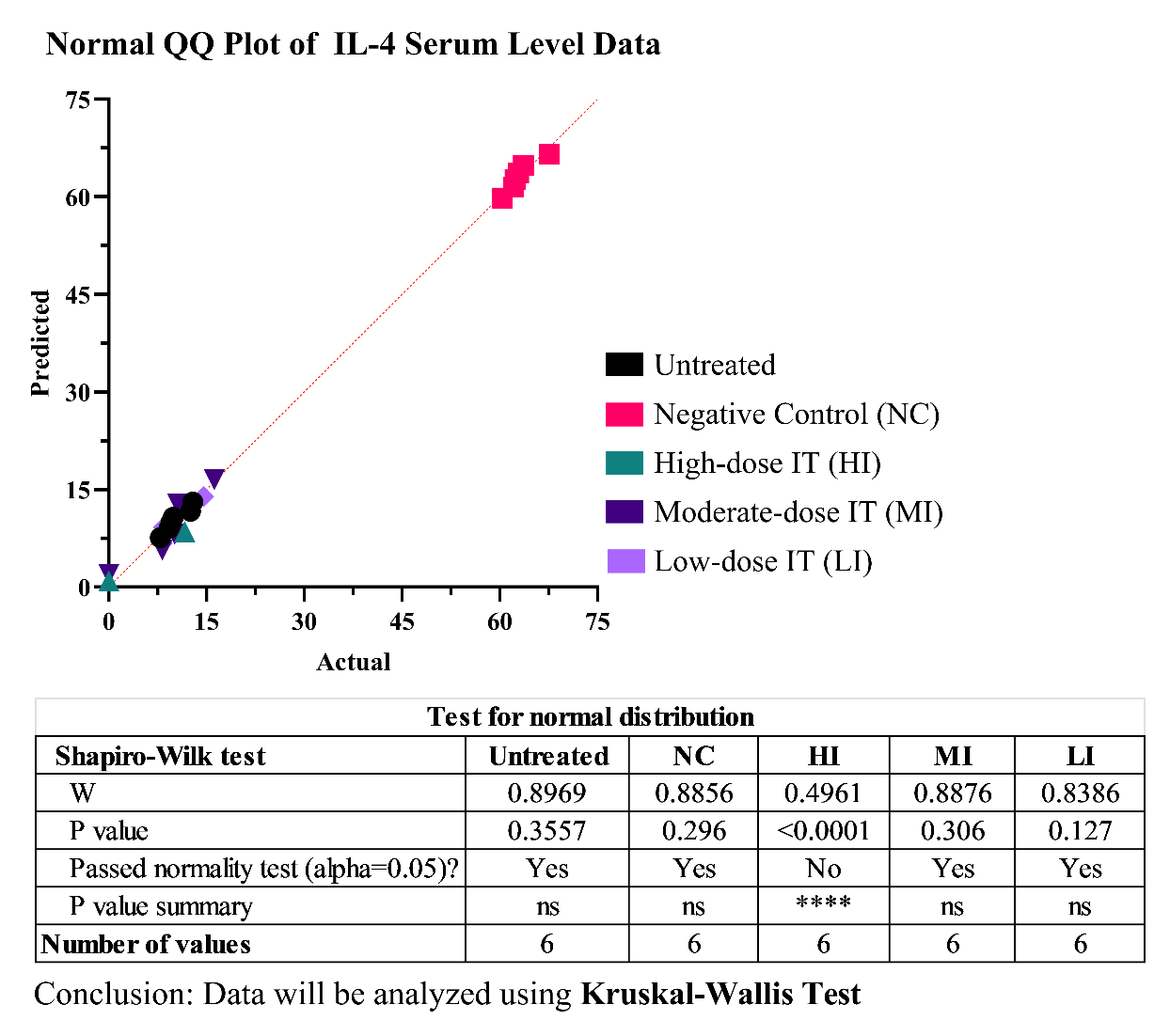


***Validation Results of Data 5:*
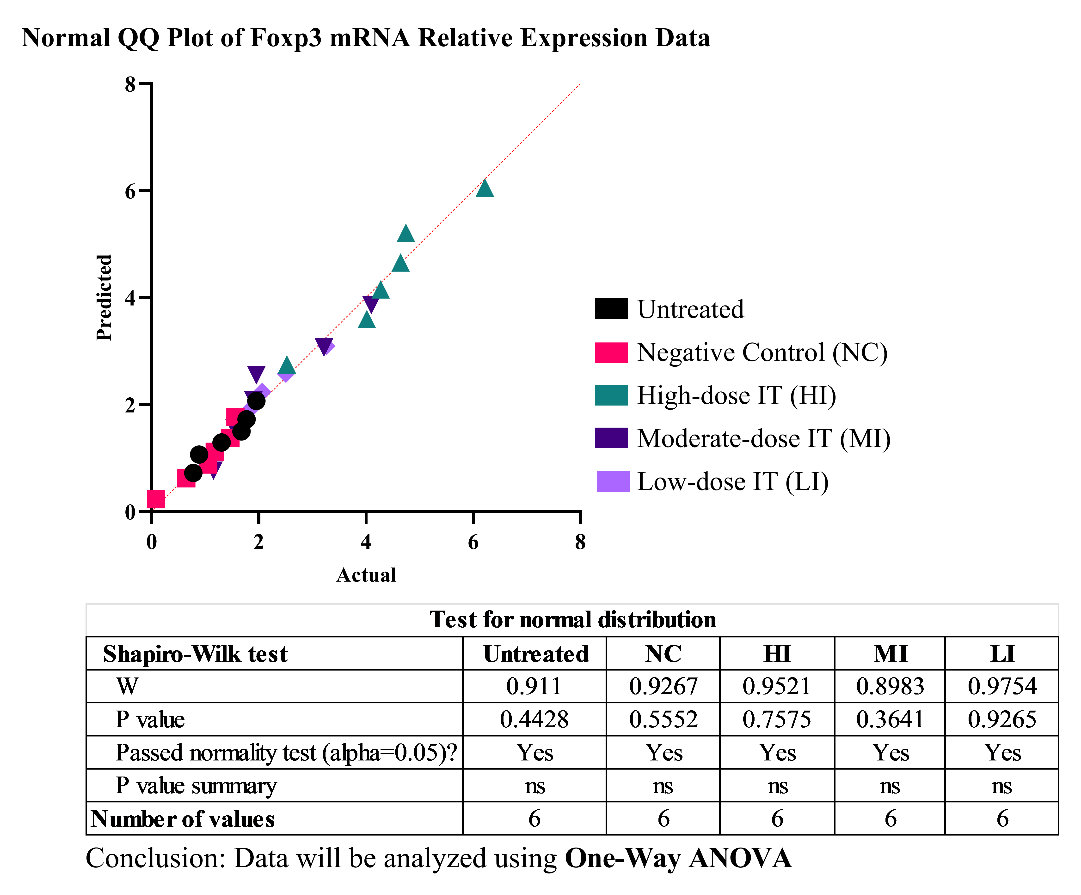
**

***Validation Results of Data 6:*
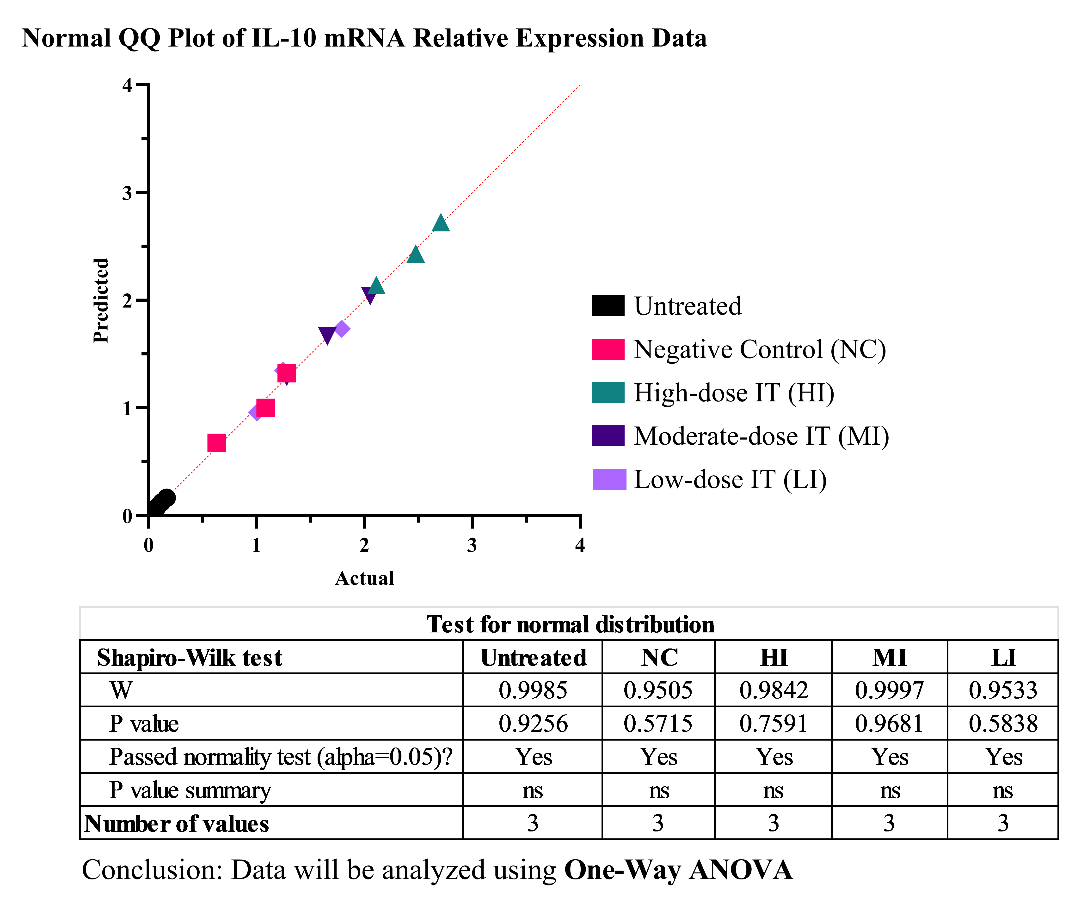
**

***Validation Results of Data 7:*
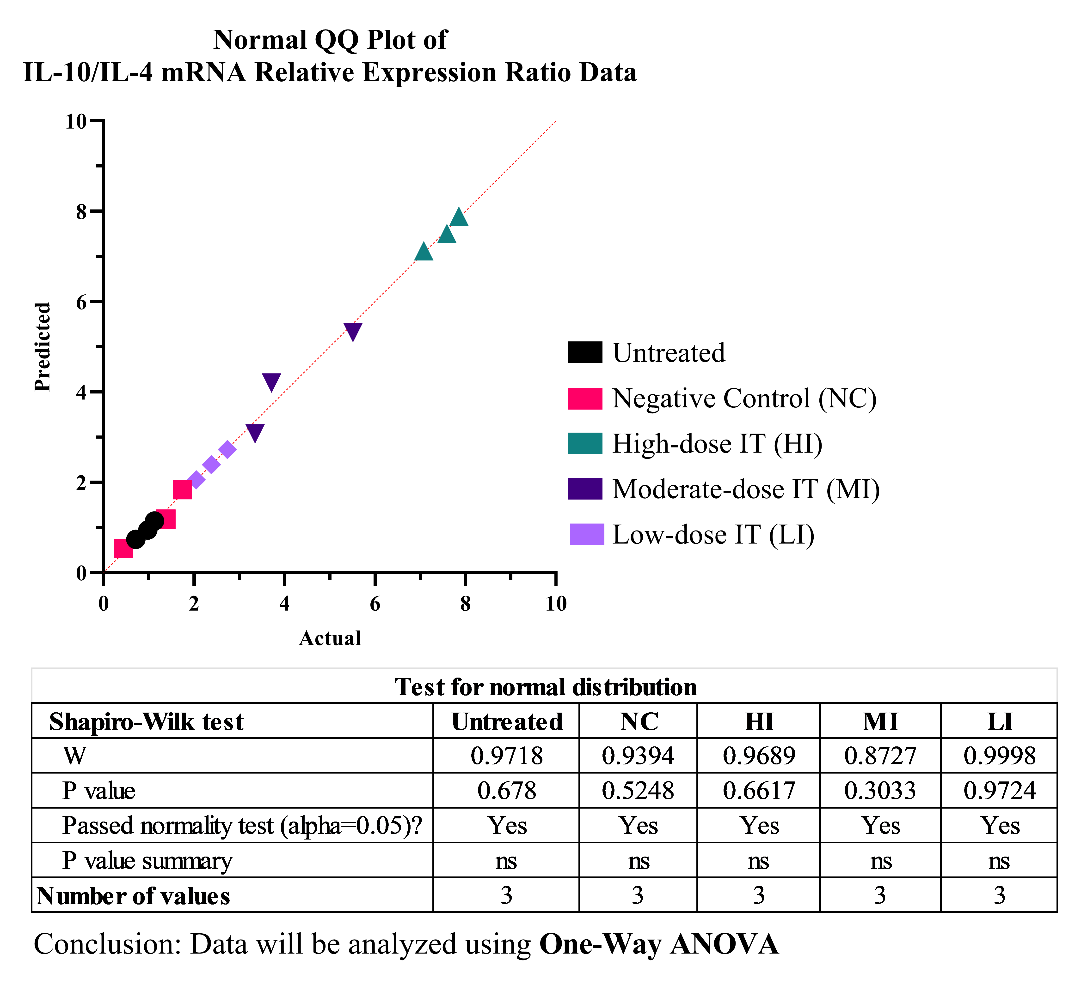
**

**
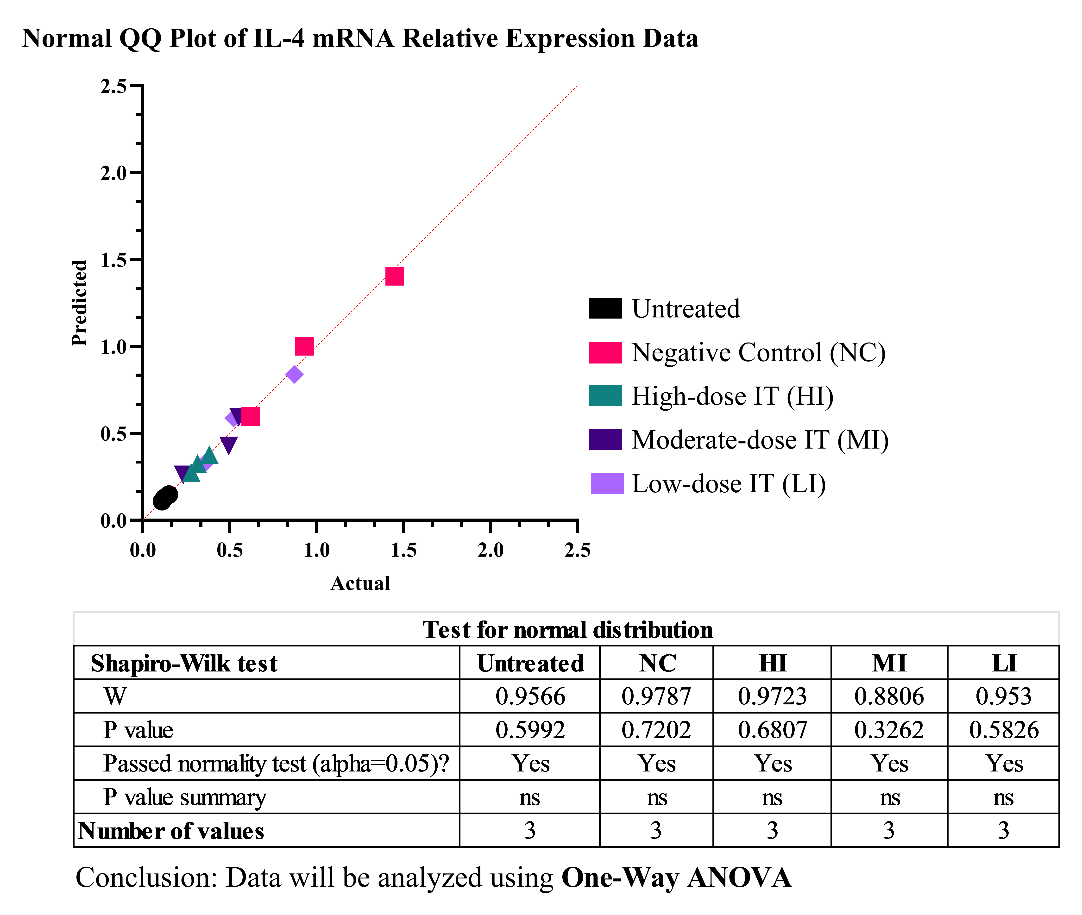
**

**References:**

1. Kuraishi Y, Nagasawa T, Hayashi K, Satoh M. Scratching behavior induced by pruritogenic but not algesiogenic agents in mice. *Eur J Pharmacol*. 1995;275(3):229-233.
2. Shimada SG, LaMotte RH. Behavioral differentiation between itch and pain in mouse. *Pain*. 2008;139(3):681-687.
3. Musoh K, Nakamura N, Sakurai T, Inagaki N, Nagai H. Scratching behavior in mice associated with IgE-mediated allergic cutaneous reaction and its pharmacological characterization. *Allergol Int*. 1997;46(2):177-124.
4. Boverhof DR, Billington R, Gollapudi BB, et al. Respiratory sensitization and allergy: current research approaches and needs. *Toxicol Appl Pharmacol*. 2008;226(1):1-13.
5. Navarro KL, Huss M, Smith JC, Sharp P, Marx JO, Pacharinsak C. Mouse Anesthesia: The Art and Science. *ILAR J*. 2021;62(1-2):238-273.
6. Castan L, Bøgh KL, Maryniak NZ, et al. Overview of in vivo and ex vivo endpoints in murine food allergy models: Suitable for evaluation of the sensitizing capacity of novel proteins?. *Allergy*. 2020;75(2):289-301.
7. Sivula CP, Suckow MA. Euthanasia. In: Weichbrod RH, Thompson GAH, Norton JN, editors. *Management of Animal Care and Use Programs in Research, Education, and Testing*. 2nd edition. Boca Raton (FL): CRC Press/Taylor & Francis; 2018. Chapter 35. Available from: <https://www.ncbi.nlm.nih.gov/books/NBK500441/>. Accessed on August 17, 2024.
8. Parasuraman S, Raveendran R, Kesavan R. Blood sample collection in small laboratory animals. *J Pharmacol Pharmacother*. 2017;8(3):153.
