# Supplementary material for: Shrimp allergen extract immunotherapy induces prolonged immune tolerance in a gastro-food allergy mouse model
Source: PLoS One. 2024 Dec 27;19(12):e0315312. doi: 10.1371/journal.pone.0315312 (PMC11676511; doi:10.1371/journal.pone.0315312)
Supplement: S1 Dataset — (DOCX) [file pone.0315312.s003.docx]

***S1 Dataset of SAE Proteomic Analysis***

**Shrimp allergen extract immunotherapy induces prolonged immune tolerance in a gastro-food allergy mouse model**

Honey Dzikri Marhaeny^1^, Lutfiatur Rohmah^1^, Yusuf Alif Pratama^1^, Salsabilla Madudari Kasatu^1^, Andang Miatmoko^2^, Rafi Addimaysqi^3^, Geert van den Bogaart^4^, Franz Y. Ho^5^, Muhammad Taher^6^, Junaidi Khotib^1*^

^1^Department of Pharmacy Practice, Faculty of Pharmacy, Airlangga University, Surabaya, Indonesia

^2^Department of Pharmaceutical Science, Faculty of Pharmacy, Airlangga University, Surabaya, Indonesia

^3^Faculty of Medicine, Airlangga University, Surabaya, Indonesia

^4^Department of Molecular Immunology and Microbiology, Groningen Biomolecular Sciences and Biotechnology Institute, Faculty of Science Engineering, University of Groningen, Groningen, The Netherlands

^5^GBB Proteomics, Groningen Biomolecular Sciences and Biotechnology Institute, Faculty of Science Engineering, University of Groningen, Groningen, The Netherlands

^6^Department of Pharmaceutical Technology, Kulliyyah of Pharmacy, International Islamic University Malaysia, Kuantan, Pahang, Malaysia

***Corresponding Author:**

Email: [junaidi-k@ff.unair.ac.id](mailto:junaidi-k@ff.unair.ac.id) (JK)

**S1 Dataset.** List of SAE proteins identified using a proteomic-based approach

| No. | Identified Protein | Accession | Average Mass (Da) | -10LgP | Peptides | Unique Protein | Post Translational Modification |
| --- | --- | --- | --- | --- | --- | --- | --- |
| 1 | Projectin | A0A423T5F8\|A0A423T5F8_PENVA | 621382.06 | 790.7694 | 374 | 374 | Acetylation (N-term); Carbamidomethylation; Deamidation (NQ); Oxidation (M) |
| 2 | Uncharacterized protein | A0A3R7PUB4\|A0A3R7PUB4_PENVA | 42421.793 | 81.71105 | 1 | 1 |  |
| 3 | Putative sodium-and chloride-dependent glycine transporter 1 | A0A3R7PKG8\|A0A3R7PKG8_PENVA | 68572.09 | 81.71105 | 1 | 1 |  |
| 4 | Uncharacterized protein | A0A3R7PEW9\|A0A3R7PEW9_PENVA | 47119.9 | 81.71105 | 1 | 1 |  |
| 5 | Ultraviolet-B receptor UVR8 | A0A423TMH5\|A0A423TMH5_PENVA | 197144.4 | 81.71105 | 1 | 1 |  |
| 6 | Putative sodium-and chloride-dependent glycine transporter 1-like isoform X3 | A0A3R7Q609\|A0A3R7Q609_PENVA | 49708.203 | 81.71105 | 1 | 1 |  |
| 7 | Putative insulin-like growth factor 1 receptor | A0A423SU77\|A0A423SU77_PENVA | 38738.17 | 81.71105 | 1 | 1 |  |
| 8 | Uncharacterized protein | A0A3R7PUG4\|A0A3R7PUG4_PENVA | 50358.844 | 81.71105 | 1 | 1 |  |
| 9 | Putative plexin-A2 | A0A3R7M1H6\|A0A3R7M1H6_PENVA | 162115.06 | 81.71105 | 1 | 1 |  |
| 10 | Uncharacterized protein | A0A423SWJ2\|A0A423SWJ2_PENVA | 43141.51 | 81.71105 | 1 | 1 |  |
| 11 | General transcription factor II-I repeat domain-containing protein 2-like | A0A423T470\|A0A423T470_PENVA | 33442.438 | 81.71105 | 1 | 1 |  |
| 12 | Uncharacterized protein | A0A423SBI9\|A0A423SBI9_PENVA | 91620.45 | 81.71105 | 1 | 1 |  |
| 13 | Uncharacterized protein | A0A3R7M9R9\|A0A3R7M9R9_PENVA | 46298.816 | 81.71105 | 1 | 1 |  |
| 14 | Glycosyl hydrolase family 63 C-terminal domain-containing protein | A0A3R7QMM8\|A0A3R7QMM8_PENVA | 100343.09 | 81.71105 | 1 | 1 |  |
| 15 | Uncharacterized protein | A0A423TYW1\|A0A423TYW1_PENVA | 45840.336 | 81.71105 | 1 | 1 |  |
| 16 | non-specific serine/threonine protein kinase | A0A423SI49\|A0A423SI49_PENVA | 164608.5 | 81.71105 | 1 | 1 |  |
| 17 | Uncharacterized protein | A0A3R7MS77\|A0A3R7MS77_PENVA | 49192.254 | 81.71105 | 1 | 1 |  |
| 18 | Putative transient receptor potential cation channel subfamily A member 1-like isoform X2 | A0A423SVL2\|A0A423SVL2_PENVA | 126315.8 | 81.71105 | 1 | 1 |  |
| 19 | Myosin heavy chain type 2 | A0A423SVE3\|A0A423SVE3_PENVA | 86903.26 | 747.9656 | 296 | 7 | Acetylation (N-term); Carbamidomethylation; Deamidation (NQ); Oxidation (M) |
| 20 | I-connectin | A0A3R7NSY1\|A0A3R7NSY1_PENVA | 314709.9 | 747.4676 | 275 | 231 | Carbamidomethylation; Deamidation (NQ); Oxidation (M) |
| 21 | I-connectin | A0A3R7MHR0\|A0A3R7MHR0_PENVA | 152602.38 | 646.9273 | 123 | 122 | Carbamidomethylation; Deamidation (NQ); Oxidation (M) |
| 22 | I-connectin | A0A3R7NAT2\|A0A3R7NAT2_PENVA | 66263.61 | 491.99564 | 36 | 36 | Carbamidomethylation; Deamidation (NQ); Oxidation (M) |
| 23 | Kettin | A0A3R7SYQ8\|A0A3R7SYQ8_PENVA | 20898.5 | 409.59863 | 16 | 16 | Carbamidomethylation; Deamidation (NQ); Oxidation (M) |
| 24 | I-connectin | A0A423U036\|A0A423U036_PENVA | 22661.49 | 358.34988 | 12 | 12 | Carbamidomethylation; Oxidation (M) |
| 25 | Myosin heavy chain type 2 | A0A423SVB5\|A0A423SVB5_PENVA | 79024.4 | 739.25574 | 276 | 9 | Acetylation (N-term); Carbamidomethylation; Deamidation (NQ); Oxidation (M) |
| 26 | Myosin heavy chain type 2 | A0A423SVI4\|A0A423SVI4_PENVA | 30354.11 | 568.63324 | 73 | 9 | Acetylation (N-term); Carbamidomethylation; Deamidation (NQ); Oxidation (M) |
| 27 | Myosin heavy chain type 1 | A0A423SDG5\|A0A423SDG5_PENVA | 81163.58 | 734.43744 | 268 | 1 | Acetylation (N-term); Carbamidomethylation; Deamidation (NQ); Oxidation (M) |
| 28 | Paramyosin | A0A3R7QCP1\|A0A3R7QCP1_PENVA | 96537.445 | 723.6291 | 256 | 255 | Acetylation (N-term); Carbamidomethylation; Deamidation (NQ); Oxidation (M) |
| 29 | Putative adenylate kinase isoenzyme 5-like (Fragment) | A0A423SRM8\|A0A423SRM8_PENVA | 56003.234 | 133.1882 | 2 | 2 |  |
| 30 | Myosin heavy chain type 1 | A0A423SDH1\|A0A423SDH1_PENVA | 79567.85 | 713.79407 | 223 | 28 | Acetylation (N-term); Carbamidomethylation; Deamidation (NQ); Oxidation (M) |
| 31 | Myosin heavy chain type 3 | A0A423T859\|A0A423T859_PENVA | 32050.78 | 609.61383 | 95 | 16 | Acetylation (N-term); Deamidation (NQ); Oxidation (M) |
| 32 | Protein kinase domain-containing protein | A0A3R7QNZ5\|A0A3R7QNZ5_PENVA | 35560.184 | 61.42547 | 1 | 1 |  |
| 33 | Myosin heavy chain type 1 | A0A423T857\|A0A423T857_PENVA | 110727.85 | 706.4258 | 222 | 15 | Acetylation (N-term); Carbamidomethylation; Deamidation (NQ); Oxidation (M) |
| 34 | Myosin heavy chain type 1 | A0A423SDE1\|A0A423SDE1_PENVA | 59398.195 | 704.35126 | 204 | 10 | Acetylation (N-term); Carbamidomethylation; Deamidation (NQ); Oxidation (M) |
| 35 | Myosin heavy chain type 1 | A0A423T881\|A0A423T881_PENVA | 36020.277 | 581.67645 | 76 | 6 | Acetylation (N-term); Deamidation (NQ); Oxidation (M) |
| 36 | Myosin heavy chain type 3 | A0A423SDG2\|A0A423SDG2_PENVA | 23971.209 | 457.9402 | 28 | 3 | Acetylation (N-term); Deamidation (NQ); Oxidation (M) |
| 37 | Pyruvate kinase | A0A423U7J5\|A0A423U7J5_PENVA | 63343.15 | 698.4349 | 190 | 190 | Acetylation (N-term); Carbamidomethylation; Deamidation (NQ); Oxidation (M) |
| 38 | Pyruvate kinase (Fragment) | A0A3R7MJT9\|A0A3R7MJT9_PENVA | 60029.484 | 675.05865 | 158 | 158 | Acetylation (N-term); Carbamidomethylation; Deamidation (NQ); Oxidation (M) |
| 39 | Uncharacterized protein | A0A3R7PGX7\|A0A3R7PGX7_PENVA | 64014.54 | 82.59973 | 1 | 1 |  |
| 40 | Methyltransferase FkbM domain-containing protein | A0A423T802\|A0A423T802_PENVA | 50375.535 | 82.59973 | 1 | 1 |  |
| 41 | Putative glutamic acid-rich protein-like | A0A423SL25\|A0A423SL25_PENVA | 126156.07 | 58.50381 | 1 | 1 |  |
| 42 | tRNA (adenine(58)-N(1))-methyltransferase | A0A3R7Q978\|A0A3R7Q978_PENVA | 159377.23 | 36.244896 | 1 | 1 |  |
| 43 | oligopeptidase A | A0A423SGL9\|A0A423SGL9_PENVA | 83541.97 | 36.244896 | 1 | 1 |  |
| 44 | Putative Neuroglian | A0A423SSF4\|A0A423SSF4_PENVA | 214640.97 | 692.26886 | 166 | 166 | Acetylation (N-term); Carbamidomethylation; Deamidation (NQ); Oxidation (M) |
| 45 | I-connectin | A0A423SNX9\|A0A423SNX9_PENVA | 477660.22 | 688.3847 | 168 | 55 | Acetylation (N-term); Carbamidomethylation; Deamidation (NQ); Oxidation (M) |
| 46 | I-connectin | A0A423U038\|A0A423U038_PENVA | 151897.5 | 337.80374 | 10 | 10 | Carbamidomethylation; Oxidation (M) |
| 47 | I-connectin | A0A423U050\|A0A423U050_PENVA | 15848.764 | 244.12724 | 4 | 4 | Carbamidomethylation |
| 48 | Myosin heavy chain type a | A0A423TZR1\|A0A423TZR1_PENVA | 77680.78 | 679.5818 | 169 | 1 | Acetylation (N-term); Carbamidomethylation; Deamidation (NQ); Oxidation (M) |
| 49 | Myosin heavy chain type a | A0A423SWT0\|A0A423SWT0_PENVA | 77703.87 | 676.39294 | 163 | 3 | Acetylation (N-term); Deamidation (NQ); Oxidation (M) |
| 50 | Projectin | A0A423T5T3\|A0A423T5T3_PENVA | 197685.52 | 676.2954 | 152 | 152 | Acetylation (N-term); Carbamidomethylation; Deamidation (NQ); Oxidation (M) |
| 51 | Neurabin-1 | A0A3R7QCF4\|A0A3R7QCF4_PENVA | 157609.17 | 34.41568 | 1 | 1 |  |
| 52 | Myosin heavy chain type 1 | A0A3R7SLR1\|A0A3R7SLR1_PENVA | 81021.56 | 668.22534 | 154 | 3 | Acetylation (N-term); Deamidation (NQ); Oxidation (M) |
| 53 | I-connectin | A0A3R7QKU8\|A0A3R7QKU8_PENVA | 248937.61 | 667.7864 | 141 | 2 | Acetylation (N-term); Carbamidomethylation; Deamidation (NQ); Oxidation (M) |
| 54 | Hemocyanin | X2KWE4\|X2KWE4_PENVA | 74989.89 | 663.84503 | 140 | 118 | Acetylation (N-term); Carbamidomethylation; Deamidation (NQ); Oxidation (M) |
| 55 | Hemocyanin | A0A3R7SZZ8\|A0A3R7SZZ8_PENVA | 73484.43 | 647.86145 | 124 | 102 | Acetylation (N-term); Carbamidomethylation; Deamidation (NQ); Oxidation (M) |
| 56 | Hemocyanin | A0A3R7PUZ2\|A0A3R7PUZ2_PENVA | 29515.643 | 575.61993 | 68 | 58 | Deamidation (NQ) |
| 57 | Myosin heavy chain type a | A0A423U4K8\|A0A423U4K8_PENVA | 87641.94 | 662.0441 | 147 | 1 | Acetylation (N-term); Carbamidomethylation; Deamidation (NQ); Oxidation (M) |
| 58 | Myosin heavy chain type 1 | A0A423TG84\|A0A423TG84_PENVA | 75595.41 | 658.13477 | 142 | 6 | Acetylation (N-term); Carbamidomethylation; Deamidation (NQ); Oxidation (M) |
| 59 | Myosin heavy chain, isoform N | A0A423TZS3\|A0A423TZS3_PENVA | 39845.1 | 508.49887 | 45 | 2 | Acetylation (N-term); Carbamidomethylation; Deamidation (NQ); Oxidation (M) |
| 60 | Isoform of A0A423TG84, Myosin heavy chain type 5 | A0A423TZN2\|A0A423TZN2_PENVA | 19331.793 | 427.19464 | 21 | 1 | Acetylation (N-term); Deamidation (NQ); Oxidation (M) |
| 61 | Myosin heavy chain type 2 (Fragment) | A0A3R7PJ50\|A0A3R7PJ50_PENVA | 77180.29 | 656.53345 | 140 | 11 | Acetylation (N-term); Deamidation (NQ); Oxidation (M) |
| 62 | Myosin heavy chain type b | A0A3R7Q491\|A0A3R7Q491_PENVA | 78063.23 | 655.763 | 151 | 1 | Acetylation (N-term); Carbamidomethylation; Deamidation (NQ); Oxidation (M) |
| 63 | Slow muscle myosin S1 heavy chain | A0A3R7M4R5\|A0A3R7M4R5_PENVA | 61842.418 | 654.4095 | 136 | 7 | Acetylation (N-term); Deamidation (NQ); Oxidation (M) |
| 64 | Myosin heavy chain type 2 | A0A3R7MR99\|A0A3R7MR99_PENVA | 74242.23 | 653.3239 | 142 | 4 | Acetylation (N-term); Carbamidomethylation; Deamidation (NQ); Oxidation (M) |
| 65 | Myosin heavy chain type b | A0A3R7QC09\|A0A3R7QC09_PENVA | 35931.848 | 432.26343 | 24 | 1 | Acetylation (N-term); Carbamidomethylation; Deamidation (NQ) |
| 66 | Calcium-transporting ATPase | A0A423TIS4\|A0A423TIS4_PENVA | 101820.05 | 644.08966 | 118 | 87 | Acetylation (N-term); Carbamidomethylation; Deamidation (NQ); Oxidation (M) |
| 67 | Sarco/endoplasmic reticulum Ca2+-ATPase | A0A3R7QEN1\|A0A3R7QEN1_PENVA | 46702.49 | 545.9614 | 51 | 23 | Acetylation (N-term); Carbamidomethylation; Deamidation (NQ); Oxidation (M) |
| 68 | Isoform of A0A3R7QFA6, Sarcoplasmic/endoplasmic reticulum calcium ATPase 1-like (Fragment) | A0A423TKM2\|A0A423TKM2_PENVA | 15834.465 | 243.39507 | 5 | 5 | Deamidation (NQ); Oxidation (M) |
| 69 | Uncharacterized protein | A0A423SSB1\|A0A423SSB1_PENVA | 105772.44 | 51.38197 | 1 | 1 | Carbamidomethylation |
| 70 | Sarcoplasmic calcium-binding protein | A0A3R7SPI4\|A0A3R7SPI4_PENVA | 31372.2 | 644.0126 | 119 | 69 | Acetylation (N-term); Carbamidomethylation; Deamidation (NQ); Oxidation (M) |
| 71 | Myosin heavy chain type 1 | A0A423TG50\|A0A423TG50_PENVA | 77714.734 | 642.6262 | 125 | 5 | Acetylation (N-term); Deamidation (NQ); Oxidation (M) |
| 72 | Myosin heavy chain type a | A0A3R7PCK5\|A0A3R7PCK5_PENVA | 54628.69 | 607.3667 | 94 | 3 | Acetylation (N-term); Deamidation (NQ); Oxidation (M) |
| 73 | Isoform of A0A423TG50, Myosin heavy chain type 5 | A0A3R7MNQ0\|A0A3R7MNQ0_PENVA | 21095.771 | 425.39563 | 21 | 1 | Acetylation (N-term); Deamidation (NQ); Oxidation (M) |
| 74 | Myosin heavy chain type 1 | A0A3R7MGC9\|A0A3R7MGC9_PENVA | 80922.54 | 641.33276 | 123 | 11 | Acetylation (N-term); Carbamidomethylation; Deamidation (NQ); Oxidation (M) |
| 75 | Actin 2 | A0A423SYH5\|A0A423SYH5_PENVA | 43863.06 | 636.5561 | 119 | 1 | Acetylation (N-term); Carbamidomethylation; Deamidation (NQ); Oxidation (M) |
| 76 | Glyceraldehyde-3-phosphate dehydrogenase | A0A3R7PW84\|A0A3R7PW84_PENVA | 49928.19 | 633.17346 | 114 | 114 | Acetylation (N-term); Carbamidomethylation; Deamidation (NQ); Oxidation (M) |
| 77 | Glyceraldehyde-3-phosphate dehydrogenase | A0A3R7QF47\|A0A3R7QF47_PENVA | 26821.074 | 553.94214 | 62 | 62 | Carbamidomethylation; Deamidation (NQ); Oxidation (M) |
| 78 | Alpha-(1,6)-fucosyltransferase | A0A3R7Q0N4\|A0A3R7Q0N4_PENVA | 40161.695 | 46.588093 | 1 | 1 |  |
| 79 | Hemocyanin | A0A3R7Q123\|A0A3R7Q123_PENVA | 77359.26 | 631.01434 | 111 | 17 | Acetylation (N-term); Carbamidomethylation; Deamidation (NQ); Oxidation (M) |
| 80 | Myosin heavy chain type 2 | A0A3R7PJ45\|A0A3R7PJ45_PENVA | 48382.883 | 630.92255 | 123 | 2 | Acetylation (N-term); Deamidation (NQ); Oxidation (M) |
| 81 | Hemocyanin subunit L2 | A0A423SGU8\|A0A423SGU8_PENVA | 152108.44 | 627.4268 | 105 | 3 | Carbamidomethylation; Deamidation (NQ); Oxidation (M) |
| 82 | Hemocyanin subunit L2 | A0A3R7LZS3\|A0A3R7LZS3_PENVA | 73933.47 | 601.10065 | 86 | 3 | Carbamidomethylation; Deamidation (NQ); Oxidation (M) |
| 83 | Sarcoplasmic calcium-binding protein, beta chain | A0A423T8H7\|A0A423T8H7_PENVA | 21924.568 | 626.40466 | 101 | 49 | Acetylation (N-term); Carbamidomethylation; Deamidation (NQ); Oxidation (M) |
| 84 | Muscle LIM protein Mlp84B | A0A423TH89\|A0A423TH89_PENVA | 96907.15 | 624.4274 | 93 | 93 | Acetylation (N-term); Carbamidomethylation; Deamidation (NQ); Oxidation (M) |
| 85 | Putative nesprin-1 | A0A3R7M8Q6\|A0A3R7M8Q6_PENVA | 55456.156 | 621.0694 | 108 | 107 | Acetylation (N-term); Carbamidomethylation; Deamidation (NQ); Oxidation (M) |
| 86 | Muscle M-line assembly protein unc-89 | A0A423SR14\|A0A423SR14_PENVA | 418029.22 | 617.0954 | 97 | 97 | Acetylation (N-term); Carbamidomethylation; Deamidation (NQ); Oxidation (M) |
| 87 | Muscle M-line assembly protein unc-89 | A0A3R7PZK0\|A0A3R7PZK0_PENVA | 46384.527 | 337.9401 | 10 | 10 | Carbamidomethylation; Deamidation (NQ) |
| 88 | Guanine nucleotide exchange factor VAV2 | A0A423U0B6\|A0A423U0B6_PENVA | 90676.41 | 264.40234 | 5 | 5 |  |
| 89 | Putative muscle M-line assembly protein unc-89-like isoform X1 | A0A423U0A5\|A0A423U0A5_PENVA | 14814.743 | 220.66579 | 4 | 4 | Carbamidomethylation; Deamidation (NQ) |
| 90 | Myosin heavy chain type 2 | A0A3R7NVY6\|A0A3R7NVY6_PENVA | 48672.26 | 615.8783 | 108 | 1 | Acetylation (N-term); Deamidation (NQ); Oxidation (M) |
| 91 | Beta-actin | A0A3R7Q1H5\|A0A3R7Q1H5_PENVA | 40352.156 | 615.69653 | 102 | 2 | Acetylation (N-term); Carbamidomethylation; Deamidation (NQ); Oxidation (M) |
| 92 | Neuroblast differentiation-associated protein AHNAK | A0A423T9X4\|A0A423T9X4_PENVA | 158017.23 | 610.7026 | 90 | 16 | Deamidation (NQ); Oxidation (M) |
| 93 | Myosin heavy chain type a | A0A3R7SNQ8\|A0A3R7SNQ8_PENVA | 76159.2 | 609.15875 | 98 | 1 | Acetylation (N-term); Carbamidomethylation; Deamidation (NQ); Oxidation (M) |
| 94 | Actin 2 | A0A3R7M7H4\|A0A3R7M7H4_PENVA | 41939.96 | 608.6054 | 98 | 3 | Carbamidomethylation; Deamidation (NQ); Oxidation (M) |
| 95 | Actin 2 | A0A3R7M3B1\|A0A3R7M3B1_PENVA | 41878.844 | 608.1393 | 97 | 2 | Acetylation (N-term); Carbamidomethylation; Deamidation (NQ); Oxidation (M) |
| 96 | Myosin heavy chain type 2 | A0A3R7QXH4\|A0A3R7QXH4_PENVA | 72638.61 | 606.3549 | 93 | 2 | Acetylation (N-term); Deamidation (NQ); Oxidation (M) |
| 97 | Actin 2 | A0A3R7PK58\|A0A3R7PK58_PENVA | 40223.094 | 603.3724 | 93 | 1 | Acetylation (N-term); Carbamidomethylation; Deamidation (NQ); Oxidation (M) |
| 98 | Beta-1,3-glucan binding protein | A0A423T1V1\|A0A423T1V1_PENVA | 216534.39 | 603.09393 | 91 | 28 | Deamidation (NQ); Oxidation (M) |
| 99 | Myosin heavy chain type 1 | A0A423U4K1\|A0A423U4K1_PENVA | 52556.246 | 602.6937 | 95 | 2 | Acetylation (N-term); Carbamidomethylation; Deamidation (NQ); Oxidation (M) |
| 100 | Actin 2 | A0A423TES4\|A0A423TES4_PENVA | 41611.727 | 600.76874 | 92 | 12 | Acetylation (N-term); Carbamidomethylation; Deamidation (NQ); Oxidation (M) |
| 101 | Putative ryanodine receptor 44F isoform X4 | A0A423U9Z0\|A0A423U9Z0_PENVA | 269654.34 | 599.4528 | 83 | 82 | Carbamidomethylation; Deamidation (NQ); Oxidation (M) |
| 102 | arginine kinase | A0A3R7PEF6\|A0A3R7PEF6_PENVA | 40131.793 | 596.93115 | 90 | 90 | Acetylation (N-term); Carbamidomethylation; Deamidation (NQ); Oxidation (M) |
| 103 | >sp\|KCRM_HUMAN\| | #CONTAM#KCRM_HUMAN\| | 43101.113 | 210.09093 | 3 | 3 | Deamidation (NQ) |
| 104 | Putative serine/threonine-protein phosphatase 6 regulatory ankyrin repeat subunit B-like | A0A3R7SY54\|A0A3R7SY54_PENVA | 155094.86 | 40.434322 | 1 | 1 |  |
| 105 | I-connectin | A0A423TF99\|A0A423TF99_PENVA | 287227.28 | 594.5668 | 79 | 63 | Acetylation (N-term); Carbamidomethylation; Deamidation (NQ); Oxidation (M) |
| 106 | Neuroblast differentiation-associated protein AHNAK | A0A423T9R6\|A0A423T9R6_PENVA | 76030.15 | 589.8275 | 74 | 5 | Oxidation (M) |
| 107 | Myosin heavy chain type 2 | A0A423T8T8\|A0A423T8T8_PENVA | 66339.38 | 589.6815 | 85 | 12 | Acetylation (N-term); Carbamidomethylation; Deamidation (NQ); Oxidation (M) |
| 108 | Fructose-bisphosphate aldolase | A0A3R7PR20\|A0A3R7PR20_PENVA | 52885.316 | 587.6275 | 77 | 60 | Acetylation (N-term); Carbamidomethylation; Deamidation (NQ); Oxidation (M) |
| 109 | Actin T2 | A0A3R7QFV3\|A0A3R7QFV3_PENVA | 41766.727 | 587.30286 | 82 | 1 | Carbamidomethylation; Deamidation (NQ); Oxidation (M) |
| 110 | Actin T2 | A0A423SPF0\|A0A423SPF0_PENVA | 40939.92 | 587.0794 | 82 | 1 | Acetylation (N-term); Carbamidomethylation; Deamidation (NQ); Oxidation (M) |
| 111 | Troponin I (Fragment) | A0A3R7MLK9\|A0A3R7MLK9_PENVA | 42791.004 | 586.41235 | 75 | 74 | Deamidation (NQ); Oxidation (M) |
| 112 | Triosephosphate isomerase | K0E682\|K0E682_PENVA | 26994.918 | 583.4357 | 75 | 75 | Acetylation (N-term); Carbamidomethylation; Deamidation (NQ); Oxidation (M) |
| 113 | Beta-1,3-glucan-binding protein | A0A3R7NYM7\|A0A3R7NYM7_PENVA | 116077.37 | 578.9274 | 74 | 10 | Deamidation (NQ); Oxidation (M) |
| 114 | Actin T2 | A0A3R7LW53\|A0A3R7LW53_PENVA | 38372.703 | 578.48975 | 75 | 1 | Carbamidomethylation; Deamidation (NQ); Oxidation (M) |
| 115 | Sarcoplasmic calcium-binding protein variant a | A0A3R7NZC3\|A0A3R7NZC3_PENVA | 33075.246 | 577.85944 | 72 | 43 | Acetylation (N-term); Carbamidomethylation; Deamidation (NQ); Oxidation (M) |
| 116 | Putative filamin-A isoform X4 | A0A3R7Q393\|A0A3R7Q393_PENVA | 99699.15 | 577.51306 | 71 | 71 | Carbamidomethylation; Deamidation (NQ); Oxidation (M) |
| 117 | Alpha-1,4 glucan phosphorylase | A0A423U2L6\|A0A423U2L6_PENVA | 97836.9 | 576.8017 | 69 | 69 | Acetylation (N-term); Carbamidomethylation; Deamidation (NQ); Oxidation (M) |
| 118 | Toll3 | A0A3R7MEZ1\|A0A3R7MEZ1_PENVA | 122217.19 | 59.748726 | 1 | 1 |  |
| 119 | Calphotin (Fragment) | A0A423U2N3\|A0A423U2N3_PENVA | 71592.43 | 571.556 | 68 | 68 | Acetylation (N-term); Deamidation (NQ); Oxidation (M) |
| 120 | Uncharacterized protein | A0A3R7MVP3\|A0A3R7MVP3_PENVA | 86039.44 | 30.94561 | 1 | 1 |  |
| 121 | Ras-associating domain-containing protein | A0A423T8Z4\|A0A423T8Z4_PENVA | 99670.79 | 30.94561 | 1 | 1 |  |
| 122 | G-protein coupled receptors family 1 profile domain-containing protein | A0A3R7NWQ5\|A0A3R7NWQ5_PENVA | 103689.336 | 30.94561 | 1 | 1 |  |
| 123 | Uncharacterized protein | A0A3R7SMQ0\|A0A3R7SMQ0_PENVA | 59063.773 | 30.94561 | 1 | 1 |  |
| 124 | Putative chromosome-associated kinesin KIF4-like | A0A3R7PZB5\|A0A3R7PZB5_PENVA | 94078.81 | 30.94561 | 1 | 1 |  |
| 125 | Uncharacterized protein | A0A423TRE2\|A0A423TRE2_PENVA | 31589.117 | 27.520475 | 1 | 1 |  |
| 126 | Uncharacterized protein | A0A423SIL5\|A0A423SIL5_PENVA | 216915.78 | 27.520475 | 1 | 1 |  |
| 127 | Basic proline-rich protein-like | A0A3R7QMF7\|A0A3R7QMF7_PENVA | 94742.4 | 27.520475 | 1 | 1 |  |
| 128 | Uncharacterized protein | A0A3R7N3G3\|A0A3R7N3G3_PENVA | 46839.434 | 27.520475 | 1 | 1 |  |
| 129 | Katanin p60 ATPase-containing subunit A-like 1 | A0A423TKQ7\|A0A423TKQ7_PENVA | 44786.637 | 27.520475 | 1 | 1 |  |
| 130 | Chorion peroxidase | A0A3R7PVP8\|A0A3R7PVP8_PENVA | 95710.37 | 27.520475 | 1 | 1 |  |
| 131 | Latrophilin Cirl | A0A3R7QH93\|A0A3R7QH93_PENVA | 149940.53 | 27.520475 | 1 | 1 |  |
| 132 | Putative adhesive plaque matrix protein-like | A0A423TR94\|A0A423TR94_PENVA | 69142.48 | 27.520475 | 1 | 1 |  |
| 133 | Putative long-chain-fatty-acid--CoA ligase 4 isoform X3 | A0A3R7MYA9\|A0A3R7MYA9_PENVA | 37145.676 | 27.520475 | 1 | 1 |  |
| 134 | Uncharacterized protein | A0A3R7PWN3\|A0A3R7PWN3_PENVA | 105220.03 | 27.520475 | 1 | 1 |  |
| 135 | DUF676 domain-containing protein | A0A423SH23\|A0A423SH23_PENVA | 181153.2 | 27.520475 | 1 | 1 |  |
| 136 | Hemocyanin | A0A3R7PEN2\|A0A3R7PEN2_PENVA | 64229.773 | 569.88275 | 69 | 4 | Acetylation (N-term); Carbamidomethylation; Deamidation (NQ); Oxidation (M) |
| 137 | Myosin heavy chain type a | A0A3R7MHI7\|A0A3R7MHI7_PENVA | 65846.39 | 569.536 | 73 | 2 | Acetylation (N-term); Carbamidomethylation; Deamidation (NQ); Oxidation (M) |
| 138 | Hemocyanin subunit L1 | A0A3R7LSN5\|A0A3R7LSN5_PENVA | 52553.473 | 567.87994 | 65 | 28 | Acetylation (N-term); Carbamidomethylation; Deamidation (NQ); Oxidation (M) |
| 139 | Actin T2 | A0A3R7M0K4\|A0A3R7M0K4_PENVA | 41861.875 | 567.57806 | 71 | 3 | Acetylation (N-term); Carbamidomethylation; Deamidation (NQ); Oxidation (M) |
| 140 | Myosin heavy chain type 2 | A0A3R7QD31\|A0A3R7QD31_PENVA | 80088.78 | 565.73285 | 80 | 12 | Acetylation (N-term); Carbamidomethylation; Deamidation (NQ); Oxidation (M) |
| 141 | Skeletal muscle actin 6 | A0A3R7PEZ5\|A0A3R7PEZ5_PENVA | 41783.812 | 563.25714 | 68 | 1 | Carbamidomethylation; Deamidation (NQ); Oxidation (M) |
| 142 | Actin 1 | A0A3R7LYA4\|A0A3R7LYA4_PENVA | 41863.035 | 558.034 | 65 | 1 | Acetylation (N-term); Carbamidomethylation; Deamidation (NQ); Oxidation (M) |
| 143 | Myosin heavy chain type 2 | A0A3R7NAL8\|A0A3R7NAL8_PENVA | 67059.91 | 557.34827 | 80 | 15 | Acetylation (N-term); Deamidation (NQ); Oxidation (M) |
| 144 | Myosin heavy chain type 2 | A0A423U0L3\|A0A423U0L3_PENVA | 39851.56 | 504.88348 | 55 | 14 | Acetylation (N-term); Deamidation (NQ); Oxidation (M) |
| 145 | Myosin heavy chain type 6b | A0A423U0M2\|A0A423U0M2_PENVA | 26093.062 | 384.80594 | 18 | 1 | Deamidation (NQ) |
| 146 | Uncharacterized protein | A0A423TYG8\|A0A423TYG8_PENVA | 176455.38 | 38.061207 | 2 | 2 |  |
| 147 | Uncharacterized protein | A0A3R7QK35\|A0A3R7QK35_PENVA | 219627.38 | 38.061207 | 2 | 2 |  |
| 148 | Uncharacterized protein | A0A423TY80\|A0A423TY80_PENVA | 186831.97 | 38.061207 | 2 | 2 |  |
| 149 | Putative DNA polymerase eta | A0A423THW9\|A0A423THW9_PENVA | 27826.559 | 27.438093 | 1 | 1 |  |
| 150 | U4/U6 small nuclear ribonucleoprotein Prp31 | A0A3R7PS26\|A0A3R7PS26_PENVA | 55328.387 | 27.438093 | 1 | 1 |  |
| 151 | Slow tropomyosin isoform | A0A423SL41\|A0A423SL41_PENVA | 17765.904 | 557.1086 | 61 | 61 | Acetylation (N-term); Deamidation (NQ); Oxidation (M) |
| 152 | SWI/SNF-related matrix-associated actin-dependent regulator of chromatin subfamily A-like protein 1 | A0A3R7N0G7\|A0A3R7N0G7_PENVA | 54154.16 | 27.376368 | 1 | 1 |  |
| 153 | >sp\|TRYP_PIG\| | #CONTAM#TRYP_PIG\| | 24409.47 | 555.52747 | 59 | 58 | Acetylation (N-term); Carbamidomethylation; Deamidation (NQ); Oxidation (M) |
| 154 | Actin 1 | A0A3R7PEZ1\|A0A3R7PEZ1_PENVA | 41730.734 | 548.6307 | 62 | 1 | Acetylation (N-term); Carbamidomethylation; Deamidation (NQ); Oxidation (M) |
| 155 | Troponin H | A0A3R7PA33\|A0A3R7PA33_PENVA | 34693.082 | 545.3097 | 67 | 64 | Acetylation (N-term); Deamidation (NQ); Oxidation (M) |
| 156 | Uncharacterized protein | A0A423SCS0\|A0A423SCS0_PENVA | 97175.63 | 90.54002 | 2 | 1 |  |
| 157 | RRM domain-containing protein | A0A3R7PXM7\|A0A3R7PXM7_PENVA | 124404.53 | 85.149956 | 1 | 1 |  |
| 158 | isoleucine--tRNA ligase | A0A423TPH2\|A0A423TPH2_PENVA | 110976.12 | 85.149956 | 1 | 1 |  |
| 159 | Queuosine 5'-phosphate N-glycosylase/hydrolase | A0A423T9F2\|A0A423T9F2_PENVA | 41851.402 | 56.33575 | 1 | 1 |  |
| 160 | Phorbol-ester/DAG-type domain-containing protein | A0A423TAE0\|A0A423TAE0_PENVA | 76333.875 | 30.095707 | 1 | 1 |  |
| 161 | Troponin C1 (Fragment) | A0A3R7Q302\|A0A3R7Q302_PENVA | 15515.338 | 541.54736 | 50 | 32 | Acetylation (N-term); Carbamidomethylation; Deamidation (NQ); Oxidation (M) |
| 162 | Actin 1 | A0A2H4V3D5\|A0A2H4V3D5_PENVA | 41620.68 | 538.3929 | 56 | 1 | Acetylation (N-term); Carbamidomethylation; Deamidation (NQ); Oxidation (M) |
| 163 | Skeletal muscle actin 8 | A0A3R7M018\|A0A3R7M018_PENVA | 41777.87 | 534.73846 | 56 | 1 | Carbamidomethylation; Deamidation (NQ); Oxidation (M) |
| 164 | Actin 1 | A0A3R7NX50\|A0A3R7NX50_PENVA | 41715.82 | 534.1899 | 55 | 1 | Acetylation (N-term); Carbamidomethylation; Deamidation (NQ); Oxidation (M) |
| 165 | Putative alpha-actinin, sarcomeric isoform X2 | A0A423TJG5\|A0A423TJG5_PENVA | 125063.57 | 530.8274 | 50 | 49 | Carbamidomethylation; Deamidation (NQ); Oxidation (M) |
| 166 | Uncharacterized protein | A0A3R7QPW9\|A0A3R7QPW9_PENVA | 42660.62 | 62.396423 | 1 | 1 |  |
| 167 | Myosin light chain 2 | A0A3R7PTG0\|A0A3R7PTG0_PENVA | 18719.922 | 526.805 | 47 | 42 | Deamidation (NQ); Oxidation (M) |
| 168 | Myosin light chain 2 | A0A3R7LUL9\|A0A3R7LUL9_PENVA | 16577.416 | 271.09744 | 6 | 1 | Oxidation (M) |
| 169 | Myosin light chain 2 | A0A3R7SJC3\|A0A3R7SJC3_PENVA | 19066.334 | 271.09744 | 6 | 1 | Oxidation (M) |
| 170 | Beta-actin | Q9GSP9\|Q9GSP9_PENVA | 41867.86 | 523.0248 | 47 | 1 | Acetylation (N-term); Carbamidomethylation; Deamidation (NQ); Oxidation (M) |
| 171 | Cardiac muscle actin | A0A2H4V3C1\|A0A2H4V3C1_PENVA | 41709.797 | 520.44135 | 48 | 1 | Carbamidomethylation; Deamidation (NQ); Oxidation (M) |
| 172 | Projectin | A0A423T5K0\|A0A423T5K0_PENVA | 88011.305 | 518.2991 | 43 | 42 | Carbamidomethylation; Deamidation (NQ) |
| 173 | Cardiac muscle actin | A0A423TUJ4\|A0A423TUJ4_PENVA | 41735.785 | 514.3274 | 43 | 1 | Acetylation (N-term); Carbamidomethylation; Deamidation (NQ); Oxidation (M) |
| 174 | Actin 1 | A0A423SYA2\|A0A423SYA2_PENVA | 41824.94 | 514.2129 | 47 | 1 | Carbamidomethylation; Deamidation (NQ); Oxidation (M) |
| 175 | Actin 1 | A0A2H4V3F4\|A0A2H4V3F4_PENVA | 41724.758 | 510.79373 | 45 | 11 | Acetylation (N-term); Carbamidomethylation; Deamidation (NQ); Oxidation (M) |
| 176 | Hemolymph clottable protein | A0A423SNE1\|A0A423SNE1_PENVA | 187697.78 | 510.16754 | 43 | 27 | Carbamidomethylation; Deamidation (NQ); Oxidation (M) |
| 177 | Actin 1 | A0A3R7PR93\|A0A3R7PR93_PENVA | 41567.66 | 509.729 | 45 | 1 | Acetylation (N-term); Carbamidomethylation; Deamidation (NQ); Oxidation (M) |
| 178 | Calx-beta domain-containing protein | A0A3R7PHL7\|A0A3R7PHL7_PENVA | 75131.92 | 506.68198 | 39 | 34 | Carbamidomethylation; Deamidation (NQ); Oxidation (M) |
| 179 | Ankyrin-2-like | A0A423T6A9\|A0A423T6A9_PENVA | 310954.78 | 506.5759 | 39 | 39 | Carbamidomethylation |
| 180 | Ryanodine receptor | A0A3R7NF48\|A0A3R7NF48_PENVA | 146012.89 | 501.14148 | 39 | 39 | Carbamidomethylation; Deamidation (NQ) |
| 181 | Uncharacterized protein | A0A3R7PJD5\|A0A3R7PJD5_PENVA | 475050.75 | 62.205223 | 1 | 1 |  |
| 182 | Cardiac muscle actin | A0A3R7N8I3\|A0A3R7N8I3_PENVA | 81271.61 | 500.8769 | 40 | 1 | Carbamidomethylation; Deamidation (NQ); Oxidation (M) |
| 183 | Myosin heavy chain type a | A0A3R7PT74\|A0A3R7PT74_PENVA | 79462.07 | 496.48563 | 45 | 15 | Acetylation (N-term); Deamidation (NQ); Oxidation (M) |
| 184 | Putative troponin T, skeletal muscle isoform X3 | A0A423SQN7\|A0A423SQN7_PENVA | 43163.96 | 493.9473 | 46 | 46 | Acetylation (N-term); Carbamidomethylation; Deamidation (NQ); Oxidation (M) |
| 185 | Spectrin alpha chain | A0A3R7LUE4\|A0A3R7LUE4_PENVA | 277752.66 | 492.18756 | 35 | 35 | Carbamidomethylation; Deamidation (NQ); Oxidation (M) |
| 186 | Heat shock protein 70 | A0A423SX77\|A0A423SX77_PENVA | 151122.73 | 488.40466 | 40 | 19 | Carbamidomethylation; Deamidation (NQ); Oxidation (M) |
| 187 | Phosphofructokinase | A0A423T180\|A0A423T180_PENVA | 101230.76 | 488.2635 | 38 | 29 | Acetylation (N-term); Carbamidomethylation; Deamidation (NQ); Oxidation (M) |
| 188 | 6-phosphofructokinase (Fragment) | A0A423T143\|A0A423T143_PENVA | 19492.188 | 278.5778 | 6 | 6 |  |
| 189 | Myosin heavy chain type 2 | A0A423SX18\|A0A423SX18_PENVA | 54236.453 | 483.52396 | 37 | 2 | Acetylation (N-term); Deamidation (NQ); Oxidation (M) |
| 190 | Beta-actin | A0A423T2P8\|A0A423T2P8_PENVA | 41935.055 | 482.22708 | 35 | 1 | Acetylation (N-term); Carbamidomethylation; Deamidation (NQ); Oxidation (M) |
| 191 | Sarco/endoplasmic reticulum Ca2+-ATPase | A0A3R7QFA6\|A0A3R7QFA6_PENVA | 73466.836 | 481.22165 | 33 | 3 | Acetylation (N-term); Carbamidomethylation; Deamidation (NQ); Oxidation (M) |
| 192 | Troponin C (Fragment) | A0A423U7N4\|A0A423U7N4_PENVA | 10890.195 | 480.81284 | 33 | 20 | Acetylation (N-term); Deamidation (NQ); Oxidation (M) |
| 193 | Skeletal muscle actin 6 | A0A423T7C2\|A0A423T7C2_PENVA | 40028.164 | 479.77197 | 33 | 2 | Acetylation (N-term); Carbamidomethylation; Deamidation (NQ); Oxidation (M) |
| 194 | L-lactate dehydrogenase | I1VSB4\|I1VSB4_PENVA | 36022.406 | 478.69232 | 29 | 29 | Carbamidomethylation; Deamidation (NQ) |
| 195 | Putative basement membrane-specific heparan sulfate proteoglycan core protein isoform X11 | A0A3R7SUV3\|A0A3R7SUV3_PENVA | 273091.53 | 476.4418 | 32 | 32 | Carbamidomethylation; Deamidation (NQ) |
| 196 | Beta-actin | A0A3R7MT76\|A0A3R7MT76_PENVA | 41698.902 | 474.82394 | 34 | 1 | Acetylation (N-term); Carbamidomethylation; Deamidation (NQ); Oxidation (M) |
| 197 | Beta-1,3-glucan binding protein | A0A3R7PLM5\|A0A3R7PLM5_PENVA | 63529.652 | 474.6212 | 32 | 1 | Acetylation (N-term); Carbamidomethylation; Deamidation (NQ); Oxidation (M) |
| 198 | Putative myosin heavy chain, non-muscle-like | A0A3R7MQU7\|A0A3R7MQU7_PENVA | 174468.36 | 473.35782 | 30 | 30 | Carbamidomethylation; Deamidation (NQ); Oxidation (M) |
| 199 | Methyl-accepting transducer domain-containing protein | A0A423SH33\|A0A423SH33_PENVA | 267858.53 | 473.0436 | 32 | 32 | Carbamidomethylation; Deamidation (NQ) |
| 200 | Troponin C | A0A3R7QG92\|A0A3R7QG92_PENVA | 13625.268 | 472.32825 | 29 | 27 | Acetylation (N-term); Deamidation (NQ); Oxidation (M) |
| 201 | Putative calmodulin | A0A423U7E7\|A0A423U7E7_PENVA | 17928.975 | 472.03088 | 31 | 31 | Acetylation (N-term); Deamidation (NQ); Oxidation (M) |
| 202 | Gelsolin | A0A3R7MUZ7\|A0A3R7MUZ7_PENVA | 81848.586 | 463.92603 | 25 | 25 | Carbamidomethylation; Deamidation (NQ) |
| 203 | Nucleoside diphosphate kinase | A0A423TB87\|A0A423TB87_PENVA | 19231.453 | 462.5901 | 29 | 29 | Carbamidomethylation |
| 204 | fructose-bisphosphatase | A0A3R7P4D7\|A0A3R7P4D7_PENVA | 42054.09 | 456.11792 | 25 | 25 | Carbamidomethylation; Oxidation (M) |
| 205 | Uncharacterized protein | A0A3R7NDX5\|A0A3R7NDX5_PENVA | 92139.41 | 453.4298 | 24 | 22 |  |
| 206 | Phosphoglycerate mutase | A0A3R7LY50\|A0A3R7LY50_PENVA | 34367.508 | 452.1578 | 26 | 26 | Carbamidomethylation; Deamidation (NQ); Oxidation (M) |
| 207 | Bip | A0A3R7PER1\|A0A3R7PER1_PENVA | 71409.55 | 451.10794 | 26 | 1 | Acetylation (N-term); Deamidation (NQ) |
| 208 | Phosphoglycerate kinase | A0A423SCY3\|A0A423SCY3_PENVA | 44249.082 | 448.60648 | 25 | 25 | Carbamidomethylation; Deamidation (NQ) |
| 209 | Hemocyanin subunit L2 | A0A3R7NPL9\|A0A3R7NPL9_PENVA | 77578.37 | 447.24774 | 25 | 2 | Carbamidomethylation; Oxidation (M) |
| 210 | Isocitrate dehydrogenase [NADP] | A0A3R7SZS6\|A0A3R7SZS6_PENVA | 49746.797 | 441.32733 | 24 | 23 | Carbamidomethylation; Deamidation (NQ) |
| 211 | Exocyst complex component | A0A423SIK9\|A0A423SIK9_PENVA | 92001.15 | 74.05643 | 1 | 1 |  |
| 212 | USP domain-containing protein | A0A423SV65\|A0A423SV65_PENVA | 64112.293 | 74.05643 | 1 | 1 |  |
| 213 | Uncharacterized protein | A0A3R7M5J9\|A0A3R7M5J9_PENVA | 64004.965 | 438.27902 | 21 | 21 | Deamidation (NQ); Oxidation (M) |
| 214 | Uncharacterized protein | A0A3R7M4E5\|A0A3R7M4E5_PENVA | 48395.402 | 425.26514 | 19 | 19 | Deamidation (NQ); Oxidation (M) |
| 215 | ATP-dependent (S)-NAD(P)H-hydrate dehydratase | A0A423TEC0\|A0A423TEC0_PENVA | 32775.89 | 438.04547 | 23 | 23 | Carbamidomethylation; Deamidation (NQ); Oxidation (M) |
| 216 | Myosin regulatory light chain 2 | B7SNI3\|MLR_PENVA | 19269.156 | 436.17133 | 22 | 17 | Carbamidomethylation; Deamidation (NQ); Oxidation (M) |
| 217 | Actin, muscle | A0A3R7Q5M1\|A0A3R7Q5M1_PENVA | 19668.326 | 435.90533 | 22 | 2 | Deamidation (NQ); Oxidation (M) |
| 218 | Spectrin beta chain | A0A423U089\|A0A423U089_PENVA | 284158.84 | 435.07333 | 21 | 20 | Carbamidomethylation; Deamidation (NQ); Oxidation (M) |
| 219 | Hcy-binding domain-containing protein | A0A3R7M573\|A0A3R7M573_PENVA | 43965.184 | 430.76212 | 21 | 21 | Carbamidomethylation |
| 220 | Betaine--homocysteine S-methyltransferase 1 | A0A3R7QI48\|A0A3R7QI48_PENVA | 39667.25 | 119.20583 | 1 | 1 | Carbamidomethylation |
| 221 | Actin T2 | A0A3R7NT89\|A0A3R7NT89_PENVA | 15632.733 | 429.5103 | 23 | 1 | Acetylation (N-term); Carbamidomethylation; Deamidation (NQ) |
| 222 | Alpha-actinin, sarcomeric | A0A3R7SS49\|A0A3R7SS49_PENVA | 23990.74 | 427.30396 | 21 | 21 | Carbamidomethylation; Deamidation (NQ); Oxidation (M) |
| 223 | Na+/K+-ATPase alpha subunit | A0A423SWH3\|A0A423SWH3_PENVA | 134467.02 | 426.70654 | 20 | 20 | Carbamidomethylation; Deamidation (NQ); Oxidation (M) |
| 224 | Na+/K+-ATPase alpha subunit | A0A3R7MSD4\|A0A3R7MSD4_PENVA | 52821.67 | 335.4461 | 9 | 9 | Carbamidomethylation; Oxidation (M) |
| 225 | Isoform of A0A423SWH3, Na+/K+-ATPase alpha subunit | A0A423SWR4\|A0A423SWR4_PENVA | 39793.21 | 267.88522 | 5 | 5 | Carbamidomethylation |
| 226 | Putative titin isoform X4 | A0A423SNU7\|A0A423SNU7_PENVA | 389493.25 | 421.37244 | 20 | 20 | Acetylation (N-term); Carbamidomethylation; Deamidation (NQ) |
| 227 | Adenylate kinase | A0A3R7LZL7\|A0A3R7LZL7_PENVA | 24379.736 | 420.5795 | 19 | 19 | Carbamidomethylation; Oxidation (M) |
| 228 | Laminin subunit beta-1 | A0A423TBN1\|A0A423TBN1_PENVA | 182575.94 | 419.64093 | 19 | 19 | Carbamidomethylation; Deamidation (NQ) |
| 229 | DJ-1 protein | A0A3R7QGG2\|A0A3R7QGG2_PENVA | 16490.965 | 419.30896 | 19 | 19 |  |
| 230 | Fructose-bisphosphate aldolase | A0A3R7MEN4\|A0A3R7MEN4_PENVA | 42698.914 | 419.18 | 19 | 2 | Acetylation (N-term); Carbamidomethylation; Deamidation (NQ); Oxidation (M) |
| 231 | Histone H2A | A0A423SY25\|A0A423SY25_PENVA | 43524.51 | 417.533 | 20 | 2 | Carbamidomethylation; Deamidation (NQ); Oxidation (M) |
| 232 | Histone H2A/H2B/H3 domain-containing protein | A0A3R7LZQ6\|A0A3R7LZQ6_PENVA | 18365.389 | 332.375 | 9 | 2 | Carbamidomethylation; Deamidation (NQ); Oxidation (M) |
| 233 | Histone H2A/H2B/H3 domain-containing protein | A0A423T035\|A0A423T035_PENVA | 18280.322 | 332.375 | 9 | 2 | Carbamidomethylation; Deamidation (NQ); Oxidation (M) |
| 234 | Histone H2A/H2B/H3 domain-containing protein | A0A3R7QIZ4\|A0A3R7QIZ4_PENVA | 19377.455 | 332.375 | 9 | 2 | Carbamidomethylation; Deamidation (NQ); Oxidation (M) |
| 235 | Histone H2A/H2B/H3 domain-containing protein | A0A423SXP4\|A0A423SXP4_PENVA | 17103.967 | 332.375 | 9 | 2 | Carbamidomethylation; Deamidation (NQ); Oxidation (M) |
| 236 | Isoform of A0A423T035, Histone H3 | A0A3R7M8S0\|A0A3R7M8S0_PENVA | 15388.0205 | 332.375 | 9 | 2 | Carbamidomethylation; Deamidation (NQ); Oxidation (M) |
| 237 | Histone H3 | A0A3R7M2A5\|A0A3R7M2A5_PENVA | 17435.414 | 332.375 | 9 | 2 | Carbamidomethylation; Deamidation (NQ); Oxidation (M) |
| 238 | Histone H3 | A0A3R7LZR1\|A0A3R7LZR1_PENVA | 14718.285 | 332.375 | 9 | 2 | Carbamidomethylation; Deamidation (NQ); Oxidation (M) |
| 239 | Histone H3 | A0A423SXR9\|A0A423SXR9_PENVA | 18062.996 | 332.375 | 9 | 2 | Carbamidomethylation; Deamidation (NQ); Oxidation (M) |
| 240 | Histone H2A/H2B/H3 domain-containing protein | A0A423SXL1\|A0A423SXL1_PENVA | 28628.945 | 324.58594 | 8 | 2 | Carbamidomethylation; Deamidation (NQ); Oxidation (M) |
| 241 | Isoform of A0A423T035, Histone H3 | A0A423SXS5\|A0A423SXS5_PENVA | 12466.609 | 131.25304 | 2 | 1 |  |
| 242 | Putative Ca2+ sensor | A0A423SNS5\|A0A423SNS5_PENVA | 21988.86 | 412.9948 | 18 | 18 | Acetylation (N-term); Carbamidomethylation; Deamidation (NQ) |
| 243 | VWFD domain-containing protein | A0A3R7MYA0\|A0A3R7MYA0_PENVA | 48826.9 | 412.3812 | 17 | 17 | Carbamidomethylation |
| 244 | Small ribosomal subunit protein RACK1 | A0A3R7PEB6\|A0A3R7PEB6_PENVA | 35644.293 | 412.28897 | 17 | 17 | Carbamidomethylation |
| 245 | Putative myosin heavy chain, muscle-like | A0A3R7P1J2\|A0A3R7P1J2_PENVA | 17454.408 | 411.98703 | 21 | 3 | Acetylation (N-term); Deamidation (NQ) |
| 246 | Thioredoxin domain-containing protein | A0A423U8Z5\|A0A423U8Z5_PENVA | 77062.336 | 410.90308 | 19 | 19 | Acetylation (N-term); Carbamidomethylation; Deamidation (NQ); Oxidation (M) |
| 247 | Putative sarcalumenin | A0A3R7PEL2\|A0A3R7PEL2_PENVA | 107604.875 | 410.0846 | 17 | 17 | Acetylation (N-term) |
| 248 | Putative neural cell adhesion molecule 1 | A0A3R7QFI5\|A0A3R7QFI5_PENVA | 65036.086 | 410.0839 | 17 | 17 | Acetylation (N-term); Carbamidomethylation; Oxidation (M) |
| 249 | Adenosylhomocysteinase | A0A423SHX3\|A0A423SHX3_PENVA | 47449.45 | 409.4723 | 17 | 17 | Carbamidomethylation; Oxidation (M) |
| 250 | Isoform of A0A3R7N0A1, phosphoglucomutase (alpha-D-glucose-1,6-bisphosphate-dependent) | A0A3R7ST37\|A0A3R7ST37_PENVA | 74975.98 | 408.65527 | 19 | 19 | Carbamidomethylation; Deamidation (NQ) |
| 251 | phosphoglucomutase (alpha-D-glucose-1,6-bisphosphate-dependent) | A0A3R7N0A1\|A0A3R7N0A1_PENVA | 82132.39 | 408.65527 | 19 | 19 | Carbamidomethylation; Deamidation (NQ) |
| 252 | Myosin heavy chain | A0A3R7PV99\|A0A3R7PV99_PENVA | 14967.91 | 408.53824 | 19 | 2 | Acetylation (N-term); Carbamidomethylation; Oxidation (M) |
| 253 | Vitellogenin domain-containing protein | A0A3R7QMW3\|A0A3R7QMW3_PENVA | 70233.16 | 407.96832 | 17 | 17 | Carbamidomethylation; Deamidation (NQ) |
| 254 | SHSP domain-containing protein | A0A3R7PUW0\|A0A3R7PUW0_PENVA | 19864.309 | 407.6005 | 17 | 16 | Oxidation (M) |
| 255 | Heat shock cognate 70 | Q6GUA8\|Q6GUA8_PENVA | 71522.84 | 405.36517 | 20 | 3 | Acetylation (N-term); Deamidation (NQ) |
| 256 | Heat shock protein 70 | A0A423SC79\|A0A423SC79_PENVA | 73095.65 | 394.9553 | 18 | 3 | Acetylation (N-term); Deamidation (NQ) |
| 257 | Hsp70B | A0A3R7PTM6\|A0A3R7PTM6_PENVA | 76613.72 | 335.96475 | 11 | 1 | Acetylation (N-term); Deamidation (NQ) |
| 258 | Isoform of A0A3R7PTM6, Heat shock protein 70 | A0A3R7MHJ9\|A0A3R7MHJ9_PENVA | 70110.38 | 335.96475 | 11 | 1 | Acetylation (N-term); Deamidation (NQ) |
| 259 | Hemolymph clottable protein | A0A423SNP0\|A0A423SNP0_PENVA | 191164.5 | 405.05118 | 19 | 3 | Carbamidomethylation |
| 260 | Fibril-forming collagen alpha chain-like | A0A423TE44\|A0A423TE44_PENVA | 170358.5 | 402.72617 | 17 | 9 | Acetylation (N-term); Carbamidomethylation; Deamidation (NQ); Oxidation (M) |
| 261 | Putative hemicentin-1 isoform X2 | A0A3R7MML6\|A0A3R7MML6_PENVA | 125800.555 | 402.71838 | 17 | 16 | Carbamidomethylation; Deamidation (NQ) |
| 262 | Tubulin alpha chain | A0A423U8M4\|A0A423U8M4_PENVA | 49066.684 | 401.37546 | 18 | 3 | Carbamidomethylation; Oxidation (M) |
| 263 | Tubulin alpha chain | A0A423U5K7\|A0A423U5K7_PENVA | 41408.07 | 218.06186 | 3 | 3 |  |
| 264 | Tubulin alpha chain | A0A3R7M1P4\|A0A3R7M1P4_PENVA | 49819.273 | 399.42844 | 18 | 3 | Carbamidomethylation; Oxidation (M) |
| 265 | Elongation factor 2 | A0A423SKS4\|A0A423SKS4_PENVA | 94391.445 | 397.6146 | 17 | 17 | Carbamidomethylation; Deamidation (NQ) |
| 266 | Elongation factor 2 (Fragment) | A0A3R7QM81\|A0A3R7QM81_PENVA | 85071.41 | 357.34933 | 13 | 13 | Carbamidomethylation; Deamidation (NQ) |
| 267 | Neurofilament heavy polypeptide | A0A3R7T009\|A0A3R7T009_PENVA | 56246.652 | 397.1648 | 14 | 14 | Acetylation (N-term) |
| 268 | Myosin heavy chain type 6a | A0A3R7M673\|A0A3R7M673_PENVA | 18951.287 | 396.68222 | 17 | 1 | Acetylation (N-term); Deamidation (NQ) |
| 269 | Glucose-6-phosphate isomerase | A0A423SN06\|A0A423SN06_PENVA | 64461.723 | 395.98236 | 17 | 17 | Deamidation (NQ) |
| 270 | Tbp-associated factor | A0A3R7PM85\|A0A3R7PM85_PENVA | 32472.615 | 75.71503 | 1 | 1 |  |
| 271 | Uncharacterized protein | A0A3R7PJH1\|A0A3R7PJH1_PENVA | 37818.824 | 75.71503 | 1 | 1 |  |
| 272 | Laminin subunit alpha | A0A423TQC0\|A0A423TQC0_PENVA | 352258.94 | 395.23325 | 19 | 19 | Acetylation (N-term); Carbamidomethylation; Deamidation (NQ) |
| 273 | ADP/ATP translocase | A0A3R7MC29\|A0A3R7MC29_PENVA | 33427.957 | 395.0707 | 18 | 9 | Carbamidomethylation; Deamidation (NQ) |
| 274 | 14-3-3-like protein | A0A423TWV0\|A0A423TWV0_PENVA | 27922.164 | 393.2875 | 16 | 13 | Carbamidomethylation; Deamidation (NQ) |
| 275 | lactoylglutathione lyase | A0A423THJ4\|A0A423THJ4_PENVA | 31859.312 | 389.69577 | 15 | 15 | Acetylation (N-term); Carbamidomethylation; Deamidation (NQ); Oxidation (M) |
| 276 | Putative histidine triad nucleotide-binding protein 2, mitochondrial-like | A0A3R7MYQ8\|A0A3R7MYQ8_PENVA | 18924.955 | 387.11096 | 13 | 13 | Carbamidomethylation; Oxidation (M) |
| 277 | Adenylosuccinate synthetase | A0A423SPS9\|A0A423SPS9_PENVA | 41451.438 | 386.036 | 16 | 16 | Carbamidomethylation; Deamidation (NQ) |
| 278 | Farnesoic acid O-methyltransferase (Fragment) | A0A3R7PCP7\|A0A3R7PCP7_PENVA | 38581.965 | 386.01428 | 14 | 14 | Acetylation (N-term); Carbamidomethylation; Oxidation (M) |
| 279 | Putative fibrillin-2-like | A0A3R7PW33\|A0A3R7PW33_PENVA | 162585.56 | 384.98306 | 14 | 14 | Carbamidomethylation; Deamidation (NQ); Oxidation (M) |
| 280 | Pacifastin light chain (Fragment) | A0A423SZH0\|A0A423SZH0_PENVA | 135718.64 | 384.9546 | 13 | 13 | Carbamidomethylation; Deamidation (NQ) |
| 281 | Pacifastin light chain | A0A423TBV9\|A0A423TBV9_PENVA | 104738.11 | 307.3232 | 7 | 7 | Carbamidomethylation; Deamidation (NQ) |
| 282 | Putative Thrombospondin-3 | A0A3R7PYP1\|A0A3R7PYP1_PENVA | 59261.88 | 384.80362 | 14 | 14 | Carbamidomethylation; Deamidation (NQ) |
| 283 | Transcription factor BTF3 (Fragment) | A0A3R7LS46\|A0A3R7LS46_PENVA | 16768.803 | 384.1143 | 15 | 15 | Deamidation (NQ) |
| 284 | Myosin light chain | A0A3R7M961\|A0A3R7M961_PENVA | 17334.049 | 383.90463 | 15 | 15 | Acetylation (N-term); Carbamidomethylation; Deamidation (NQ); Oxidation (M) |
| 285 | Lamin Dm0 | A0A3R7Q7T6\|A0A3R7Q7T6_PENVA | 52350.855 | 382.82925 | 18 | 17 | Carbamidomethylation; Deamidation (NQ); Oxidation (M) |
| 286 | Heat shock protein 21 | A0A3R7NN04\|A0A3R7NN04_PENVA | 84741.945 | 382.80713 | 14 | 14 | Carbamidomethylation; Deamidation (NQ) |
| 287 | Hemocyanin | A0A3R7P7W3\|A0A3R7P7W3_PENVA | 77391.82 | 382.4551 | 15 | 3 | Carbamidomethylation; Oxidation (M) |
| 288 | 10-formyltetrahydrofolate dehydrogenase | A0A3R7PD39\|A0A3R7PD39_PENVA | 99886.59 | 378.39777 | 14 | 14 | Carbamidomethylation |
| 289 | ATP synthase subunit alpha | A0A423TIL1\|A0A423TIL1_PENVA | 59306.52 | 376.96478 | 14 | 14 |  |
| 290 | LIM domain-binding protein 3 | A0A3R7SYN0\|A0A3R7SYN0_PENVA | 53323.76 | 375.9522 | 12 | 12 | Carbamidomethylation |
| 291 | Protein disulfide-isomerase | A0A423U5I3\|A0A423U5I3_PENVA | 57496.977 | 375.43335 | 12 | 12 |  |
| 292 | Protein disulfide isomerase | A0A3R7LYM3\|A0A3R7LYM3_PENVA | 35849.66 | 327.49625 | 8 | 8 |  |
| 293 | 6-phosphofructokinase | A0A423T0V9\|A0A423T0V9_PENVA | 80951.41 | 375.00085 | 13 | 4 | Carbamidomethylation; Deamidation (NQ) |
| 294 | Uncharacterized protein | A0A423SK60\|A0A423SK60_PENVA | 46753.117 | 374.74963 | 13 | 13 |  |
| 295 | Ryanodine receptor | A0A423U9Z8\|A0A423U9Z8_PENVA | 55530.203 | 373.82422 | 13 | 13 | Carbamidomethylation |
| 296 | Uncharacterized protein | A0A3R7LZ71\|A0A3R7LZ71_PENVA | 39529.215 | 371.5862 | 12 | 12 | Carbamidomethylation; Deamidation (NQ) |
| 297 | Calreticulin | A0A3R7PYG5\|A0A3R7PYG5_PENVA | 46871.426 | 369.67752 | 12 | 12 | Carbamidomethylation; Deamidation (NQ) |
| 298 | Putative calcium-activated chloride channel regulator 2 | A0A3R7ML54\|A0A3R7ML54_PENVA | 62320.4 | 367.37784 | 12 | 10 |  |
| 299 | Putative elongation factor 1-beta | A0A3R7Q0U5\|A0A3R7Q0U5_PENVA | 24016.889 | 365.9375 | 12 | 11 | Carbamidomethylation |
| 300 | Laminin subunit gamma-1 | A0A3R7NXU5\|A0A3R7NXU5_PENVA | 140415.81 | 365.5814 | 12 | 12 | Acetylation (N-term); Carbamidomethylation |
| 301 | Na+/Ca2+-exchanger | A0A3R7SVL4\|A0A3R7SVL4_PENVA | 38690.44 | 365.07852 | 12 | 7 | Deamidation (NQ) |
| 302 | Eukaryotic translation initiation factor 5A | M9WSX9\|M9WSX9_PENVA | 17257.557 | 364.63 | 13 | 13 |  |
| 303 | Tubulin alpha chain | A0A423SD42\|A0A423SD42_PENVA | 48379.76 | 363.32333 | 13 | 2 | Carbamidomethylation; Oxidation (M) |
| 304 | Muscle LIM protein Mlp84B | A0A3R7QRP4\|A0A3R7QRP4_PENVA | 20432.58 | 361.7899 | 11 | 11 | Carbamidomethylation; Deamidation (NQ); Oxidation (M) |
| 305 | Talin-1 | A0A423SL60\|A0A423SL60_PENVA | 199854.14 | 361.28986 | 12 | 12 | Carbamidomethylation |
| 306 | Trichohyalin | A0A3R7QGD6\|A0A3R7QGD6_PENVA | 225588.84 | 360.1665 | 16 | 16 | Carbamidomethylation; Deamidation (NQ) |
| 307 | Uncharacterized protein | A0A3R7QW30\|A0A3R7QW30_PENVA | 47251.887 | 359.31046 | 11 | 11 | Acetylation (N-term); Deamidation (NQ) |
| 308 | Glycogen debranching enzyme (Fragment) | A0A3R7MD67\|A0A3R7MD67_PENVA | 134800.42 | 354.05545 | 13 | 13 | Deamidation (NQ) |
| 309 | Putative glycogen debranching enzyme isoform X1 | A0A3R7QAY0\|A0A3R7QAY0_PENVA | 117268.57 | 341.75964 | 12 | 12 | Deamidation (NQ) |
| 310 | Elongation factor 1-delta protein | A0A3R7LWV0\|A0A3R7LWV0_PENVA | 38844.215 | 353.79242 | 11 | 10 | Acetylation (N-term); Carbamidomethylation |
| 311 | Tubulin beta-2 chain | A0A3R7MGX0\|A0A3R7MGX0_PENVA | 63529.055 | 352.80746 | 11 | 1 |  |
| 312 | Putative tubulin beta-1 chain | A0A3R7QA79\|A0A3R7QA79_PENVA | 87862.47 | 352.80746 | 11 | 1 |  |
| 313 | Putative isochorismatase domain-containing protein 2, mitochondrial-like | A0A423SAB7\|A0A423SAB7_PENVA | 24006.941 | 351.88467 | 10 | 10 | Carbamidomethylation |
| 314 | Dystonin | A0A423TTR8\|A0A423TTR8_PENVA | 405597.2 | 351.0616 | 15 | 15 | Acetylation (N-term); Deamidation (NQ) |
| 315 | Transient receptor potential channel pyrexia | A0A3R7PES4\|A0A3R7PES4_PENVA | 94439.85 | 39.783207 | 1 | 1 |  |
| 316 | RNA exonuclease 4 | A0A423SPR7\|A0A423SPR7_PENVA | 38838.48 | 39.783207 | 1 | 1 |  |
| 317 | Ig-like domain-containing protein | A0A423SZM8\|A0A423SZM8_PENVA | 35096.92 | 351.04968 | 11 | 11 | Carbamidomethylation |
| 318 | EH domain-containing protein 1 | A0A3R7N5R2\|A0A3R7N5R2_PENVA | 58694.58 | 350.94003 | 10 | 10 |  |
| 319 | Reticulon-like protein | A0A3R7M9D9\|A0A3R7M9D9_PENVA | 18270.29 | 350.12576 | 10 | 10 | Deamidation (NQ) |
| 320 | cystathionine gamma-lyase | A0A423TEZ4\|A0A423TEZ4_PENVA | 42363.473 | 348.09937 | 11 | 11 | Acetylation (N-term); Carbamidomethylation |
| 321 | Gastrolith protein 30 | A0A3R7NXR2\|A0A3R7NXR2_PENVA | 26521.52 | 347.26965 | 10 | 10 | Carbamidomethylation; Deamidation (NQ) |
| 322 | Polyubiquitin | A0A423TTK6\|A0A423TTK6_PENVA | 96278.85 | 346.69376 | 10 | 10 | Deamidation (NQ) |
| 323 | Polyubiquitin-C | A0A3R7Q158\|A0A3R7Q158_PENVA | 111212.89 | 346.69376 | 10 | 10 | Deamidation (NQ) |
| 324 | >sp\|RS27A_HUMAN\| | #CONTAM#RS27A_HUMAN\| | 17964.914 | 334.59195 | 9 | 9 | Deamidation (NQ) |
| 325 | Ubiquitin-like domain-containing protein | A0A423SIM8\|A0A423SIM8_PENVA | 37876.38 | 334.59195 | 9 | 9 | Deamidation (NQ) |
| 326 | Ubiquitin-ribosomal protein eL40 fusion protein | A0A076NBT3\|A0A076NBT3_PENVA | 14701.281 | 334.59195 | 9 | 9 | Deamidation (NQ) |
| 327 | Isoform of A0A423SIM8, Ubiquitin-like domain-containing protein | A0A423TU39\|A0A423TU39_PENVA | 37573.16 | 334.59195 | 9 | 9 | Deamidation (NQ) |
| 328 | Trichohyalin-like | A0A3R7QX89\|A0A3R7QX89_PENVA | 325758.7 | 344.68573 | 12 | 12 | Deamidation (NQ); Oxidation (M) |
| 329 | Uncharacterized protein | A0A423TCL6\|A0A423TCL6_PENVA | 80286.97 | 344.31506 | 11 | 11 | Acetylation (N-term); Deamidation (NQ) |
| 330 | Malate dehydrogenase | A0A3R7PNK7\|A0A3R7PNK7_PENVA | 54015.027 | 343.37738 | 10 | 10 | Carbamidomethylation |
| 331 | Beta-1,3-glucan-binding protein | A0A3R7PI18\|A0A3R7PI18_PENVA | 60414.867 | 340.96014 | 10 | 10 |  |
| 332 | Histone H3 | A0A3R7MJT5\|A0A3R7MJT5_PENVA | 15281.881 | 340.85504 | 10 | 3 | Carbamidomethylation |
| 333 | Putative histone H3.3-like isoform X2 | A0A423SFE3\|A0A423SFE3_PENVA | 12407.448 | 340.85504 | 10 | 3 | Carbamidomethylation |
| 334 | Histone H3 | A0A3R7P8P2\|A0A3R7P8P2_PENVA | 15297.881 | 340.85504 | 10 | 3 | Carbamidomethylation |
| 335 | Histone H3 | A0A3R7PTG8\|A0A3R7PTG8_PENVA | 15327.907 | 340.85504 | 10 | 3 | Carbamidomethylation |
| 336 | Histone H3 | A0A423TQD7\|A0A423TQD7_PENVA | 15369.944 | 340.85504 | 10 | 3 | Carbamidomethylation |
| 337 | Core histone H2A/H2B/H3/H4 | A0A3R7MIH4\|A0A3R7MIH4_PENVA | 17047.838 | 340.85504 | 10 | 3 | Carbamidomethylation |
| 338 | Isoform of A0A423STH3, Potassium channel | A0A3R7N7D2\|A0A3R7N7D2_PENVA | 49675.41 | 340.65158 | 10 | 7 |  |
| 339 | Ig-like domain-containing protein | A0A423SN57\|A0A423SN57_PENVA | 35441.785 | 337.94083 | 9 | 9 | Carbamidomethylation |
| 340 | GMP reductase | A0A423SS90\|A0A423SS90_PENVA | 35744.727 | 336.75638 | 10 | 10 | Carbamidomethylation; Oxidation (M) |
| 341 | Prefoldin subunit 3 | A0A423SYQ1\|A0A423SYQ1_PENVA | 21065.336 | 335.29297 | 10 | 10 | Deamidation (NQ); Oxidation (M) |
| 342 | Fibril-forming collagen alpha chain-like (Fragment) | A0A423T2L9\|A0A423T2L9_PENVA | 167315.5 | 334.6202 | 9 | 1 | Carbamidomethylation; Oxidation (M) |
| 343 | ATP synthase subunit beta | A0A423U5W2\|A0A423U5W2_PENVA | 55489.355 | 334.20746 | 12 | 8 | Acetylation (N-term); Deamidation (NQ) |
| 344 | Large ribosomal subunit protein uL18 | A0A3R7MQM8\|A0A3R7MQM8_PENVA | 34748.96 | 333.66302 | 9 | 9 | Deamidation (NQ); Oxidation (M) |
| 345 | Heat shock protein 70 | A0A423SCR7\|A0A423SCR7_PENVA | 66887.266 | 333.3596 | 11 | 1 | Carbamidomethylation; Deamidation (NQ); Oxidation (M) |
| 346 | Uncharacterized protein | A0A423SK95\|A0A423SK95_PENVA | 59585.42 | 332.70627 | 10 | 10 | Deamidation (NQ) |
| 347 | Putative integrator complex subunit 6-B isoform X2 | A0A3R7PPC3\|A0A3R7PPC3_PENVA | 128845.88 | 38.717575 | 1 | 1 |  |
| 348 | ADP/ATP translocase | A0A3R7QA52\|A0A3R7QA52_PENVA | 25957.994 | 332.0904 | 11 | 2 | Carbamidomethylation; Deamidation (NQ) |
| 349 | Methenyltetrahydrofolate synthase domain-containing protein (Fragment) | A0A3R7Q0U0\|A0A3R7Q0U0_PENVA | 62011.152 | 331.9126 | 9 | 9 | Carbamidomethylation |
| 350 | Putative methenyltetrahydrofolate synthase domain-containing protein isoform X2 | A0A423U7P1\|A0A423U7P1_PENVA | 23059.463 | 223.43153 | 3 | 3 | Carbamidomethylation |
| 351 | Prolyl endopeptidase | A0A423U6S1\|A0A423U6S1_PENVA | 76420.94 | 330.81393 | 9 | 9 | Carbamidomethylation; Deamidation (NQ) |
| 352 | vesicle-fusing ATPase | A0A423TPP0\|A0A423TPP0_PENVA | 88206.7 | 327.60764 | 9 | 9 | Acetylation (N-term); Carbamidomethylation; Deamidation (NQ) |
| 353 | Golgin subfamily A member 4 | A0A3R7MLD2\|A0A3R7MLD2_PENVA | 87804.07 | 326.80673 | 10 | 10 | Carbamidomethylation; Deamidation (NQ) |
| 354 | Uncharacterized protein | A0A3R7PX44\|A0A3R7PX44_PENVA | 56483.477 | 309.51956 | 8 | 8 | Carbamidomethylation; Deamidation (NQ) |
| 355 | Prefoldin subunit 4 | A0A3R7Q9V6\|A0A3R7Q9V6_PENVA | 14847.516 | 324.20093 | 8 | 8 | Oxidation (M) |
| 356 | Cofilin/actin-depolymerizing factor | A0A3R7Q305\|A0A3R7Q305_PENVA | 17131.445 | 324.124 | 8 | 8 | Carbamidomethylation |
| 357 | Annexin | A0A3R7QA34\|A0A3R7QA34_PENVA | 35699.91 | 322.56546 | 8 | 8 |  |
| 358 | Heat shock protein 70 kDa | A0A3R7PWV9\|A0A3R7PWV9_PENVA | 41918.176 | 322.39438 | 11 | 2 | Carbamidomethylation; Deamidation (NQ) |
| 359 | Polysialoglycoprotein | A0A423SUV4\|A0A423SUV4_PENVA | 65036.98 | 322.1865 | 9 | 8 | Acetylation (N-term) |
| 360 | Isoform of A0A423SQX9, Putative muscle M-line assembly protein unc-89-like isoform X2 (Fragment) | A0A423TKI9\|A0A423TKI9_PENVA | 153443.92 | 321.6192 | 8 | 8 |  |
| 361 | Glycerol-3-phosphate dehydrogenase [NAD(+)] | A0A3R7PAZ3\|A0A3R7PAZ3_PENVA | 33638 | 319.87576 | 10 | 10 | Carbamidomethylation |
| 362 | proton-translocating NAD(P)(+) transhydrogenase | A0A3R7QM12\|A0A3R7QM12_PENVA | 113381.58 | 319.68576 | 10 | 10 | Carbamidomethylation |
| 363 | proton-translocating NAD(P)(+) transhydrogenase | A0A423SNG3\|A0A423SNG3_PENVA | 62444.695 | 247.23222 | 5 | 5 | Carbamidomethylation |
| 364 | Isoform of A0A3R7QM12, Proton-translocating NAD(P)(+) transhydrogenase | A0A423SNQ1\|A0A423SNQ1_PENVA | 43940.727 | 148.32744 | 3 | 3 |  |
| 365 | Titin (Fragment) | A0A423SZ32\|A0A423SZ32_PENVA | 44999.418 | 317.0591 | 7 | 7 | Carbamidomethylation; Deamidation (NQ) |
| 366 | RhoA | A0A3R7QNM6\|A0A3R7QNM6_PENVA | 21605.156 | 316.77472 | 9 | 8 | Carbamidomethylation |
| 367 | RhoA | A0A3R7Q0A1\|A0A3R7Q0A1_PENVA | 37863.438 | 316.77472 | 9 | 8 | Carbamidomethylation |
| 368 | Uncharacterized protein | A0A3R7QAY8\|A0A3R7QAY8_PENVA | 16290.256 | 316.4548 | 19 | 19 | Oxidation (M) |
| 369 | Uncharacterized protein | A0A3R7PIL9\|A0A3R7PIL9_PENVA | 62140.047 | 315.86752 | 7 | 7 |  |
| 370 | UV excision repair protein RAD23 | A0A423SMA6\|A0A423SMA6_PENVA | 30634.557 | 315.84137 | 8 | 8 | Oxidation (M) |
| 371 | UV excision repair protein RAD23 | A0A3R7PG05\|A0A3R7PG05_PENVA | 13099.478 | 273.4512 | 5 | 5 |  |
| 372 | Leucine-rich repeat flightless-I-interacting protein 2B (Fragment) | A0A3R7MHC2\|A0A3R7MHC2_PENVA | 43443.37 | 312.23038 | 7 | 7 | Deamidation (NQ) |
| 373 | Troponin C isoform 3 | A0A423SVU5\|A0A423SVU5_PENVA | 17488.393 | 311.3428 | 7 | 1 | Deamidation (NQ) |
| 374 | Projectin | A0A3R7M4M5\|A0A3R7M4M5_PENVA | 52522.375 | 311.27826 | 8 | 8 | Deamidation (NQ); Oxidation (M) |
| 375 | Muscle M-line assembly protein unc-89 | A0A423U095\|A0A423U095_PENVA | 60829.453 | 310.105 | 9 | 9 |  |
| 376 | Putative Kv channel-interacting protein 4-like isoform X2 | A0A423TFU5\|A0A423TFU5_PENVA | 59667.71 | 38.187443 | 1 | 1 |  |
| 377 | Putative gamma-aminobutyric acid receptor subunit delta | A0A3R7QU60\|A0A3R7QU60_PENVA | 77510.92 | 38.187443 | 1 | 1 |  |
| 378 | Serine/threonine-protein kinase ULK3 | A0A3R7QK05\|A0A3R7QK05_PENVA | 54703.75 | 38.187443 | 1 | 1 |  |
| 379 | C-1-tetrahydrofolate synthase, cytoplasmic | A0A423UB03\|A0A423UB03_PENVA | 100556.96 | 38.187443 | 1 | 1 |  |
| 380 | C-type lectin domain-containing protein | A0A423TMM3\|A0A423TMM3_PENVA | 63790.227 | 38.187443 | 1 | 1 |  |
| 381 | ABC protein, subfamily ABCG | A0A423TTF4\|A0A423TTF4_PENVA | 75360.76 | 38.187443 | 1 | 1 |  |
| 382 | C-1-tetrahydrofolate synthase, cytoplasmic | A0A3R7SL78\|A0A3R7SL78_PENVA | 100540.96 | 38.187443 | 1 | 1 |  |
| 383 | 3-methyladenine DNA glycosidase | A0A3R7QNH0\|A0A3R7QNH0_PENVA | 55625.414 | 38.187443 | 1 | 1 |  |
| 384 | Acyl-CoA-binding protein | A0A3R7PDI4\|A0A3R7PDI4_PENVA | 10136.464 | 309.9558 | 7 | 7 |  |
| 385 | S-phase kinase-associated protein 1 | A0A423T4X7\|A0A423T4X7_PENVA | 18907.283 | 309.6443 | 8 | 8 | Carbamidomethylation; Deamidation (NQ) |
| 386 | Prefoldin subunit | A0A423THR5\|A0A423THR5_PENVA | 18324.152 | 308.86603 | 8 | 8 | Carbamidomethylation |
| 387 | Prefoldin subunit | A0A3R7MGP5\|A0A3R7MGP5_PENVA | 14754.094 | 281.93182 | 6 | 6 |  |
| 388 | Heat shock protein 83 | A0A423TIC0\|A0A423TIC0_PENVA | 88172.16 | 305.77866 | 8 | 8 | Deamidation (NQ) |
| 389 | Heat shock protein 83 | A0A423TI28\|A0A423TI28_PENVA | 59137.516 | 166.3933 | 3 | 3 | Deamidation (NQ) |
| 390 | Proteasome subunit beta | A0A3R7NZW3\|A0A3R7NZW3_PENVA | 22979.535 | 303.68463 | 7 | 7 | Carbamidomethylation |
| 391 | Annulin | A0A423T6S9\|A0A423T6S9_PENVA | 86330.37 | 303.66727 | 7 | 7 | Carbamidomethylation |
| 392 | Receptor-type tyrosine-protein phosphatase kappa | A0A423THA1\|A0A423THA1_PENVA | 54141.504 | 302.86783 | 7 | 7 | Deamidation (NQ) |
| 393 | ES1-like protein, mitochondrial | A0A423TX84\|A0A423TX84_PENVA | 26242.912 | 302.7532 | 8 | 8 | Carbamidomethylation |
| 394 | Charged multivesicular body protein 4b | A0A3R7PXM5\|A0A3R7PXM5_PENVA | 24461.412 | 301.19418 | 7 | 7 |  |
| 395 | Glutamate gated chloride channel | A0A3R7LRM3\|A0A3R7LRM3_PENVA | 45800.934 | 300.82602 | 7 | 7 |  |
| 396 | Putative glutamate-gated chloride channel isoform X4 | A0A3R7T1Q9\|A0A3R7T1Q9_PENVA | 47634.105 | 187.69495 | 3 | 3 |  |
| 397 | Uncharacterized protein | A0A3R7PI34\|A0A3R7PI34_PENVA | 14536.308 | 180.10765 | 2 | 2 |  |
| 398 | Eukaryotic initiation factor 4E binding protein | A0A3R7PEN3\|A0A3R7PEN3_PENVA | 12642.262 | 299.97467 | 8 | 8 | Deamidation (NQ) |
| 399 | CaMKII isoform E | A0A423TRR1\|A0A423TRR1_PENVA | 22144.723 | 299.31714 | 6 | 6 | Carbamidomethylation |
| 400 | Putative tubulin alpha-1B chain isoform X5 | A0A423SVJ1\|A0A423SVJ1_PENVA | 28921.79 | 299.14078 | 8 | 1 | Carbamidomethylation; Oxidation (M) |
| 401 | Uncharacterized protein | A0A3R7M5R5\|A0A3R7M5R5_PENVA | 298903.38 | 298.62854 | 7 | 6 |  |
| 402 | Serine/threonine-protein phosphatase | A0A423SHB2\|A0A423SHB2_PENVA | 40635.58 | 298.33667 | 7 | 5 | Carbamidomethylation |
| 403 | Serine/threonine-protein phosphatase | A0A3R7PN62\|A0A3R7PN62_PENVA | 31528.938 | 118.198944 | 1 | 1 | Carbamidomethylation |
| 404 | Moesin/ezrin/radixin homolog 1 | A0A3R7PAT7\|A0A3R7PAT7_PENVA | 78752.97 | 297.85257 | 9 | 8 | Carbamidomethylation; Deamidation (NQ) |
| 405 | Moesin/ezrin/radixin homolog 1 | A0A3R7QBI0\|A0A3R7QBI0_PENVA | 51733.598 | 114.62679 | 2 | 2 |  |
| 406 | Moesin/ezrin/radixin homolog 1 | A0A3R7ST02\|A0A3R7ST02_PENVA | 70382.55 | 114.62679 | 2 | 2 |  |
| 407 | E1 ubiquitin-activating enzyme | A0A423U7J2\|A0A423U7J2_PENVA | 112770.94 | 296.73132 | 6 | 6 |  |
| 408 | Xylose isomerase | A0A3R7PJ76\|A0A3R7PJ76_PENVA | 70005.04 | 296.0934 | 6 | 6 | Deamidation (NQ) |
| 409 | Alpha-taxilin | A0A423U3L3\|A0A423U3L3_PENVA | 71926.34 | 295.5772 | 8 | 7 | Deamidation (NQ) |
| 410 | Carboxypeptidase B | A0A3R7SYN4\|A0A3R7SYN4_PENVA | 25693.117 | 295.29135 | 6 | 6 | Carbamidomethylation; Deamidation (NQ) |
| 411 | Tubulin beta chain | A0A423SID4\|A0A423SID4_PENVA | 50799.227 | 295.1239 | 8 | 1 | Carbamidomethylation |
| 412 | Tubulin beta chain | A0A423THU9\|A0A423THU9_PENVA | 50125.633 | 207.90004 | 3 | 1 |  |
| 413 | Malate dehydrogenase | A0A423T5Y1\|A0A423T5Y1_PENVA | 35284.8 | 294.92932 | 6 | 6 |  |
| 414 | Protein-L-isoaspartate(D-aspartate) O-methyltransferase | A0A3R7MBQ4\|A0A3R7MBQ4_PENVA | 24855.414 | 294.81226 | 7 | 7 |  |
| 415 | Collagen alpha-1(V) chain | A0A423SAL8\|A0A423SAL8_PENVA | 109056.445 | 293.42282 | 6 | 6 |  |
| 416 | Putative proteasome subunit alpha type-6-like | A0A3R7LSG6\|A0A3R7LSG6_PENVA | 25068.639 | 293.28113 | 7 | 7 | Carbamidomethylation |
| 417 | Proteasome subunit alpha type | A0A3R7P5H5\|A0A3R7P5H5_PENVA | 26852.43 | 293.28113 | 7 | 7 | Carbamidomethylation |
| 418 | Crustacyanin subunit A | A0A3R7MIN0\|A0A3R7MIN0_PENVA | 24074.084 | 292.83817 | 7 | 1 | Carbamidomethylation; Deamidation (NQ) |
| 419 | Crustacyanin subunit A | A0A423SEP1\|A0A423SEP1_PENVA | 21104.625 | 289.0863 | 6 | 1 | Carbamidomethylation |
| 420 | Putative thrombospondin type-1 domain-containing protein 4-like | A0A3R7P5N9\|A0A3R7P5N9_PENVA | 88524.875 | 291.6241 | 6 | 6 | Carbamidomethylation |
| 421 | PLAC domain-containing protein | A0A423TIY7\|A0A423TIY7_PENVA | 27605.428 | 248.26588 | 4 | 4 | Carbamidomethylation |
| 422 | glutamate dehydrogenase [NAD(P)(+)] | A0A3R7PGP1\|A0A3R7PGP1_PENVA | 82374.29 | 291.24625 | 6 | 6 | Carbamidomethylation |
| 423 | Nidogen-2 | A0A423S9W3\|A0A423S9W3_PENVA | 31805.516 | 291.0988 | 6 | 6 | Carbamidomethylation |
| 424 | Phosphorylase b kinase regulatory subunit | A0A3R7QP64\|A0A3R7QP64_PENVA | 71852.38 | 287.96835 | 6 | 6 |  |
| 425 | Putative oligoribonuclease, mitochondrial | A0A3R7SHS1\|A0A3R7SHS1_PENVA | 24625.402 | 287.9343 | 6 | 6 |  |
| 426 | 60S ribosomal protein L7a | A0A423SSD7\|A0A423SSD7_PENVA | 35589.914 | 287.28693 | 6 | 6 | Carbamidomethylation |
| 427 | Mitochondrial ATP synthase delta subunit | H9LFA4\|H9LFA4_PENVA | 16498.586 | 286.8145 | 6 | 6 |  |
| 428 | Elongation factor 1-alpha | A0A3R7NRQ6\|A0A3R7NRQ6_PENVA | 32271.703 | 285.6677 | 6 | 6 | Carbamidomethylation; Deamidation (NQ) |
| 429 | Elongation factor 1-alpha | A0A423TZA0\|A0A423TZA0_PENVA | 30005.361 | 222.69308 | 3 | 3 | Carbamidomethylation |
| 430 | Crustacyanin subunit A | A0A423SEM5\|A0A423SEM5_PENVA | 23154.709 | 285.65268 | 7 | 1 | Carbamidomethylation; Deamidation (NQ) |
| 431 | Crustacyanin subunit A | A0A3R7P6W3\|A0A3R7P6W3_PENVA | 21219.717 | 285.65268 | 7 | 1 | Carbamidomethylation; Deamidation (NQ) |
| 432 | Beta-1,3-glucan-binding protein | A0A3R7M1N6\|A0A3R7M1N6_PENVA | 104661.49 | 284.15106 | 10 | 8 | Deamidation (NQ) |
| 433 | Sodium/calcium exchanger | A0A423TLI4\|A0A423TLI4_PENVA | 20960.56 | 283.88834 | 8 | 8 |  |
| 434 | Sodium/calcium exchanger 1 | A0A423T6F9\|A0A423T6F9_PENVA | 25357.906 | 184.34447 | 4 | 4 |  |
| 435 | Smoothelin domain-containing protein | A0A3R7PSG3\|A0A3R7PSG3_PENVA | 329829.2 | 283.7228 | 9 | 9 | Acetylation (N-term); Deamidation (NQ) |
| 436 | Copper/zinc superoxide dismutase isoform 4 | A0A423TH92\|A0A423TH92_PENVA | 70158.58 | 282.53854 | 7 | 7 | Carbamidomethylation; Deamidation (NQ) |
| 437 | Trans-sialidase | A0A423TNX4\|A0A423TNX4_PENVA | 91971.305 | 281.88324 | 5 | 5 |  |
| 438 | Uncharacterized protein | A0A423TNZ4\|A0A423TNZ4_PENVA | 71867.766 | 281.88324 | 5 | 5 |  |
| 439 | Uncharacterized protein (Fragment) | A0A3R7NP30\|A0A3R7NP30_PENVA | 43886.656 | 281.32138 | 5 | 5 |  |
| 440 | Fatty-acid binding protein | E2IH93\|E2IH93_PENVA | 15494.823 | 280.2511 | 7 | 7 | Acetylation (N-term); Deamidation (NQ) |
| 441 | Laminin G domain-containing protein | A0A3R7MDX3\|A0A3R7MDX3_PENVA | 75196.96 | 279.2391 | 6 | 6 | Deamidation (NQ) |
| 442 | Lacunin | A0A423SN99\|A0A423SN99_PENVA | 43733.59 | 279.22495 | 5 | 5 | Carbamidomethylation |
| 443 | Isoform of A0A3R7PSB0, Putative trimeric intracellular cation channel type A | A0A3R7LWC0\|A0A3R7LWC0_PENVA | 16024.662 | 278.7716 | 5 | 5 | Acetylation (N-term) |
| 444 | Cathepsin F-like cysteine peptidase protein | A0A3R7PSB0\|A0A3R7PSB0_PENVA | 46103.656 | 122.313835 | 1 | 1 | Acetylation (N-term) |
| 445 | Putative calumenin | A0A3R7PEJ3\|A0A3R7PEJ3_PENVA | 21389.018 | 278.13312 | 5 | 5 | Deamidation (NQ) |
| 446 | Putative calcium/calmodulin-dependent protein kinase type II alpha chain isoform X10 | A0A3R7MG20\|A0A3R7MG20_PENVA | 38766.434 | 277.52902 | 5 | 5 | Carbamidomethylation |
| 447 | Uncharacterized protein | A0A423TNY3\|A0A423TNY3_PENVA | 11040.31 | 277.41144 | 6 | 6 |  |
| 448 | Proteasome subunit alpha type | A0A3R7PNG1\|A0A3R7PNG1_PENVA | 26213.729 | 276.77368 | 5 | 5 |  |
| 449 | VAMP-associated protein | A0A423TKA1\|A0A423TKA1_PENVA | 27170.191 | 276.58844 | 5 | 5 |  |
| 450 | Phosphorylase b kinase regulatory subunit | A0A423T8A3\|A0A423T8A3_PENVA | 115329.94 | 275.32056 | 7 | 7 | Carbamidomethylation |
| 451 | Alpha glucosidase | A0A423SJF6\|A0A423SJF6_PENVA | 98369.61 | 275.05826 | 5 | 5 | Carbamidomethylation; Deamidation (NQ) |
| 452 | Ribosomal protein L15 | A0A423TN92\|A0A423TN92_PENVA | 24067.965 | 271.70612 | 5 | 5 | Carbamidomethylation |
| 453 | Small ribosomal subunit protein eS17 | A0A423SX22\|A0A423SX22_PENVA | 15030.457 | 269.8338 | 6 | 6 | Carbamidomethylation |
| 454 | Translationally-controlled tumor protein homolog | A0A3R7SKF0\|A0A3R7SKF0_PENVA | 22810.684 | 269.39044 | 6 | 6 |  |
| 455 | Glycine cleavage system H protein, mitochondrial | A0A423T425\|A0A423T425_PENVA | 10210.336 | 268.86002 | 5 | 5 | Carbamidomethylation; Deamidation (NQ) |
| 456 | Four and a half LIM domains protein 2 | A0A3R7P1C7\|A0A3R7P1C7_PENVA | 63045.074 | 268.31143 | 6 | 6 | Carbamidomethylation |
| 457 | Isoform of A0A423TBL2, Putative proteasome subunit beta type-2-like | A0A3R7M666\|A0A3R7M666_PENVA | 11373.054 | 268.21454 | 5 | 5 | Deamidation (NQ); Oxidation (M) |
| 458 | Proteasome subunit beta | A0A423TBL2\|A0A423TBL2_PENVA | 23263.81 | 239.52023 | 4 | 4 | Deamidation (NQ) |
| 459 | >sp\|K2C1_HUMAN\| | #CONTAM#K2C1_HUMAN\| | 65886.48 | 267.88074 | 6 | 5 |  |
| 460 | Putative phosphate carrier protein, mitochondrial isoform X2 | A0A3R7MZV2\|A0A3R7MZV2_PENVA | 38464.93 | 267.63696 | 6 | 6 | Carbamidomethylation |
| 461 | Putative myosin light chain kinase | A0A423TFG6\|A0A423TFG6_PENVA | 452689.9 | 267.4141 | 11 | 10 | Deamidation (NQ) |
| 462 | Malic enzyme | A0A423T5V2\|A0A423T5V2_PENVA | 70403.86 | 267.16992 | 5 | 5 |  |
| 463 | Trypsinogen 1 | A0A423SXN9\|A0A423SXN9_PENVA | 28258.498 | 260.6436 | 5 | 2 | Carbamidomethylation |
| 464 | Trypsinogen 1 | A0A423TS83\|A0A423TS83_PENVA | 24794.768 | 135.65755 | 2 | 2 |  |
| 465 | Putative dihydropyrimidinase-like isoform X4 | A0A3R7M0S1\|A0A3R7M0S1_PENVA | 61586.168 | 260.4786 | 5 | 5 |  |
| 466 | Polyadenylate-binding protein | A0A3R7QB76\|A0A3R7QB76_PENVA | 66659.24 | 259.5792 | 7 | 7 | Carbamidomethylation; Deamidation (NQ) |
| 467 | Polyadenylate-binding protein | A0A423U4E7\|A0A423U4E7_PENVA | 67462.06 | 259.5792 | 7 | 7 | Carbamidomethylation; Deamidation (NQ) |
| 468 | Peptidyl-prolyl cis-trans isomerase | G5D053\|G5D053_PENVA | 17620.074 | 259.11554 | 5 | 5 |  |
| 469 | Vigilin | A0A3R7PAP7\|A0A3R7PAP7_PENVA | 143706.23 | 258.85828 | 9 | 9 | Acetylation (N-term); Carbamidomethylation; Deamidation (NQ) |
| 470 | Vigilin | A0A3R7PET9\|A0A3R7PET9_PENVA | 74523.88 | 184.8534 | 5 | 5 | Acetylation (N-term); Deamidation (NQ) |
| 471 | Putative vigilin | A0A3R7QIW2\|A0A3R7QIW2_PENVA | 55976.49 | 172.33786 | 3 | 3 |  |
| 472 | Protein hu-li tai shao | A0A423TVY7\|A0A423TVY7_PENVA | 86471.71 | 258.40625 | 5 | 5 | Oxidation (M) |
| 473 | Protein hu-li tai shao | A0A423SNT3\|A0A423SNT3_PENVA | 38412.145 | 218.2496 | 3 | 3 |  |
| 474 | ATP synthase subunit gamma | A0A423TUC1\|A0A423TUC1_PENVA | 35905 | 258.35547 | 6 | 6 | Carbamidomethylation |
| 475 | ATP synthase subunit gamma | A0A3R7PR46\|A0A3R7PR46_PENVA | 36533.527 | 208.2417 | 4 | 4 | Carbamidomethylation |
| 476 | Failed axon connections | A0A3R7SYW9\|A0A3R7SYW9_PENVA | 39564.945 | 258.1112 | 5 | 5 | Carbamidomethylation; Deamidation (NQ) |
| 477 | 14-3-3 epsilon-like transcript variant 1 | A0A3R7LQ50\|A0A3R7LQ50_PENVA | 49705.105 | 257.53253 | 5 | 2 | Carbamidomethylation |
| 478 | Myosin-XVIIIa | A0A423TMF8\|A0A423TMF8_PENVA | 192460.34 | 255.5018 | 5 | 5 | Acetylation (N-term) |
| 479 | isocitrate dehydrogenase (NADP(+)) | A0A3R7M382\|A0A3R7M382_PENVA | 35560.582 | 255.23242 | 5 | 4 | Carbamidomethylation |
| 480 | Proteasome subunit alpha type | A0A423SM29\|A0A423SM29_PENVA | 29059.2 | 255.21008 | 5 | 5 | Carbamidomethylation |
| 481 | Heat shock protein | A0A423TIS3\|A0A423TIS3_PENVA | 21025.73 | 254.06801 | 4 | 4 |  |
| 482 | SHSP domain-containing protein | A0A3R7QA51\|A0A3R7QA51_PENVA | 68244.77 | 254.05531 | 4 | 3 | Deamidation (NQ) |
| 483 | Serine hydroxymethyltransferase | A0A423SJP6\|A0A423SJP6_PENVA | 53112.566 | 253.86368 | 5 | 5 |  |
| 484 | Serine hydroxymethyltransferase | A0A3R7QFR9\|A0A3R7QFR9_PENVA | 87497.836 | 150.70627 | 2 | 2 |  |
| 485 | Trypsin | A0A3R7M720\|A0A3R7M720_PENVA | 29434.29 | 253.72859 | 4 | 1 | Carbamidomethylation |
| 486 | Trypsinogen 1 | A0A3R7LZM0\|A0A3R7LZM0_PENVA | 35714.727 | 119.42483 | 1 | 1 |  |
| 487 | Uncharacterized protein | A0A423SQX9\|A0A423SQX9_PENVA | 42194.367 | 253.46353 | 4 | 4 |  |
| 488 | Heat shock protein | A0A3R7MCL9\|A0A3R7MCL9_PENVA | 58756.938 | 253.0886 | 5 | 5 | Carbamidomethylation |
| 489 | Cathepsin l | A0A3R7ST81\|A0A3R7ST81_PENVA | 35685.746 | 252.69464 | 4 | 4 | Carbamidomethylation |
| 490 | Cathepsin l | A0A423TC40\|A0A423TC40_PENVA | 47462.125 | 252.69464 | 4 | 4 | Carbamidomethylation |
| 491 | Proteasome subunit alpha type | A0A3R7QBK5\|A0A3R7QBK5_PENVA | 24173.775 | 252.34215 | 5 | 5 |  |
| 492 | Proteasome subunit alpha type | A0A423TAV2\|A0A423TAV2_PENVA | 20644.18 | 133.73587 | 2 | 2 |  |
| 493 | Proteasome subunit beta | A0A3R7MJS2\|A0A3R7MJS2_PENVA | 30883.324 | 252.25441 | 5 | 5 | Carbamidomethylation |
| 494 | Dihydrolipoyllysine-residue succinyltransferase component of 2-oxoglutarate dehydrogenase complex, mitochondrial | A0A3R7MC33\|A0A3R7MC33_PENVA | 46549.668 | 252.16476 | 5 | 5 | Acetylation (N-term); Carbamidomethylation; Deamidation (NQ) |
| 495 | Projectin | A0A3R7SRE5\|A0A3R7SRE5_PENVA | 48966.035 | 251.90643 | 4 | 4 | Oxidation (M) |
| 496 | Tail muscle elongation factor 1 gamma | A0A3R7NDA8\|A0A3R7NDA8_PENVA | 48528.406 | 251.58333 | 4 | 4 |  |
| 497 | Putative aminopeptidase W07G4.4 | A0A3R7M2D5\|A0A3R7M2D5_PENVA | 45986.25 | 250.3425 | 4 | 4 |  |
| 498 | Protein kinase C and casein kinase substrate in neurons protein 2 | A0A3R7M5X2\|A0A3R7M5X2_PENVA | 50000.754 | 249.8988 | 4 | 4 | Carbamidomethylation |
| 499 | Aspartate aminotransferase | A0A423U3M3\|A0A423U3M3_PENVA | 45511.13 | 249.62708 | 4 | 4 | Carbamidomethylation |
| 500 | MYND-type domain-containing protein | A0A3R7PK09\|A0A3R7PK09_PENVA | 68077.36 | 246.12639 | 4 | 4 |  |
| 501 | Uncharacterized protein | A0A423SU64\|A0A423SU64_PENVA | 154581.89 | 121.29056 | 1 | 1 |  |
| 502 | Calpain M | A0A423SQ82\|A0A423SQ82_PENVA | 59876.35 | 245.15686 | 4 | 4 |  |
| 503 | Muscle-specific calpain | A0A423SQ73\|A0A423SQ73_PENVA | 39141.117 | 177.68967 | 2 | 2 |  |
| 504 | Peptidase S1 and S6 chymotrypsin/Hap | A0A3R7QBX6\|A0A3R7QBX6_PENVA | 38076.684 | 244.8026 | 4 | 4 | Carbamidomethylation |
| 505 | Beta-1,3-glucan-binding protein | A0A423T231\|A0A423T231_PENVA | 49565.258 | 243.58092 | 4 | 3 |  |
| 506 | I-connectin | A0A3R7MNV4\|A0A3R7MNV4_PENVA | 54633.984 | 243.50098 | 5 | 3 |  |
| 507 | Thioredoxin | A0A3R7LRU8\|A0A3R7LRU8_PENVA | 11947.737 | 243.39703 | 5 | 5 |  |
| 508 | TATA-binding protein interacting (TIP20) domain-containing protein | A0A423SHU4\|A0A423SHU4_PENVA | 124307.57 | 242.11494 | 5 | 5 | Carbamidomethylation; Deamidation (NQ) |
| 509 | Hydroxyacylglutathione hydrolase, mitochondrial | A0A423SZK6\|A0A423SZK6_PENVA | 34064.7 | 241.19157 | 4 | 4 | Carbamidomethylation |
| 510 | phosphorylase kinase | A0A3R7M7J0\|A0A3R7M7J0_PENVA | 44585.71 | 241.18698 | 4 | 4 |  |
| 511 | TAR DNA-binding protein 43 | A0A423TX11\|A0A423TX11_PENVA | 46919.33 | 238.94138 | 5 | 5 | Carbamidomethylation |
| 512 | Putative Heterogeneous nuclear ribonucleoprotein A1 | A0A3R7PHF6\|A0A3R7PHF6_PENVA | 20652.133 | 223.14487 | 3 | 3 | Carbamidomethylation |
| 513 | Putative F-box/LRR-repeat protein 7 isoform X3 | A0A423TL36\|A0A423TL36_PENVA | 52229.703 | 238.74857 | 4 | 4 |  |
| 514 | Peptidyl-prolyl cis-trans isomerase E | A0A3R7N945\|A0A3R7N945_PENVA | 32155.178 | 238.2094 | 5 | 5 | Deamidation (NQ) |
| 515 | Small ribosomal subunit protein eS6 | A0A3R7N4Z8\|A0A3R7N4Z8_PENVA | 37797.246 | 237.70827 | 4 | 4 |  |
| 516 | 40S ribosomal protein S21 | A0A3R7SSF0\|A0A3R7SSF0_PENVA | 9340.572 | 237.43019 | 4 | 4 | Acetylation (N-term) |
| 517 | Fasciclin-2 | A0A3R7QIP8\|A0A3R7QIP8_PENVA | 93680.375 | 236.89162 | 4 | 4 | Carbamidomethylation |
| 518 | Putative 40S ribosomal protein S3-like isoform X1 | A0A423SFL3\|A0A423SFL3_PENVA | 29563.447 | 236.7996 | 4 | 4 |  |
| 519 | Myosin light chain | A0A3R7P8N3\|A0A3R7P8N3_PENVA | 16461.145 | 236.07953 | 4 | 4 |  |
| 520 | Beta-1,3-glucan binding protein | A0A423TFV7\|A0A423TFV7_PENVA | 44712.926 | 235.40907 | 4 | 4 |  |
| 521 | Lipopolysaccharide and beta-1,3-glucan binding protein | A0A423TFI6\|A0A423TFI6_PENVA | 24341.94 | 95.46179 | 1 | 1 |  |
| 522 | ADP-ribosylation factor 1 | A0A3R7MW78\|A0A3R7MW78_PENVA | 28164.46 | 235.38713 | 4 | 4 |  |
| 523 | ADP-ribosylation factor | A0A423TXF7\|A0A423TXF7_PENVA | 32838.613 | 164.62933 | 2 | 2 |  |
| 524 | ADP-ribosylation factor | A0A3R7P8U4\|A0A3R7P8U4_PENVA | 39712.277 | 92.60784 | 1 | 1 |  |
| 525 | ADP-ribosylation factor | A0A423SIV0\|A0A423SIV0_PENVA | 37744.543 | 92.60784 | 1 | 1 |  |
| 526 | Putative PDZ and LIM domain protein Zasp | A0A423TZI4\|A0A423TZI4_PENVA | 77655.98 | 232.69284 | 5 | 5 | Deamidation (NQ); Oxidation (M) |
| 527 | Histone H1 | A0A3R7PSD9\|A0A3R7PSD9_PENVA | 32649.18 | 231.82445 | 4 | 4 | Oxidation (M) |
| 528 | T-complex protein 1 subunit theta | A0A3R7QPD2\|A0A3R7QPD2_PENVA | 59511.336 | 231.3734 | 4 | 4 | Carbamidomethylation |
| 529 | Histone H2B | A0A3R7M7L1\|A0A3R7M7L1_PENVA | 18643.773 | 230.96555 | 5 | 5 | Deamidation (NQ) |
| 530 | Histone H2B | A0A3R7PKU8\|A0A3R7PKU8_PENVA | 38142.19 | 230.96555 | 5 | 5 | Deamidation (NQ) |
| 531 | Histone H2B | A0A3R7M767\|A0A3R7M767_PENVA | 12996.1045 | 230.96555 | 5 | 5 | Deamidation (NQ) |
| 532 | Isoform of A0A3R7PKU8, Histone H2B | A0A3R7PJW9\|A0A3R7PJW9_PENVA | 13668.919 | 230.96555 | 5 | 5 | Deamidation (NQ) |
| 533 | Histone H2B | A0A3R7MSX5\|A0A3R7MSX5_PENVA | 9732.303 | 230.96555 | 5 | 5 | Deamidation (NQ) |
| 534 | Isoform of A0A3R7PKU8, Putative H2B histone | A0A3R7MSV8\|A0A3R7MSV8_PENVA | 11457.208 | 195.83914 | 3 | 3 | Deamidation (NQ) |
| 535 | Histone H2B.1/H2B.2 | A0A3R7M298\|A0A3R7M298_PENVA | 16090.859 | 195.83914 | 3 | 3 | Deamidation (NQ) |
| 536 | Putative H2B histone | A0A3R7PJX6\|A0A3R7PJX6_PENVA | 16280.872 | 195.83914 | 3 | 3 | Deamidation (NQ) |
| 537 | Voltage-dependent anion-selective channel | A0A3R7T1P2\|A0A3R7T1P2_PENVA | 28390.043 | 230.17226 | 4 | 4 |  |
| 538 | Voltage-dependent anion-selective channel | A0A423TL51\|A0A423TL51_PENVA | 29987.877 | 230.17226 | 4 | 4 |  |
| 539 | Uncharacterized protein | A0A423TDP7\|A0A423TDP7_PENVA | 78013.67 | 229.55856 | 4 | 4 | Acetylation (N-term) |
| 540 | Uncharacterized protein | A0A423SME7\|A0A423SME7_PENVA | 47791.566 | 47.226437 | 1 | 1 | Acetylation (N-term) |
| 541 | Putative zinc finger protein OZF | A0A3R7T1J7\|A0A3R7T1J7_PENVA | 60579.367 | 47.226437 | 1 | 1 | Acetylation (N-term) |
| 542 | Histone H4 | A0A3R7Q5C5\|A0A3R7Q5C5_PENVA | 29108.162 | 229.31807 | 4 | 4 |  |
| 543 | 60S ribosomal protein L4 C-terminal domain-containing protein | A0A3R7SZH4\|A0A3R7SZH4_PENVA | 45942.867 | 227.91519 | 4 | 4 | Carbamidomethylation |
| 544 | F-actin-capping protein subunit alpha | A0A3R7QCH9\|A0A3R7QCH9_PENVA | 32437.188 | 227.89212 | 4 | 4 | Acetylation (N-term); Carbamidomethylation |
| 545 | Putative hemocyte protein-glutamine gamma-glutamyltransferase | A0A3R7PCW4\|A0A3R7PCW4_PENVA | 85399.49 | 227.53868 | 4 | 3 | Carbamidomethylation |
| 546 | Putative CAP-Gly domain-containing linker protein 1 isoform X2 | A0A3R7SKL6\|A0A3R7SKL6_PENVA | 82727.14 | 225.00809 | 4 | 4 |  |
| 547 | RNA-binding protein 1 | A0A3R7Q212\|A0A3R7Q212_PENVA | 17758.33 | 224.99806 | 3 | 3 |  |
| 548 | Putative RNA-binding protein 1-like isoform X4 (Fragment) | A0A3R7M3S4\|A0A3R7M3S4_PENVA | 15165.152 | 224.99806 | 3 | 3 |  |
| 549 | Ribosomal protein | A0A3R7PEP7\|A0A3R7PEP7_PENVA | 29279.35 | 224.87764 | 3 | 3 | Carbamidomethylation; Deamidation (NQ) |
| 550 | Junctophilin-2 | A0A423SPH2\|A0A423SPH2_PENVA | 18841.5 | 224.43507 | 3 | 3 | Carbamidomethylation |
| 551 | Sorbin and SH3 domain-containing protein | A0A423TN49\|A0A423TN49_PENVA | 55952.85 | 224.23161 | 3 | 3 | Carbamidomethylation; Deamidation (NQ) |
| 552 | Aspartate aminotransferase | A0A3R7MVJ7\|A0A3R7MVJ7_PENVA | 47025.043 | 222.81035 | 3 | 3 | Carbamidomethylation; Deamidation (NQ) |
| 553 | ribonuclease H | A0A423U9M1\|A0A423U9M1_PENVA | 24305.283 | 222.56311 | 3 | 3 |  |
| 554 | Heat shock protein 21 | A0A423TTX7\|A0A423TTX7_PENVA | 44001.098 | 221.61707 | 4 | 4 |  |
| 555 | Uncharacterized protein | A0A3R7NTQ8\|A0A3R7NTQ8_PENVA | 11836.617 | 220.76111 | 3 | 3 |  |
| 556 | NSFL1 cofactor p47 | A0A423T2L2\|A0A423T2L2_PENVA | 41560.066 | 220.52489 | 3 | 3 |  |
| 557 | SEP domain-containing protein | A0A3R7PSZ3\|A0A3R7PSZ3_PENVA | 16695.555 | 179.54082 | 2 | 2 |  |
| 558 | Putative heterogeneous nuclear ribonucleoprotein H2 isoform X2 | A0A423SN10\|A0A423SN10_PENVA | 43839.805 | 220.43964 | 3 | 3 |  |
| 559 | Uncharacterized protein | A0A3R7QFQ3\|A0A3R7QFQ3_PENVA | 15539.58 | 220.25989 | 3 | 3 | Carbamidomethylation |
| 560 | Ribosome maturation protein SBDS | A0A3R7QXH6\|A0A3R7QXH6_PENVA | 33643.094 | 220.02983 | 4 | 4 | Carbamidomethylation |
| 561 | Crustacyanin subunit C | A0A3R7QBN0\|A0A3R7QBN0_PENVA | 22512.314 | 219.6913 | 5 | 3 | Deamidation (NQ) |
| 562 | Crustacyanin subunit C | A0A3R7LTS1\|A0A3R7LTS1_PENVA | 26981.547 | 219.6913 | 5 | 3 | Deamidation (NQ) |
| 563 | Crustacyanin subunit C | A0A423SEP0\|A0A423SEP0_PENVA | 20900.69 | 219.6913 | 5 | 3 | Deamidation (NQ) |
| 564 | Crustacyanin subunit C | A0A3R7LRS6\|A0A3R7LRS6_PENVA | 19109.494 | 207.90703 | 4 | 3 | Deamidation (NQ) |
| 565 | Crustacyanin subunit C | A0A423SER3\|A0A423SER3_PENVA | 22539.344 | 206.52696 | 4 | 3 |  |
| 566 | Crustacyanin subunit C | A0A3R7SIP5\|A0A3R7SIP5_PENVA | 22463.334 | 206.52696 | 4 | 3 |  |
| 567 | Crustacyanin subunit C | A0A3R7PXN3\|A0A3R7PXN3_PENVA | 14771.801 | 191.79662 | 3 | 3 |  |
| 568 | Crustacyanin subunit C | A0A423U583\|A0A423U583_PENVA | 22189.254 | 111.974884 | 2 | 1 |  |
| 569 | Crustacyanin subunit C | A0A423U591\|A0A423U591_PENVA | 22134.098 | 111.974884 | 2 | 1 |  |
| 570 | Crustacyanin subunit C | A0A3R7QYU1\|A0A3R7QYU1_PENVA | 21262.094 | 111.974884 | 2 | 1 |  |
| 571 | Crustacyanin subunit C | A0A423U575\|A0A423U575_PENVA | 21512.346 | 111.974884 | 2 | 1 |  |
| 572 | Crustacyanin subunit C | A0A3R7PVI2\|A0A3R7PVI2_PENVA | 22093.002 | 111.974884 | 2 | 1 |  |
| 573 | Crustacyanin subunit C | A0A423U563\|A0A423U563_PENVA | 17728.129 | 111.974884 | 2 | 1 |  |
| 574 | Crustacyanin subunit C | A0A423U543\|A0A423U543_PENVA | 22164.125 | 111.974884 | 2 | 1 |  |
| 575 | Crustacyanin-A1 subunit | A0A3R7MR94\|A0A3R7MR94_PENVA | 16620.672 | 82.5142 | 1 | 1 |  |
| 576 | Crustacyanin-C1 subunit | A0A423U564\|A0A423U564_PENVA | 11018.293 | 82.5142 | 1 | 1 |  |
| 577 | Crustacyanin subunit C | A0A3R7M141\|A0A3R7M141_PENVA | 20515.38 | 82.5142 | 1 | 1 |  |
| 578 | Crustacyanin-C1 subunit | A0A423U544\|A0A423U544_PENVA | 15699.485 | 82.5142 | 1 | 1 |  |
| 579 | Crustacyanin subunit C | A0A423U516\|A0A423U516_PENVA | 9951.173 | 82.5142 | 1 | 1 |  |
| 580 | Crustacyanin subunit C | A0A3R7NNS7\|A0A3R7NNS7_PENVA | 14426.967 | 82.5142 | 1 | 1 |  |
| 581 | Crustacyanin subunit C | A0A3R7PVH5\|A0A3R7PVH5_PENVA | 16565.686 | 82.5142 | 1 | 1 |  |
| 582 | Crustacyanin-C1 subunit | A0A423U599\|A0A423U599_PENVA | 13753.292 | 82.5142 | 1 | 1 |  |
| 583 | Crustacyanin-C1 subunit | A0A423U556\|A0A423U556_PENVA | 15823.709 | 82.5142 | 1 | 1 |  |
| 584 | Crustacyanin subunit C | A0A423U505\|A0A423U505_PENVA | 17990.348 | 82.5142 | 1 | 1 |  |
| 585 | Large ribosomal subunit protein uL2 | Q2I3E8\|Q2I3E8_PENVA | 28233.857 | 219.08119 | 3 | 3 | Carbamidomethylation |
| 586 | Large ribosomal subunit protein uL2 | A0A3R7QRC2\|A0A3R7QRC2_PENVA | 19352.346 | 175.21106 | 2 | 2 | Carbamidomethylation |
| 587 | Putative tropomodulin isoform X2 | A0A423TNY6\|A0A423TNY6_PENVA | 16279.407 | 218.89584 | 3 | 3 |  |
| 588 | Citrate synthase | A0A423SYM7\|A0A423SYM7_PENVA | 52056.69 | 218.68027 | 3 | 3 |  |
| 589 | Putative rho GDP-dissociation inhibitor 2 (Fragment) | A0A3R7NUJ0\|A0A3R7NUJ0_PENVA | 23908.984 | 218.65703 | 3 | 3 | Acetylation (N-term) |
| 590 | Putative rho GDP-dissociation inhibitor 2 isoform X1 | A0A423SB23\|A0A423SB23_PENVA | 19749.58 | 103.29932 | 1 | 1 |  |
| 591 | Putative serine proteinase inhibitor | A0A3R7QNE7\|A0A3R7QNE7_PENVA | 40458.656 | 218.60742 | 3 | 3 |  |
| 592 | Isoform of A0A423SUV4, ATP synthase subunit beta | A0A3R7SN35\|A0A3R7SN35_PENVA | 36103.02 | 218.42433 | 5 | 2 |  |
| 593 | Ribosomal protein S7 domain-containing protein | A0A423U2Y2\|A0A423U2Y2_PENVA | 23507.072 | 216.36192 | 3 | 3 | Carbamidomethylation |
| 594 | Putative 40S ribosomal protein S5 isoform X2 | A0A423U2R2\|A0A423U2R2_PENVA | 19919.203 | 176.45335 | 2 | 2 | Carbamidomethylation |
| 595 | KASH domain-containing protein | A0A423TWS3\|A0A423TWS3_PENVA | 1328300.8 | 215.91257 | 5 | 5 |  |
| 596 | Tetraspanin | A0A0P0C4Q7\|A0A0P0C4Q7_PENVA | 25728.951 | 215.78175 | 3 | 3 | Carbamidomethylation; Deamidation (NQ) |
| 597 | T-complex protein 1 subunit epsilon | A0A3R7P4W6\|A0A3R7P4W6_PENVA | 58996.44 | 215.237 | 4 | 4 | Acetylation (N-term); Carbamidomethylation; Oxidation (M) |
| 598 | GTP-binding nuclear protein | A0A3R7SGV1\|A0A3R7SGV1_PENVA | 29658.148 | 213.9647 | 4 | 4 | Carbamidomethylation |
| 599 | Proteasome subunit alpha type | A0A423T969\|A0A423T969_PENVA | 26067.94 | 213.79492 | 3 | 3 |  |
| 600 | 60S acidic ribosomal protein P0 | A0A3R7T0I1\|A0A3R7T0I1_PENVA | 34174.258 | 212.9135 | 4 | 4 | Carbamidomethylation |
| 601 | small monomeric GTPase (Fragment) | A0A3R7P9I3\|A0A3R7P9I3_PENVA | 19492.236 | 211.7616 | 4 | 3 |  |
| 602 | small monomeric GTPase | A0A3R7M6R9\|A0A3R7M6R9_PENVA | 20890.984 | 211.7616 | 4 | 3 |  |
| 603 | Uncharacterized protein | A0A423SZE7\|A0A423SZE7_PENVA | 22008.994 | 211.45915 | 3 | 3 |  |
| 604 | Ribosomal protein L7 | K4KFY7\|K4KFY7_PENVA | 32423.492 | 210.6812 | 3 | 3 | Carbamidomethylation |
| 605 | Ribosomal protein L7 | A0A3R7N2L2\|A0A3R7N2L2_PENVA | 32272.36 | 166.45697 | 2 | 2 |  |
| 606 | Aldose 1-epimerase | A0A3R7MME0\|A0A3R7MME0_PENVA | 38982.734 | 210.11655 | 3 | 3 | Carbamidomethylation |
| 607 | Putative filamin-A isoform X3 | A0A423U897\|A0A423U897_PENVA | 110516.28 | 210.05948 | 3 | 3 |  |
| 608 | Ribosomal protein L11 | A0A423UAP1\|A0A423UAP1_PENVA | 20342.625 | 209.96999 | 4 | 4 |  |
| 609 | N-acyl-L-amino-acid amidohydrolase | A0A423T1E2\|A0A423T1E2_PENVA | 55076.11 | 208.85852 | 3 | 3 | Carbamidomethylation |
| 610 | Dihydropteridine reductase | A0A3R7MB03\|A0A3R7MB03_PENVA | 24659.168 | 207.60666 | 3 | 3 |  |
| 611 | Crustacyanin subunit C | A0A423SEN6\|A0A423SEN6_PENVA | 22093.97 | 207.40971 | 3 | 1 |  |
| 612 | Uncharacterized protein | A0A423SR08\|A0A423SR08_PENVA | 16239.294 | 206.51639 | 3 | 3 |  |
| 613 | Putative plasminogen activator inhibitor 1 RNA-binding protein isoform X1 | A0A3R7QCB8\|A0A3R7QCB8_PENVA | 19148.574 | 203.76442 | 4 | 4 | Deamidation (NQ) |
| 614 | Putative plasminogen activator inhibitor 1 RNA-binding protein-like isoform X4 | A0A3R7PPQ9\|A0A3R7PPQ9_PENVA | 26459.955 | 203.76442 | 4 | 4 | Deamidation (NQ) |
| 615 | Cell division control protein 42 homolog | A0A423TIL7\|A0A423TIL7_PENVA | 21397.678 | 202.77116 | 3 | 3 |  |
| 616 | Glycogen debranching enzyme | A0A423T9T8\|A0A423T9T8_PENVA | 78442.08 | 202.31606 | 3 | 3 | Carbamidomethylation |
| 617 | >sp\|K1C10_HUMAN\| | #CONTAM#K1C10_HUMAN\| | 59518.656 | 202.18463 | 3 | 3 |  |
| 618 | >sp\|K1C15_SHEEP\| | #CONTAM#K1C15_SHEEP\| | 48770.03 | 102.35205 | 1 | 1 |  |
| 619 | Putative muscle-specific protein 20 | A0A423TDK5\|A0A423TDK5_PENVA | 25208.715 | 200.29655 | 3 | 3 |  |
| 620 | Large ribosomal subunit protein uL6 | A0A3R7M1R2\|A0A3R7M1R2_PENVA | 21820.402 | 199.49957 | 3 | 3 |  |
| 621 | Small ribosomal subunit protein eS1 | A0A3R7MJX1\|A0A3R7MJX1_PENVA | 30053.754 | 199.33328 | 3 | 3 |  |
| 622 | ATP synthase subunit b | A0A423T934\|A0A423T934_PENVA | 28215.467 | 199.06688 | 3 | 3 |  |
| 623 | Aquaporin | A0A3R7PEU6\|A0A3R7PEU6_PENVA | 33973.66 | 199.0228 | 5 | 5 | Carbamidomethylation |
| 624 | Small nuclear ribonucleoprotein Sm D1 | A0A3R7MBV4\|A0A3R7MBV4_PENVA | 10665.268 | 198.57669 | 3 | 3 |  |
| 625 | Hydroxyacyl-coenzyme A dehydrogenase, mitochondrial | A0A3R7QAN5\|A0A3R7QAN5_PENVA | 33629.76 | 194.99937 | 3 | 3 |  |
| 626 | Proteasome subunit beta | A0A423SPQ8\|A0A423SPQ8_PENVA | 27169.86 | 194.9196 | 3 | 3 | Carbamidomethylation |
| 627 | Ryanodine receptor | A0A3R7MMB5\|A0A3R7MMB5_PENVA | 38271.81 | 193.74149 | 3 | 3 |  |
| 628 | >sp\|TRY1_BOVIN\| | #CONTAM#TRY1_BOVIN\| | 25424.768 | 193.19965 | 3 | 2 | Acetylation (N-term); Carbamidomethylation; Deamidation (NQ) |
| 629 | Kinesin-like protein | A0A423TSE4\|A0A423TSE4_PENVA | 108938.84 | 191.82477 | 3 | 3 | Deamidation (NQ) |
| 630 | Putative hfb2 protein | A0A3R7PAX5\|A0A3R7PAX5_PENVA | 54279.547 | 190.4553 | 3 | 3 |  |
| 631 | Dual specificity protein phosphatase | A0A3R7NPA3\|A0A3R7NPA3_PENVA | 21857.2 | 187.9456 | 3 | 3 | Deamidation (NQ) |
| 632 | Eukaryotic translation initiation factor 3 subunit G | A0A3R7QN85\|A0A3R7QN85_PENVA | 32946 | 187.19324 | 3 | 3 |  |
| 633 | Putative serine/threonine-protein phosphatase PP1-beta catalytic subunit-like | A0A3R7P657\|A0A3R7P657_PENVA | 14778.639 | 186.57953 | 3 | 1 | Acetylation (N-term) |
| 634 | Metalloendopeptidase | A0A3R7P5D9\|A0A3R7P5D9_PENVA | 28871.48 | 186.00471 | 3 | 3 |  |
| 635 | Putative cysteine proteinase (Fragment) | A0A3R7QHX6\|A0A3R7QHX6_PENVA | 104051.46 | 185.20966 | 2 | 2 | Carbamidomethylation |
| 636 | Putative multiple epidermal growth factor-like domains protein 6 isoform X6 | A0A3R7NFP6\|A0A3R7NFP6_PENVA | 107998.09 | 185.1491 | 2 | 2 | Carbamidomethylation |
| 637 | Myotrophin | A0A423TKX6\|A0A423TKX6_PENVA | 12385.011 | 184.4538 | 2 | 2 |  |
| 638 | Basement membrane-specific heparan sulfate proteoglycan core protein | A0A3R7MHL2\|A0A3R7MHL2_PENVA | 49245.574 | 184.23871 | 2 | 2 | Carbamidomethylation |
| 639 | Myosin essential light chain | A0A3R7PSX2\|A0A3R7PSX2_PENVA | 8882.774 | 184.1173 | 2 | 2 |  |
| 640 | Myosin essential light chain | A0A3R7N886\|A0A3R7N886_PENVA | 11580.969 | 121.42716 | 1 | 1 |  |
| 641 | Alpha-galactosidase | A0A423SKV4\|A0A423SKV4_PENVA | 50289.023 | 183.4173 | 2 | 2 |  |
| 642 | Cathepsin L | A0A423SD78\|A0A423SD78_PENVA | 43081.133 | 183.21178 | 2 | 2 |  |
| 643 | phospholipase D | A0A3R7SLU0\|A0A3R7SLU0_PENVA | 93579.62 | 183.16603 | 2 | 2 |  |
| 644 | Outer dense fiber protein 3 | A0A3R7PNZ6\|A0A3R7PNZ6_PENVA | 17171.424 | 183.04637 | 2 | 2 |  |
| 645 | BTB domain-containing protein | A0A3R7T196\|A0A3R7T196_PENVA | 68741.2 | 182.36401 | 2 | 2 |  |
| 646 | Peritrophin-44-like protein | A0A3R7P1R8\|A0A3R7P1R8_PENVA | 23806.814 | 182.19945 | 2 | 2 | Carbamidomethylation |
| 647 | Ferritin | A0A423SK61\|A0A423SK61_PENVA | 19420.86 | 182.06528 | 2 | 2 |  |
| 648 | Isoform of A0A3R7MMB5, Inositol 1,4,5-trisphosphate/ryanodine receptor domain-containing protein | A0A423U9W7\|A0A423U9W7_PENVA | 28911.803 | 181.90057 | 2 | 2 |  |
| 649 | Sodium potassium-transporting ATPase subunit beta | A0A3R7M5U5\|A0A3R7M5U5_PENVA | 38128.36 | 180.90648 | 4 | 4 | Deamidation (NQ) |
| 650 | Sodium potassium-transporting ATPase subunit beta | A0A3R7PIA5\|A0A3R7PIA5_PENVA | 23945.36 | 91.76773 | 2 | 2 | Deamidation (NQ) |
| 651 | palmitoyl-protein hydrolase | A0A423SHU2\|A0A423SHU2_PENVA | 25947.549 | 180.15259 | 2 | 2 |  |
| 652 | serine--tRNA ligase | A0A423U6I5\|A0A423U6I5_PENVA | 66620.49 | 180.13487 | 2 | 2 |  |
| 653 | Anoctamin | A0A423TAV3\|A0A423TAV3_PENVA | 76946.04 | 180.0874 | 2 | 2 |  |
| 654 | QM protein | A0A3R7QCC2\|A0A3R7QCC2_PENVA | 41404.805 | 179.79575 | 2 | 2 | Carbamidomethylation |
| 655 | Inositol-1-monophosphatase | A0A3R7M590\|A0A3R7M590_PENVA | 27010.004 | 178.79822 | 2 | 2 |  |
| 656 | 40S ribosomal protein S7 | A0A3R7MSS7\|A0A3R7MSS7_PENVA | 27129.69 | 177.63864 | 2 | 2 |  |
| 657 | Lamin-B receptor of TUDOR domain-containing protein | A0A3R7QHT8\|A0A3R7QHT8_PENVA | 67481.29 | 177.44566 | 3 | 2 |  |
| 658 | phospholipase D | A0A3R7PIK3\|A0A3R7PIK3_PENVA | 106690.2 | 177.07854 | 3 | 3 | Deamidation (NQ) |
| 659 | Proteasome subunit beta | A0A3R7P4C8\|A0A3R7P4C8_PENVA | 22510.738 | 176.52484 | 3 | 3 | Deamidation (NQ) |
| 660 | RRM domain-containing protein | A0A3R7SSF9\|A0A3R7SSF9_PENVA | 39443.48 | 176.06061 | 2 | 2 | Acetylation (N-term); Deamidation (NQ) |
| 661 | S10e ribosomal protein | A0A423TWT2\|A0A423TWT2_PENVA | 17621.24 | 176.03516 | 2 | 2 | Carbamidomethylation |
| 662 | Cdc42-like protein (Fragment) | A0A3R7PNH5\|A0A3R7PNH5_PENVA | 6812.642 | 175.33276 | 2 | 1 |  |
| 663 | Putative ensconsin-like isoform X8 | A0A423U5T5\|A0A423U5T5_PENVA | 120392.16 | 175.2238 | 2 | 2 |  |
| 664 | Heme-binding protein 2 | A0A3R7QRR3\|A0A3R7QRR3_PENVA | 29704.781 | 174.79248 | 2 | 2 | Oxidation (M) |
| 665 | Ribosomal protein L3 | A0A3R7PW55\|A0A3R7PW55_PENVA | 46413.64 | 174.3056 | 2 | 2 |  |
| 666 | Uncharacterized protein | A0A3R7PQK0\|A0A3R7PQK0_PENVA | 45176.23 | 173.37007 | 2 | 2 |  |
| 667 | Large ribosomal subunit protein eL6 | A0A423SBM6\|A0A423SBM6_PENVA | 52782.496 | 173.12784 | 2 | 2 | Deamidation (NQ) |
| 668 | Large ribosomal subunit protein eL6 | A0A423SBQ2\|A0A423SBQ2_PENVA | 35834.85 | 173.12784 | 2 | 2 | Deamidation (NQ) |
| 669 | Cytochrome c oxidase subunit 5A, mitochondrial | A0A3R7Q0U8\|A0A3R7Q0U8_PENVA | 17140.53 | 173.09702 | 2 | 2 |  |
| 670 | Putative glutamyl-tRNA synthetase, cytoplasmic | A0A3R7P5C3\|A0A3R7P5C3_PENVA | 178177.25 | 172.71623 | 2 | 2 |  |
| 671 | Tubulin-specific chaperone A | A0A3R7PPY4\|A0A3R7PPY4_PENVA | 12910.643 | 171.69589 | 3 | 3 |  |
| 672 | >sp\|K1C9_HUMAN\| | #CONTAM#K1C9_HUMAN\| | 62129.438 | 171.65688 | 2 | 2 |  |
| 673 | Histone H2A | A0A423SG35\|A0A423SG35_PENVA | 13447.642 | 170.1031 | 4 | 1 |  |
| 674 | Putative malate dehydrogenase, cytoplasmic isoform X2 | A0A423SLW8\|A0A423SLW8_PENVA | 24833.297 | 167.67888 | 3 | 3 | Carbamidomethylation; Deamidation (NQ) |
| 675 | Mesencephalic astrocyte-derived neurotrophic factor homolog | A0A423TIF1\|A0A423TIF1_PENVA | 32154.65 | 167.13486 | 2 | 2 | Carbamidomethylation |
| 676 | Putative E1b-55kD-associated protein | A0A3R7SMF4\|A0A3R7SMF4_PENVA | 110125.57 | 166.67293 | 2 | 2 |  |
| 677 | Zasp-like motif domain-containing protein | A0A3R7PWP5\|A0A3R7PWP5_PENVA | 13835.487 | 166.61292 | 3 | 3 |  |
| 678 | Uncharacterized protein | A0A3R7PPZ0\|A0A3R7PPZ0_PENVA | 14463.537 | 164.73816 | 2 | 2 |  |
| 679 | I-connectin | A0A3R7PZH7\|A0A3R7PZH7_PENVA | 89586.61 | 164.22412 | 3 | 1 |  |
| 680 | Uncharacterized protein | A0A3R7PAK4\|A0A3R7PAK4_PENVA | 107929.52 | 163.72926 | 2 | 2 |  |
| 681 | N-acyl-aliphatic-L-amino acid amidohydrolase | A0A3R7SS95\|A0A3R7SS95_PENVA | 45352.2 | 163.44838 | 2 | 2 |  |
| 682 | Isoform of A0A3R7SS95, Putative aminoacylase-1 | A0A423TPF6\|A0A423TPF6_PENVA | 27504.676 | 121.48797 | 1 | 1 |  |
| 683 | Putative junctophilin-3-like | A0A423TSY0\|A0A423TSY0_PENVA | 40430.816 | 161.83987 | 3 | 3 | Deamidation (NQ) |
| 684 | Transglutaminase-like domain-containing protein | A0A3R7SJE1\|A0A3R7SJE1_PENVA | 53133.016 | 160.11276 | 2 | 1 | Carbamidomethylation |
| 685 | F-actin-capping protein subunit beta | A0A3R7MIL4\|A0A3R7MIL4_PENVA | 31189.148 | 159.05197 | 2 | 2 | Carbamidomethylation; Deamidation (NQ) |
| 686 | Putative biorientation of chromosomes in cell division protein 1-like 1 isoform X4 | A0A423TYP1\|A0A423TYP1_PENVA | 68922.664 | 158.90715 | 2 | 2 |  |
| 687 | Phospholipase-like protein A2, group | A0A423SUK8\|A0A423SUK8_PENVA | 24550.352 | 156.25763 | 2 | 2 |  |
| 688 | Crustacyanin subunit A | A0A3R7LYW0\|A0A3R7LYW0_PENVA | 21038.7 | 154.79013 | 3 | 1 | Carbamidomethylation; Deamidation (NQ) |
| 689 | Crustacyanin subunit A | A0A423SEN2\|A0A423SEN2_PENVA | 21377.021 | 154.79013 | 3 | 1 | Carbamidomethylation; Deamidation (NQ) |
| 690 | Metallo-beta-lactamase domain-containing protein | A0A3R7QRL1\|A0A3R7QRL1_PENVA | 31484.055 | 154.0664 | 2 | 2 | Carbamidomethylation |
| 691 | Putative surface protein bspA-like (Fragment) | A0A3R7SIC8\|A0A3R7SIC8_PENVA | 35135.723 | 152.95732 | 2 | 2 |  |
| 692 | T-complex protein 1 subunit delta | A0A423SNI6\|A0A423SNI6_PENVA | 57463.83 | 152.4219 | 2 | 2 |  |
| 693 | RRM domain-containing protein | A0A423SPB5\|A0A423SPB5_PENVA | 60310.637 | 152.26451 | 2 | 2 |  |
| 694 | Adenylyl cyclase-associated protein | A0A3R7PMB6\|A0A3R7PMB6_PENVA | 58966.605 | 152.1816 | 3 | 3 | Carbamidomethylation |
| 695 | Putative serine/threonine-protein phosphatase | A0A3R7PWZ9\|A0A3R7PWZ9_PENVA | 63671.734 | 151.83081 | 2 | 2 | Carbamidomethylation |
| 696 | Putative serine/threonine-protein phosphatase | A0A3R7MT78\|A0A3R7MT78_PENVA | 98152.15 | 151.83081 | 2 | 2 | Carbamidomethylation |
| 697 | Uncharacterized protein | A0A3R7PLR3\|A0A3R7PLR3_PENVA | 224269.98 | 150.1687 | 2 | 2 |  |
| 698 | Putative eukaryotic translation initiation factor 2 subunit 2 isoform X2 | A0A3R7MF11\|A0A3R7MF11_PENVA | 36450.332 | 149.3984 | 3 | 3 |  |
| 699 | Mitochondrial ATP synthase F chain | A0A423T6N8\|A0A423T6N8_PENVA | 12138.055 | 148.90881 | 2 | 2 |  |
| 700 | Nucleosome assembly protein 1-like 4 | A0A423T1L6\|A0A423T1L6_PENVA | 42221.996 | 147.2455 | 2 | 2 |  |
| 701 | protein-serine/threonine phosphatase (Fragment) | A0A423TZ43\|A0A423TZ43_PENVA | 40682.86 | 147.23425 | 2 | 2 |  |
| 702 | Putative nesprin-1 isoform X11 | A0A423T0Y4\|A0A423T0Y4_PENVA | 48195.09 | 146.75186 | 2 | 1 |  |
| 703 | Leucine-rich repeat-containing protein 16A | A0A423U7Z4\|A0A423U7Z4_PENVA | 135162.86 | 146.01404 | 3 | 3 | Acetylation (N-term) |
| 704 | Putative CD81 protein | A0A423T7P8\|A0A423T7P8_PENVA | 44096.316 | 145.90903 | 2 | 2 |  |
| 705 | Putative ubiquitin-conjugating enzyme E2 G1 | A0A423SRQ8\|A0A423SRQ8_PENVA | 18239.695 | 145.27979 | 2 | 2 |  |
| 706 | Cytochrome c oxidase subunit 4 | A0A423U8F6\|A0A423U8F6_PENVA | 20009.236 | 144.88501 | 2 | 2 |  |
| 707 | Secreted protein | A0A3R7MDK8\|A0A3R7MDK8_PENVA | 38744.215 | 143.5311 | 2 | 2 | Carbamidomethylation |
| 708 | ATP synthase-coupling factor 6, mitochondrial | A0A423SS33\|A0A423SS33_PENVA | 12615.422 | 143.21925 | 3 | 3 | Oxidation (M) |
| 709 | Uncharacterized protein | A0A423SV08\|A0A423SV08_PENVA | 234274.17 | 143.09685 | 2 | 1 | Acetylation (N-term) |
| 710 | Uncharacterized protein | A0A3R7SUJ4\|A0A3R7SUJ4_PENVA | 59038.508 | 141.87917 | 2 | 2 | Carbamidomethylation |
| 711 | Ribosomal protein S24 | A0A423SB40\|A0A423SB40_PENVA | 14654.261 | 140.504 | 2 | 2 |  |
| 712 | ATP synthase subunit d, mitochondrial | A0A423SG07\|A0A423SG07_PENVA | 19006.535 | 138.56012 | 2 | 2 |  |
| 713 | Programmed cell death protein 4 | A0A3R7NZL3\|A0A3R7NZL3_PENVA | 50889.285 | 137.60503 | 2 | 2 |  |
| 714 | Putative mitochondrial ribosome-associated GTPase 2 isoform X1 | A0A3R7MAJ5\|A0A3R7MAJ5_PENVA | 47996.453 | 30.639107 | 1 | 1 |  |
| 715 | Ubiquitin-conjugating enzyme | A0A3R7SRB5\|A0A3R7SRB5_PENVA | 66753.91 | 136.29103 | 2 | 2 | Carbamidomethylation |
| 716 | Isoform of A0A3R7P788, Putative tubulin beta-4B chain | A0A423SFE0\|A0A423SFE0_PENVA | 9837.988 | 136.00587 | 2 | 2 |  |
| 717 | VWFA domain-containing protein | A0A423U2M5\|A0A423U2M5_PENVA | 97351.164 | 135.33133 | 3 | 3 | Carbamidomethylation; Deamidation (NQ); Oxidation (M) |
| 718 | Putative thioredoxin-like protein 1 | A0A3R7QFF7\|A0A3R7QFF7_PENVA | 19142.71 | 135.05081 | 2 | 2 |  |
| 719 | Uncharacterized protein | A0A3R7MP64\|A0A3R7MP64_PENVA | 119815.1 | 134.87741 | 2 | 2 |  |
| 720 | Prostaglandin reductase 1 | A0A423TES1\|A0A423TES1_PENVA | 40238.473 | 134.74881 | 4 | 3 | Carbamidomethylation |
| 721 | Prostaglandin reductase 1 | A0A423TER0\|A0A423TER0_PENVA | 37431.22 | 102.58926 | 2 | 2 |  |
| 722 | Putative neuroplastin isoform X1 | A0A3R7MY06\|A0A3R7MY06_PENVA | 30183.293 | 134.35834 | 2 | 2 | Carbamidomethylation; Deamidation (NQ) |
| 723 | Dynein light chain | A0A3R7QHU5\|A0A3R7QHU5_PENVA | 10402.876 | 133.83617 | 2 | 2 | Carbamidomethylation |
| 724 | Long-chain-fatty-acid--CoA ligase | A0A3R7QP52\|A0A3R7QP52_PENVA | 66056.66 | 132.68993 | 2 | 2 | Acetylation (N-term); Carbamidomethylation |
| 725 | Titin | A0A3R7MFP5\|A0A3R7MFP5_PENVA | 156735.38 | 131.64546 | 2 | 2 | Deamidation (NQ) |
| 726 | Ras-like protein | A0A3R7PZR4\|A0A3R7PZR4_PENVA | 21184.236 | 125.46199 | 2 | 1 |  |
| 727 | Putative muscle calcium channel subunit alpha-1 isoform X1 (Fragment) | A0A423T6L3\|A0A423T6L3_PENVA | 131900.44 | 124.60646 | 2 | 2 |  |
| 728 | Putative basement membrane-specific heparan sulfate proteoglycan core protein isoform X13 | A0A423T9T3\|A0A423T9T3_PENVA | 40603.035 | 124.13822 | 1 | 1 | Carbamidomethylation; Deamidation (NQ) |
| 729 | Uncharacterized protein | A0A423TJX4\|A0A423TJX4_PENVA | 23224.201 | 124.06349 | 1 | 1 | Carbamidomethylation |
| 730 | Heat shock protein 40 | A0A3G2YPU5\|A0A3G2YPU5_PENVA | 44368.74 | 124.05434 | 1 | 1 | Carbamidomethylation |
| 731 | Putative phenazine biosynthesis-like domain-containing protein | A0A3R7QFE6\|A0A3R7QFE6_PENVA | 33561.17 | 123.90072 | 1 | 1 | Deamidation (NQ) |
| 732 | Putative lysosomal alpha-glucosidase-like | A0A423SQ98\|A0A423SQ98_PENVA | 16410.117 | 123.79862 | 1 | 1 |  |
| 733 | Cytochrome c oxidase copper chaperone | A0A423U2U9\|A0A423U2U9_PENVA | 16907.297 | 123.79733 | 1 | 1 | Carbamidomethylation |
| 734 | Putative structural constituent of cuticle | A0A423TRF2\|A0A423TRF2_PENVA | 11916.1875 | 123.644684 | 1 | 1 | Deamidation (NQ) |
| 735 | Calcineurin-like phosphoesterase domain-containing protein | A0A3R7N1S8\|A0A3R7N1S8_PENVA | 49356.812 | 123.542816 | 1 | 1 |  |
| 736 | 40S ribosomal protein S12 | A0A423TWD2\|A0A423TWD2_PENVA | 11850.673 | 123.49463 | 1 | 1 | Carbamidomethylation |
| 737 | Putative twinfilin | A0A3R7M6G1\|A0A3R7M6G1_PENVA | 35839.88 | 123.441536 | 1 | 1 |  |
| 738 | Translocon-associated protein subunit delta | A0A3R7PYH8\|A0A3R7PYH8_PENVA | 17631.166 | 123.42765 | 1 | 1 |  |
| 739 | Putative GTP-binding protein SAR1B | A0A3R7QRM9\|A0A3R7QRM9_PENVA | 23479.979 | 123.23949 | 1 | 1 | Deamidation (NQ) |
| 740 | Sorting nexin 12-like protein | A0A3R7QXZ8\|A0A3R7QXZ8_PENVA | 12608.531 | 123.14988 | 1 | 1 |  |
| 741 | Putative leucine-rich glioma-inactivated protein 1-like | A0A3R7MFS1\|A0A3R7MFS1_PENVA | 16944.092 | 123.13623 | 1 | 1 |  |
| 742 | Putative huntingtin-interacting protein K | A0A3R7NN65\|A0A3R7NN65_PENVA | 13257.93 | 122.978195 | 1 | 1 | Acetylation (N-term) |
| 743 | Putative papilin-like isoform X6 | A0A423SN04\|A0A423SN04_PENVA | 134133.77 | 122.95854 | 1 | 1 | Carbamidomethylation |
| 744 | RGS domain-containing protein | A0A423T024\|A0A423T024_PENVA | 19595.074 | 122.68327 | 1 | 1 |  |
| 745 | Trimeric intracellular cation channel type B | A0A3R7QUT9\|A0A3R7QUT9_PENVA | 13542.027 | 122.67965 | 1 | 1 | Oxidation (M) |
| 746 | Uncharacterized protein | A0A423SPE6\|A0A423SPE6_PENVA | 123745.99 | 122.63139 | 1 | 1 |  |
| 747 | Clathrin light chain | A0A3R7SWG8\|A0A3R7SWG8_PENVA | 40365.52 | 122.61565 | 2 | 2 | Acetylation (N-term) |
| 748 | UBC core domain-containing protein | A0A3R7ML74\|A0A3R7ML74_PENVA | 17071.877 | 122.51611 | 1 | 1 |  |
| 749 | Isoform of A0A423SSL9, Uncharacterized protein | A0A3R7LXK4\|A0A3R7LXK4_PENVA | 39538.16 | 122.48741 | 1 | 1 |  |
| 750 | Single VWC domain protein 1 | A0A3R7QJX4\|A0A3R7QJX4_PENVA | 18214.707 | 122.40874 | 1 | 1 | Carbamidomethylation |
| 751 | Succinate--CoA ligase [ADP/GDP-forming] subunit alpha, mitochondrial | A0A3R7PBU9\|A0A3R7PBU9_PENVA | 137061.67 | 122.34581 | 1 | 1 | Carbamidomethylation |
| 752 | Low-density lipoprotein receptor-related protein 2 | A0A423TRM5\|A0A423TRM5_PENVA | 69449.49 | 122.29729 | 1 | 1 | Carbamidomethylation |
| 753 | Protein stum | A0A423T3Q1\|A0A423T3Q1_PENVA | 20574.281 | 122.28902 | 1 | 1 |  |
| 754 | Putative stress-induced-phosphoprotein 1 | A0A423TG20\|A0A423TG20_PENVA | 29688.691 | 122.27839 | 1 | 1 |  |
| 755 | Ubiquinol-cytochrome c reductase, Rieske iron-sulfur polypeptide 1 | A0A3R7Q749\|A0A3R7Q749_PENVA | 28429.584 | 122.25715 | 1 | 1 |  |
| 756 | Mutant C-type lectin | A0A423U9P3\|A0A423U9P3_PENVA | 30106.887 | 122.2383 | 1 | 1 |  |
| 757 | Nesprin-1-like | A0A423SHS2\|A0A423SHS2_PENVA | 104833.836 | 122.23476 | 1 | 1 |  |
| 758 | Putative nesprin-1 isoform X7 | A0A3R7PL73\|A0A3R7PL73_PENVA | 24584.643 | 122.16894 | 1 | 1 | Deamidation (NQ) |
| 759 | Putative heterochromatin protein 1-binding protein 3 isoform X8 | A0A423TDM1\|A0A423TDM1_PENVA | 35855.426 | 122.14667 | 1 | 1 |  |
| 760 | NADH dehydrogenase [ubiquinone] iron-sulfur protein 3, mitochondrial | A0A3R7MHW7\|A0A3R7MHW7_PENVA | 31804.201 | 122.068344 | 1 | 1 |  |
| 761 | NADH dehydrogenase [ubiquinone] iron-sulfur protein 3, mitochondrial | A0A3R7P608\|A0A3R7P608_PENVA | 30842.254 | 122.068344 | 1 | 1 |  |
| 762 | Peptidyl-prolyl cis-trans isomerase (Fragment) | A0A3R7NBX4\|A0A3R7NBX4_PENVA | 15316.131 | 121.985725 | 1 | 1 | Deamidation (NQ) |
| 763 | Phosphorylase b kinase regulatory subunit | A0A3R7MCY6\|A0A3R7MCY6_PENVA | 30551.355 | 121.896576 | 1 | 1 | Deamidation (NQ) |
| 764 | Sarco/endoplasmic reticulum calcium-ATPase isoform 2 | A0A3R7M1B8\|A0A3R7M1B8_PENVA | 13174.079 | 121.82166 | 1 | 1 | Deamidation (NQ) |
| 765 | inorganic diphosphatase | A0A3R7PKM8\|A0A3R7PKM8_PENVA | 36914.008 | 121.735634 | 1 | 1 | Deamidation (NQ) |
| 766 | Isoform of A0A3R7PKM8, inorganic diphosphatase | A0A423U4P7\|A0A423U4P7_PENVA | 12240.878 | 121.735634 | 1 | 1 | Deamidation (NQ) |
| 767 | C2 domain-containing protein | A0A3R7PDT2\|A0A3R7PDT2_PENVA | 27368.852 | 121.720764 | 1 | 1 |  |
| 768 | Putative host cell factor 1-like | A0A423TUW0\|A0A423TUW0_PENVA | 86878 | 121.67624 | 1 | 1 |  |
| 769 | Uncharacterized protein | A0A423TFX1\|A0A423TFX1_PENVA | 51979.453 | 121.669395 | 1 | 1 |  |
| 770 | Ribosomal protein P1 | A0A423SWS8\|A0A423SWS8_PENVA | 9893.185 | 121.66028 | 1 | 1 |  |
| 771 | Activation factor subunit spp27 | A0A423TC50\|A0A423TC50_PENVA | 24541.543 | 121.65459 | 1 | 1 |  |
| 772 | EF-hand domain-containing protein (Fragment) | A0A423T1C3\|A0A423T1C3_PENVA | 20914.703 | 121.60227 | 1 | 1 |  |
| 773 | Uncharacterized oxidoreductase | A0A3R7MFJ8\|A0A3R7MFJ8_PENVA | 46061.355 | 121.53541 | 1 | 1 |  |
| 774 | Coatomer subunit beta' | A0A423TM28\|A0A423TM28_PENVA | 111144.24 | 121.53541 | 1 | 1 |  |
| 775 | NADH dehydrogenase | A0A3R7QEY9\|A0A3R7QEY9_PENVA | 13176.27 | 121.529755 | 1 | 1 |  |
| 776 | Putative cytosolic non-specific dipeptidase | A0A3R7PII9\|A0A3R7PII9_PENVA | 53269.99 | 121.51506 | 1 | 1 |  |
| 777 | Putative cytosolic non-specific dipeptidase | A0A423STZ9\|A0A423STZ9_PENVA | 32371.99 | 121.51506 | 1 | 1 |  |
| 778 | Rhodanese domain-containing protein | A0A3R7PND5\|A0A3R7PND5_PENVA | 16540.8 | 121.509415 | 1 | 1 | Deamidation (NQ) |
| 779 | Calcineurin subunit B | A0A3R7QTN8\|A0A3R7QTN8_PENVA | 18438.967 | 121.509415 | 1 | 1 |  |
| 780 | Saposin isoform 1 | A0A423UA58\|A0A423UA58_PENVA | 39262.348 | 121.508286 | 1 | 1 | Carbamidomethylation |
| 781 | Saposin isoform 1 | A0A423UA78\|A0A423UA78_PENVA | 95618.24 | 121.508286 | 1 | 1 | Carbamidomethylation |
| 782 | non-specific serine/threonine protein kinase | A0A3R7LXP0\|A0A3R7LXP0_PENVA | 73987.4 | 121.50603 | 1 | 1 |  |
| 783 | Cyclase | A0A3R7MDC5\|A0A3R7MDC5_PENVA | 28788.705 | 121.50264 | 1 | 1 |  |
| 784 | DDB1-and CUL4-associated factor 6 | A0A3R7PFT2\|A0A3R7PFT2_PENVA | 84186.69 | 121.4193 | 1 | 1 |  |
| 785 | glutathione transferase | A0A3R7PVQ7\|A0A3R7PVQ7_PENVA | 28210.436 | 121.39796 | 1 | 1 |  |
| 786 | Crustacean hematopoietic factor-like protein | A0A423TXY6\|A0A423TXY6_PENVA | 31091.89 | 121.36433 | 1 | 1 | Carbamidomethylation |
| 787 | Thioredoxin-related transmembrane protein 1 | A0A423U5X6\|A0A423U5X6_PENVA | 25697.781 | 121.34418 | 1 | 1 |  |
| 788 | aldehyde dehydrogenase (NAD(+)) | A0A3R7PT34\|A0A3R7PT34_PENVA | 57560.11 | 121.26938 | 1 | 1 |  |
| 789 | Angiopoietin-related protein 1 | A0A423TSJ7\|A0A423TSJ7_PENVA | 54010.41 | 121.25713 | 1 | 1 |  |
| 790 | Malate dehydrogenase, cytoplasmic (Fragment) | A0A3R7LXH7\|A0A3R7LXH7_PENVA | 14028.453 | 121.24267 | 1 | 1 |  |
| 791 | Large ribosomal subunit protein uL22 | A0A423THI6\|A0A423THI6_PENVA | 21213.842 | 121.224884 | 1 | 1 | Carbamidomethylation |
| 792 | Putative GRIP1-associated protein 1-like (Fragment) | A0A3R7QYE0\|A0A3R7QYE0_PENVA | 62368.457 | 121.21932 | 1 | 1 |  |
| 793 | Uncharacterized protein | A0A3R7MZJ7\|A0A3R7MZJ7_PENVA | 40359.805 | 121.20378 | 1 | 1 | Carbamidomethylation |
| 794 | Crustacyanin subunit A | A0A423SES2\|A0A423SES2_PENVA | 24356.424 | 121.139534 | 1 | 1 | Carbamidomethylation |
| 795 | Eukaryotic translation initiation factor 2A | A0A3R7PUY1\|A0A3R7PUY1_PENVA | 65229.414 | 121.08655 | 1 | 1 |  |
| 796 | RNA recognition motif (RRM) | A0A3R7T1U5\|A0A3R7T1U5_PENVA | 31521.701 | 121.057915 | 1 | 1 |  |
| 797 | Large ribosomal subunit protein uL14 | A0A423TLX9\|A0A423TLX9_PENVA | 30086.797 | 121.00188 | 1 | 1 |  |
| 798 | Eukaryotic translation initiation factor 3 subunit B | A0A3R7MBC4\|A0A3R7MBC4_PENVA | 80568.5 | 120.95696 | 1 | 1 |  |
| 799 | NAD-dependent deacetylase sirtuin-2 | A0A3R7M8U0\|A0A3R7M8U0_PENVA | 53566.81 | 120.8479 | 1 | 1 | Acetylation (N-term) |
| 800 | Endoribonuclease | A0A3R7LWS7\|A0A3R7LWS7_PENVA | 35109.2 | 120.818565 | 1 | 1 | Deamidation (NQ) |
| 801 | Transporter | A0A423TZJ1\|A0A423TZJ1_PENVA | 67940.53 | 120.554665 | 1 | 1 |  |
| 802 | Putative collagen alpha chain, type IV (Fragment) | A0A3R7QTI2\|A0A3R7QTI2_PENVA | 27415.426 | 120.52262 | 1 | 1 |  |
| 803 | Sulfatase N-terminal domain-containing protein | A0A423TS42\|A0A423TS42_PENVA | 59753.93 | 120.469345 | 1 | 1 |  |
| 804 | Proteasome subunit alpha type | A0A3R7MGN6\|A0A3R7MGN6_PENVA | 31286.88 | 119.80562 | 1 | 1 | Carbamidomethylation; Deamidation (NQ) |
| 805 | Putative gamma-subunit,methylmalonyl-CoA decarboxylase | A0A3R7MIR8\|A0A3R7MIR8_PENVA | 11243.355 | 119.48102 | 1 | 1 | Carbamidomethylation |
| 806 | Phosphotriesterase-related protein | A0A3R7LYV0\|A0A3R7LYV0_PENVA | 39356.746 | 119.18211 | 1 | 1 | Carbamidomethylation |
| 807 | T-complex protein 1 subunit alpha | A0A3R7SYF7\|A0A3R7SYF7_PENVA | 59799.76 | 118.998764 | 2 | 2 | Carbamidomethylation |
| 808 | Retinal dehydrogenase 1 | A0A423SGM7\|A0A423SGM7_PENVA | 58607.38 | 118.96224 | 1 | 1 | Carbamidomethylation |
| 809 | Peroxiredoxin | D0FH89\|D0FH89_PENVA | 22010.197 | 118.63928 | 2 | 2 |  |
| 810 | Voltage-dependent L-type calcium channel subunit alpha | A0A3R7M520\|A0A3R7M520_PENVA | 89853.32 | 118.63469 | 1 | 1 |  |
| 811 | Alpha-galactosidase | A0A3R7M4M2\|A0A3R7M4M2_PENVA | 56343.19 | 118.38287 | 1 | 1 |  |
| 812 | Putative BAG domain-containing protein Samui isoform X1 | A0A423U3C8\|A0A423U3C8_PENVA | 74541.305 | 117.85137 | 2 | 2 |  |
| 813 | BAG domain-containing protein | A0A423SBB4\|A0A423SBB4_PENVA | 60961.36 | 59.062763 | 1 | 1 |  |
| 814 | Putative neurofilament heavy polypeptide | A0A423TBN5\|A0A423TBN5_PENVA | 22135.562 | 117.84139 | 1 | 1 |  |
| 815 | Peptidyl-prolyl cis-trans isomerase | A0A3R7N980\|A0A3R7N980_PENVA | 24746.092 | 117.62537 | 2 | 2 |  |
| 816 | C-type lectin | A0A3R7QW14\|A0A3R7QW14_PENVA | 18640.91 | 117.51976 | 1 | 1 | Deamidation (NQ) |
| 817 | Phosphoglycolate phosphatase | A0A3R7Q051\|A0A3R7Q051_PENVA | 35542.777 | 117.411194 | 2 | 2 | Carbamidomethylation |
| 818 | T-complex protein 1 subunit beta | A0A423SHB5\|A0A423SHB5_PENVA | 56943.434 | 116.670135 | 1 | 1 |  |
| 819 | Peritrophin-44-like protein | A0A3R7Q1E9\|A0A3R7Q1E9_PENVA | 24268.28 | 116.45303 | 3 | 3 | Carbamidomethylation |
| 820 | Crustacyanin subunit C | A0A3R7P6V3\|A0A3R7P6V3_PENVA | 22387.07 | 116.37167 | 2 | 1 | Deamidation (NQ) |
| 821 | Crustacyanin subunit C | A0A423SEQ1\|A0A423SEQ1_PENVA | 24392.209 | 116.37167 | 2 | 1 | Deamidation (NQ) |
| 822 | Crustacyanin subunit C | A0A3R7NNR5\|A0A3R7NNR5_PENVA | 12975.697 | 84.15084 | 1 | 1 |  |
| 823 | GTP binding protein alpha subunit Go | A0A423SF76\|A0A423SF76_PENVA | 28772.541 | 116.32902 | 1 | 1 |  |
| 824 | Sex-lethal | A0A423TSY3\|A0A423TSY3_PENVA | 23396.5 | 116.17131 | 1 | 1 |  |
| 825 | Uncharacterized protein | A0A3R7MJR0\|A0A3R7MJR0_PENVA | 164042.58 | 116.15981 | 2 | 2 |  |
| 826 | Small androgen receptor-interacting protein 1 | A0A423TFY7\|A0A423TFY7_PENVA | 28464.258 | 115.38873 | 2 | 1 | Acetylation (N-term) |
| 827 | Rab1A | A0A423TSB0\|A0A423TSB0_PENVA | 22810.887 | 115.275246 | 3 | 3 |  |
| 828 | Rab10 | A0A3R7M456\|A0A3R7M456_PENVA | 23066.455 | 107.71337 | 2 | 2 |  |
| 829 | Ras-related protein Rab-33B | A0A423SCL7\|A0A423SCL7_PENVA | 26593.264 | 62.947296 | 1 | 1 |  |
| 830 | Putative ras-related protein Rab-30 | A0A3R7PF71\|A0A3R7PF71_PENVA | 24701.072 | 62.947296 | 1 | 1 |  |
| 831 | Rab14 | A0A3R7LX90\|A0A3R7LX90_PENVA | 19937.137 | 62.947296 | 1 | 1 |  |
| 832 | Putative ras-related protein Rab-37 isoform X1 | A0A3R7M6D3\|A0A3R7M6D3_PENVA | 42113.477 | 62.947296 | 1 | 1 |  |
| 833 | Putative Rab-43-like protein | A0A423SEJ9\|A0A423SEJ9_PENVA | 25137.697 | 62.947296 | 1 | 1 |  |
| 834 | Putative EF-hand calcium-binding domain-containing protein 4B | A0A3R7PDS3\|A0A3R7PDS3_PENVA | 49645.81 | 62.947296 | 1 | 1 |  |
| 835 | Putative RAS-related protein | A0A3R7LUN2\|A0A3R7LUN2_PENVA | 20828.643 | 62.947296 | 1 | 1 |  |
| 836 | Ras-related protein Rab-8A | A0A423TT98\|A0A423TT98_PENVA | 35952.58 | 62.947296 | 1 | 1 |  |
| 837 | Rab GTPase | A0A3R7PDP2\|A0A3R7PDP2_PENVA | 23737.91 | 62.947296 | 1 | 1 |  |
| 838 | >sp\|NEDD8_HUMAN\| | #CONTAM#NEDD8_HUMAN\| | 9071.548 | 115.262695 | 1 | 1 |  |
| 839 | NEDD8 | A0A423UAI3\|A0A423UAI3_PENVA | 9005.46 | 115.262695 | 1 | 1 |  |
| 840 | >sp\|ALBU_BOVIN\| | #CONTAM#ALBU_BOVIN\| | 69293.41 | 115.19111 | 1 | 1 |  |
| 841 | Ribosomal protein L19 | A0A423TID6\|A0A423TID6_PENVA | 22755.99 | 114.94891 | 2 | 2 | Deamidation (NQ) |
| 842 | Glutathione S-transferase | A0A3R7SMK3\|A0A3R7SMK3_PENVA | 27370.574 | 113.828186 | 1 | 1 |  |
| 843 | 3-hydroxyacyl-CoA dehydrogenase type-2 | A0A423U2A1\|A0A423U2A1_PENVA | 23309.068 | 113.534546 | 1 | 1 |  |
| 844 | Putative heat shock factor-binding protein 1-like | A0A3R7PCG6\|A0A3R7PCG6_PENVA | 9813.037 | 113.330925 | 1 | 1 |  |
| 845 | >sp\|PPIA_HUMAN\| | #CONTAM#PPIA_HUMAN\| | 17881.297 | 112.969345 | 1 | 1 | Acetylation (N-term) |
| 846 | Mitochondrial cytochrome c oxidase subunit VIb | A0A3R7QFM7\|A0A3R7QFM7_PENVA | 15555.558 | 112.26439 | 4 | 4 | Carbamidomethylation |
| 847 | N-acetylated-alpha-linked acidic dipeptidase 2 | A0A423TNU3\|A0A423TNU3_PENVA | 84014.14 | 110.98068 | 1 | 1 |  |
| 848 | T-complex protein 1 subunit zeta | A0A423TPV2\|A0A423TPV2_PENVA | 84304.05 | 110.65106 | 2 | 2 |  |
| 849 | Isoform of A0A3R7SKX2, Putative eukaryotic translation initiation factor 2 subunit 3 | A0A423SLZ6\|A0A423SLZ6_PENVA | 31001.336 | 110.36218 | 1 | 1 |  |
| 850 | protein-synthesizing GTPase | A0A3R7SKX2\|A0A3R7SKX2_PENVA | 50400.914 | 110.36218 | 1 | 1 |  |
| 851 | Putative myosin light chain kinase | A0A3R7MMZ5\|A0A3R7MMZ5_PENVA | 84979.375 | 110.16415 | 1 | 1 | Carbamidomethylation |
| 852 | RNA helicase | A0A423T420\|A0A423T420_PENVA | 45450.652 | 109.70875 | 1 | 1 |  |
| 853 | Eukaryotic initiation factor 4A | A0A3R7SRV6\|A0A3R7SRV6_PENVA | 41193.062 | 109.70875 | 1 | 1 |  |
| 854 | PITH domain-containing protein | A0A3R7PQE0\|A0A3R7PQE0_PENVA | 18830.336 | 109.02497 | 1 | 1 | Carbamidomethylation; Deamidation (NQ) |
| 855 | Chloride intracellular channel | A0A423SDD3\|A0A423SDD3_PENVA | 21711.986 | 108.77574 | 1 | 1 | Deamidation (NQ) |
| 856 | Isoform of A0A423TGZ0, Receptor expression-enhancing protein | A0A3R7PTC9\|A0A3R7PTC9_PENVA | 21424.002 | 106.664734 | 2 | 2 |  |
| 857 | Putative hsp90 co-chaperone Cdc37 | A0A423SPS6\|A0A423SPS6_PENVA | 25937.695 | 106.30303 | 1 | 1 |  |
| 858 | Putative transketolase-like protein 2 isoform X1 | A0A423SA92\|A0A423SA92_PENVA | 67768.11 | 105.27642 | 1 | 1 |  |
| 859 | Electron transfer flavoprotein subunit beta | A0A3R7R0B0\|A0A3R7R0B0_PENVA | 27700.434 | 104.813705 | 1 | 1 |  |
| 860 | Microtubule-associated protein RP/EB family member 3 | A0A3R7QM52\|A0A3R7QM52_PENVA | 29625.688 | 104.180435 | 1 | 1 | Carbamidomethylation |
| 861 | Putative beta-tubulin | A0A423SFE4\|A0A423SFE4_PENVA | 90612.69 | 102.78441 | 1 | 1 | Carbamidomethylation |
| 862 | Ubiquitin carboxyl-terminal hydrolase | A0A423T541\|A0A423T541_PENVA | 41618.305 | 102.73337 | 1 | 1 |  |
| 863 | 60S ribosomal protein L12 | A0A423TXI0\|A0A423TXI0_PENVA | 17838.873 | 102.70186 | 2 | 2 | Deamidation (NQ) |
| 864 | Putative heterogeneous nuclear ribonucleoprotein A1, A2/B1-like | A0A423SS06\|A0A423SS06_PENVA | 34751.555 | 101.79933 | 1 | 1 |  |
| 865 | Putative heterogeneous nuclear ribonucleoprotein A1, A2/B1-like isoform X1 | A0A3R7MPR7\|A0A3R7MPR7_PENVA | 38689.367 | 101.79933 | 1 | 1 |  |
| 866 | Resistance to inhibitors of cholinesterase protein 3 N-terminal domain-containing protein | A0A3R7PC60\|A0A3R7PC60_PENVA | 86157.13 | 101.59211 | 1 | 1 |  |
| 867 | Uncharacterized protein (Fragment) | A0A3R7NC20\|A0A3R7NC20_PENVA | 75263.97 | 101.59211 | 1 | 1 |  |
| 868 | 40S ribosomal protein SA | A0A3R7LY48\|A0A3R7LY48_PENVA | 28454.156 | 101.34355 | 2 | 2 |  |
| 869 | Small ribosomal subunit protein uS2 | A0A423TDL6\|A0A423TDL6_PENVA | 29590.408 | 101.34355 | 2 | 2 |  |
| 870 | 40S ribosomal protein SA | A0A423TTG8\|A0A423TTG8_PENVA | 17358.922 | 67.93755 | 1 | 1 |  |
| 871 | Crustacyanin subunit A | A0A3R7MIM3\|A0A3R7MIM3_PENVA | 21396.08 | 101.199234 | 2 | 1 | Carbamidomethylation; Deamidation (NQ) |
| 872 | Crustacyanin subunit A | A0A423SEP5\|A0A423SEP5_PENVA | 21372.059 | 101.199234 | 2 | 1 | Carbamidomethylation; Deamidation (NQ) |
| 873 | Isoform of A0A3R7MIM3, Crustacyanin subunit A | A0A3R7LRR6\|A0A3R7LRR6_PENVA | 19659.992 | 88.06776 | 1 | 1 | Carbamidomethylation |
| 874 | Phosducin thioredoxin-like domain-containing protein | A0A423U6G3\|A0A423U6G3_PENVA | 27606.148 | 101.08672 | 1 | 1 |  |
| 875 | Sodium-calcium exchanger 1 isoform 1 | A0A3R7Q5S5\|A0A3R7Q5S5_PENVA | 65138.105 | 100.03875 | 1 | 1 | Deamidation (NQ) |
| 876 | Glutathione peroxidase 6 | A0A3R7PJ04\|A0A3R7PJ04_PENVA | 11269.853 | 99.96017 | 1 | 1 |  |
| 877 | S-adenosylmethionine synthase | A0A3R7MHS2\|A0A3R7MHS2_PENVA | 44234.117 | 99.259384 | 1 | 1 | Carbamidomethylation |
| 878 | Coronin | A0A423T0V7\|A0A423T0V7_PENVA | 58109.824 | 98.588 | 2 | 2 |  |
| 879 | Glycogen synthase kinase 3 beta | A0A423T7S5\|A0A423T7S5_PENVA | 47999.492 | 85.222115 | 1 | 1 |  |
| 880 | Putative serine/arginine-rich splicing factor 7-like | A0A3R7PCB5\|A0A3R7PCB5_PENVA | 41158.434 | 97.51704 | 1 | 1 |  |
| 881 | Protein quiver | A0A3R7MKU8\|A0A3R7MKU8_PENVA | 23164.51 | 97.26541 | 1 | 1 |  |
| 882 | >sp\|K22E_HUMAN\| | #CONTAM#K22E_HUMAN\| | 65865.32 | 95.64836 | 2 | 1 |  |
| 883 | Putative laminin A chain | A0A423T016\|A0A423T016_PENVA | 40590.3 | 94.39723 | 2 | 2 | Carbamidomethylation; Deamidation (NQ) |
| 884 | RNA recognition motif (RRM) | A0A3R7LX65\|A0A3R7LX65_PENVA | 37723.98 | 93.20951 | 1 | 1 |  |
| 885 | Small ribosomal subunit protein uS10 | A0A3R7M5H8\|A0A3R7M5H8_PENVA | 14424.856 | 93.09452 | 2 | 2 |  |
| 886 | Glycogen [starch] synthase | A0A423TSU1\|A0A423TSU1_PENVA | 74594.92 | 92.98828 | 2 | 2 |  |
| 887 | Glycogen-binding subunit 76A | A0A3R7QIC8\|A0A3R7QIC8_PENVA | 128508.81 | 92.57919 | 1 | 1 |  |
| 888 | I/LWEQ domain-containing protein | A0A423SL96\|A0A423SL96_PENVA | 63928.348 | 91.70182 | 1 | 1 |  |
| 889 | Uncharacterized protein | A0A3R7MJI6\|A0A3R7MJI6_PENVA | 13002.786 | 90.512596 | 1 | 1 |  |
| 890 | Pyruvate dehydrogenase E1 component subunit beta | A0A3R7PI62\|A0A3R7PI62_PENVA | 44203.793 | 88.50508 | 2 | 2 |  |
| 891 | Large ribosomal subunit protein uL13 | A0A3R7Q736\|A0A3R7Q736_PENVA | 23310.986 | 88.13163 | 1 | 1 | Acetylation (N-term) |
| 892 | Putative FH1/FH2 domain-containing protein 3 | A0A423T0H5\|A0A423T0H5_PENVA | 166254.19 | 87.59227 | 1 | 1 |  |
| 893 | Large ribosomal subunit protein eL28 | A0A3R7MP37\|A0A3R7MP37_PENVA | 16806.723 | 87.568886 | 1 | 1 |  |
| 894 | SHSP domain-containing protein | A0A3R7NMZ6\|A0A3R7NMZ6_PENVA | 248301.34 | 87.3685 | 1 | 1 |  |
| 895 | Trehalose-6-phosphate synthase | A0A423TFS5\|A0A423TFS5_PENVA | 103798.44 | 87.08923 | 2 | 2 | Carbamidomethylation |
| 896 | Trehalose-6-phosphate synthase | A0A3R7PL38\|A0A3R7PL38_PENVA | 72633.664 | 61.66652 | 1 | 1 | Carbamidomethylation |
| 897 | Transforming growth factor-beta-induced protein ig-h3 | A0A3R7MHP7\|A0A3R7MHP7_PENVA | 37714.06 | 86.905655 | 2 | 2 |  |
| 898 | Prolyl 3-hydroxylase 1 | A0A423T7Z1\|A0A423T7Z1_PENVA | 49311.977 | 86.840614 | 1 | 1 |  |
| 899 | 26S protease regulatory subunit 7 | A0A3R7MC01\|A0A3R7MC01_PENVA | 48479.785 | 86.34545 | 1 | 1 |  |
| 900 | Putative beta-ureidopropionase-like | A0A3R7PE77\|A0A3R7PE77_PENVA | 42828.676 | 85.299484 | 1 | 1 | Acetylation (N-term); Carbamidomethylation |
| 901 | Putative 14 kDa phosphohistidine phosphatase-like | A0A3R7PFF9\|A0A3R7PFF9_PENVA | 13529.199 | 84.85937 | 1 | 1 |  |
| 902 | Putative serine/threonine-protein phosphatase 2A 56 kDa regulatory subunit epsilon isoform | A0A3R7QI61\|A0A3R7QI61_PENVA | 40697.53 | 84.59972 | 1 | 1 |  |
| 903 | Putative myosin regulatory light chain 2 smooth muscle | A0A423SLL0\|A0A423SLL0_PENVA | 19786.086 | 84.20246 | 1 | 1 |  |
| 904 | Nucleoside phosphorylase domain-containing protein (Fragment) | A0A3R7MTI6\|A0A3R7MTI6_PENVA | 17571.004 | 83.90603 | 1 | 1 | Carbamidomethylation; Deamidation (NQ) |
| 905 | Putative lamin Dm0 | A0A3R7M1D2\|A0A3R7M1D2_PENVA | 15661.704 | 83.37954 | 1 | 1 |  |
| 906 | Small ribosomal subunit protein uS12 | A0A3R7MJ77\|A0A3R7MJ77_PENVA | 19582.658 | 83.06383 | 1 | 1 | Carbamidomethylation |
| 907 | 4F2 cell-surface antigen heavy chain | A0A3R7PZ26\|A0A3R7PZ26_PENVA | 50047.535 | 81.32271 | 1 | 1 |  |
| 908 | Rab GTPase (Fragment) | A0A3R7QMV3\|A0A3R7QMV3_PENVA | 22483.428 | 80.64716 | 1 | 1 |  |
| 909 | Integrin-linked protein kinase | A0A3R7PXY3\|A0A3R7PXY3_PENVA | 50397.168 | 78.93203 | 1 | 1 |  |
| 910 | 26S proteasome non-ATPase regulatory subunit 4 | A0A3R7MGC1\|A0A3R7MGC1_PENVA | 41299.4 | 78.30596 | 1 | 1 | Oxidation (M) |
| 911 | Putative 26S proteasome non-ATPase regulatory subunit 4 | A0A3R7Q3T7\|A0A3R7Q3T7_PENVA | 38571.113 | 78.30596 | 1 | 1 | Oxidation (M) |
| 912 | Uncharacterized protein | A0A423T686\|A0A423T686_PENVA | 448202.44 | 78.22753 | 1 | 1 |  |
| 913 | Heterochromatin-associated protein HP1 (Fragment) | A0A423SYV8\|A0A423SYV8_PENVA | 16532.318 | 77.822296 | 1 | 1 | Carbamidomethylation |
| 914 | 60S ribosomal protein L13 | A0A423UAM6\|A0A423UAM6_PENVA | 24266.645 | 77.26802 | 2 | 2 | Oxidation (M) |
| 915 | Large proline-rich protein BAG6 | A0A423TG42\|A0A423TG42_PENVA | 153075.62 | 76.85128 | 1 | 1 |  |
| 916 | Endothelial differentiation-related factor 1-like protein | A0A423SAJ2\|A0A423SAJ2_PENVA | 16627.918 | 75.984184 | 1 | 1 |  |
| 917 | Chitin-binding type-2 domain-containing protein | A0A3R7M838\|A0A3R7M838_PENVA | 29650.016 | 75.595215 | 1 | 1 | Carbamidomethylation |
| 918 | Dipeptidyl peptidase 1 | A0A423SFG7\|A0A423SFG7_PENVA | 50266.652 | 75.58862 | 1 | 1 |  |
| 919 | Lethal(2) giant larvae protein-like protein | A0A3R7LXJ7\|A0A3R7LXJ7_PENVA | 113792.555 | 74.223724 | 1 | 1 |  |
| 920 | Adenosylhomocysteinase | A0A423T3K1\|A0A423T3K1_PENVA | 52077.82 | 72.96963 | 1 | 1 | Carbamidomethylation |
| 921 | JHE-like carboxylesterase 2 | A0A3R7Q273\|A0A3R7Q273_PENVA | 84852.21 | 71.23601 | 1 | 1 |  |
| 922 | non-specific serine/threonine protein kinase | A0A3R7QQE6\|A0A3R7QQE6_PENVA | 228741.2 | 71.08826 | 4 | 2 | Acetylation (N-term) |
| 923 | Protein containing Polysaccharide lyase family 8, central | A0A3R7QLT7\|A0A3R7QLT7_PENVA | 80886.05 | 70.10113 | 1 | 1 |  |
| 924 | VCBS repeat-containing protein | A0A423TV81\|A0A423TV81_PENVA | 45252.844 | 69.9798 | 1 | 1 |  |
| 925 | Serine/threonine-protein phosphatase | A0A3R7PY34\|A0A3R7PY34_PENVA | 35017.785 | 69.180626 | 1 | 1 |  |
| 926 | Putative RING finger protein nhl-1 | A0A3R7PWY5\|A0A3R7PWY5_PENVA | 122975.195 | 69.143394 | 1 | 1 |  |
| 927 | WH2 domain-containing protein | A0A423UB10\|A0A423UB10_PENVA | 73994.53 | 68.865234 | 1 | 1 |  |
| 928 | Masquerade-like serine proteinase-like protein 3 | A0A3R7Q840\|A0A3R7Q840_PENVA | 57737.895 | 68.42634 | 1 | 1 | Carbamidomethylation |
| 929 | Protein msta, isoform A | A0A423TJA9\|A0A423TJA9_PENVA | 53751.777 | 67.91977 | 1 | 1 |  |
| 930 | Dipeptidyl peptidase 1 | A0A423T7S3\|A0A423T7S3_PENVA | 43545.008 | 67.58096 | 2 | 2 |  |
| 931 | Protein CASC3 | A0A3R7PET7\|A0A3R7PET7_PENVA | 83870.836 | 67.35455 | 1 | 1 |  |
| 932 | Chitin-binding type-2 domain-containing protein | A0A3R7Q691\|A0A3R7Q691_PENVA | 26653.506 | 66.646454 | 1 | 1 | Carbamidomethylation |
| 933 | Nesprin-1-like | A0A3R7PYS0\|A0A3R7PYS0_PENVA | 587723.3 | 66.443565 | 2 | 1 | Deamidation (NQ) |
| 934 | Putative fatty aldehyde dehydrogenase-like | A0A423TET4\|A0A423TET4_PENVA | 41991.316 | 65.96335 | 2 | 2 |  |
| 935 | WSSV receptor Rab7 | A0A3R7PEU4\|A0A3R7PEU4_PENVA | 40687.527 | 65.833336 | 2 | 2 |  |
| 936 | Calpain B (Fragment) | A0A3R7NE38\|A0A3R7NE38_PENVA | 79259.016 | 64.94091 | 1 | 1 |  |
| 937 | Cuticular protein 34 | A0A423TEF3\|A0A423TEF3_PENVA | 196899.95 | 64.82723 | 1 | 1 |  |
| 938 | Interferon-inducible double stranded RNA-dependent protein kinase activator A | F5AW41\|F5AW41_PENVA | 36834.83 | 64.02511 | 1 | 1 |  |
| 939 | UTP--glucose-1-phosphate uridylyltransferase | A0A3R7PVK4\|A0A3R7PVK4_PENVA | 65129.47 | 63.62156 | 1 | 1 |  |
| 940 | Proteasome subunit beta | A0A3R7SKK1\|A0A3R7SKK1_PENVA | 25888.562 | 63.6039 | 1 | 1 |  |
| 941 | U1 small nuclear ribonucleoprotein 70 kDa-like | A0A3R7PQQ0\|A0A3R7PQQ0_PENVA | 35663.395 | 63.601 | 1 | 1 |  |
| 942 | Hsc70-interacting protein | A0A423SW82\|A0A423SW82_PENVA | 43016.47 | 62.599777 | 2 | 2 | Acetylation (N-term) |
| 943 | Hsc70-interacting protein (Fragment) | A0A423TI26\|A0A423TI26_PENVA | 24984.027 | 28.003149 | 1 | 1 | Acetylation (N-term) |
| 944 | Uncharacterized protein | A0A3R7PI12\|A0A3R7PI12_PENVA | 243074.42 | 61.851376 | 1 | 1 |  |
| 945 | Endothelin-converting enzyme 1 | A0A3R7MJP1\|A0A3R7MJP1_PENVA | 59601.914 | 61.851376 | 1 | 1 |  |
| 946 | Putative programmed cell death protein 2 | A0A3R7SVI2\|A0A3R7SVI2_PENVA | 42237.684 | 61.44581 | 1 | 1 |  |
| 947 | Aldehyde oxidase/xanthine dehydrogenase a/b hammerhead domain-containing protein | A0A423SVY7\|A0A423SVY7_PENVA | 85266.25 | 59.959908 | 1 | 1 |  |
| 948 | Plasmolipin isoform 2 | A0A423TVB4\|A0A423TVB4_PENVA | 26488.012 | 59.840942 | 2 | 2 |  |
| 949 | Ubiquitin carboxyl-terminal hydrolase | A0A3R7LP93\|A0A3R7LP93_PENVA | 20701.104 | 58.638706 | 1 | 1 |  |
| 950 | Putative repetitive proline-rich cell wall protein 2-like | A0A3R7QJX5\|A0A3R7QJX5_PENVA | 156029.02 | 58.59562 | 1 | 1 |  |
| 951 | Putative serine/threonine-protein phosphatase 6 regulatory subunit 3 isoform X1 | A0A3R7PVR4\|A0A3R7PVR4_PENVA | 79564.43 | 58.585564 | 1 | 1 |  |
| 952 | Peritrophin-44-like protein | A0A423T995\|A0A423T995_PENVA | 24044.07 | 58.469265 | 1 | 1 | Carbamidomethylation |
| 953 | Uncharacterized protein | A0A3R7N8P0\|A0A3R7N8P0_PENVA | 59767.707 | 58.306126 | 1 | 1 |  |
| 954 | Mirror | A0A3R7P082\|A0A3R7P082_PENVA | 50262.656 | 57.9617 | 1 | 1 |  |
| 955 | Adenylate kinase 2, mitochondrial | A0A3R7PQF1\|A0A3R7PQF1_PENVA | 26670.531 | 57.48553 | 1 | 1 | Deamidation (NQ) |
| 956 | Glucosamine-6-phosphate isomerase | A0A423TR93\|A0A423TR93_PENVA | 22635.71 | 56.97455 | 1 | 1 | Carbamidomethylation |
| 957 | Putative mediator of RNA polymerase II transcription subunit 26 | A0A3R7SYI9\|A0A3R7SYI9_PENVA | 27753.936 | 56.953957 | 1 | 1 | Deamidation (NQ) |
| 958 | Acyl carrier protein | A0A3R7PUS3\|A0A3R7PUS3_PENVA | 24363.072 | 56.7586 | 2 | 2 |  |
| 959 | Oplophorus-luciferin 2-monooxygenase non-catalytic subunit | A0A3R7M948\|A0A3R7M948_PENVA | 34245.22 | 56.594185 | 1 | 1 |  |
| 960 | Uncharacterized protein | A0A3R7NUR5\|A0A3R7NUR5_PENVA | 65479.387 | 55.594883 | 1 | 1 |  |
| 961 | 3-hydroxyisobutyryl-CoA hydrolase, mitochondrial | A0A3R7QX48\|A0A3R7QX48_PENVA | 39422.42 | 55.42932 | 1 | 1 | Carbamidomethylation |
| 962 | Putative cytochrome b-c1 complex subunit 9 | A0A423T4F2\|A0A423T4F2_PENVA | 26736.709 | 53.85043 | 1 | 1 |  |
| 963 | Metalloendopeptidase | A0A423S926\|A0A423S926_PENVA | 28865.055 | 53.787434 | 1 | 1 |  |
| 964 | DnaJ protein-like 1-like | A0A3R7Q3Q8\|A0A3R7Q3Q8_PENVA | 16703.623 | 53.372627 | 1 | 1 |  |
| 965 | DnaJ protein-like 1-like | A0A3R7Q013\|A0A3R7Q013_PENVA | 39653.812 | 53.372627 | 1 | 1 |  |
| 966 | Uncharacterized protein | A0A3R7PV73\|A0A3R7PV73_PENVA | 39112.234 | 53.040314 | 1 | 1 |  |
| 967 | GDNF/GAS1 domain-containing protein | A0A423U1G1\|A0A423U1G1_PENVA | 37229.18 | 53.040314 | 1 | 1 |  |
| 968 | Putative proliferation-associated protein 2G4 | A0A3R7QDZ5\|A0A3R7QDZ5_PENVA | 42836.5 | 51.017197 | 1 | 1 |  |
| 969 | Lymphoid organ expressed yellow head virus receptor protein | A0A3R7NER9\|A0A3R7NER9_PENVA | 50853.918 | 50.880856 | 1 | 1 |  |
| 970 | Hemocyte transglutaminase | A0A3R7LU97\|A0A3R7LU97_PENVA | 44897.29 | 50.842777 | 1 | 1 |  |
| 971 | Isoform of A0A3R7N5V9, AAA+ ATPase domain-containing protein | A0A3R7MCD0\|A0A3R7MCD0_PENVA | 31236.408 | 50.056477 | 1 | 1 |  |
| 972 | Uncharacterized protein | A0A3R7PKW3\|A0A3R7PKW3_PENVA | 88009.45 | 49.610546 | 1 | 1 |  |
| 973 | Peroxin-19 | A0A3R7MBG2\|A0A3R7MBG2_PENVA | 37966.215 | 49.073742 | 1 | 1 |  |
| 974 | Putative mucin-5AC | A0A423TI79\|A0A423TI79_PENVA | 58952.6 | 48.705765 | 1 | 1 |  |
| 975 | Uncharacterized protein | A0A423TJ76\|A0A423TJ76_PENVA | 58731.12 | 48.486298 | 2 | 2 | Acetylation (N-term) |
| 976 | Putative striatin-interacting protein 2-like | A0A423TAC9\|A0A423TAC9_PENVA | 14746.654 | 34.204266 | 1 | 1 | Acetylation (N-term) |
| 977 | Uncharacterized protein | A0A3R7PV77\|A0A3R7PV77_PENVA | 64908.977 | 34.204266 | 1 | 1 | Acetylation (N-term) |
| 978 | Uncharacterized protein (Fragment) | A0A3R7PF44\|A0A3R7PF44_PENVA | 94205.68 | 34.204266 | 1 | 1 | Acetylation (N-term) |
| 979 | Organic cation transporter protein | A0A3R7PRN4\|A0A3R7PRN4_PENVA | 103202.56 | 34.204266 | 1 | 1 | Acetylation (N-term) |
| 980 | Uncharacterized protein | A0A423SVY3\|A0A423SVY3_PENVA | 41993.613 | 34.204266 | 1 | 1 | Acetylation (N-term) |
| 981 | Uncharacterized protein | A0A3R7MKG8\|A0A3R7MKG8_PENVA | 51471.598 | 34.204266 | 1 | 1 | Acetylation (N-term) |
| 982 | Midasin | A0A3R7NVK2\|A0A3R7NVK2_PENVA | 484496.72 | 34.204266 | 1 | 1 | Acetylation (N-term) |
| 983 | Uncharacterized protein | A0A423THD7\|A0A423THD7_PENVA | 58432.793 | 34.204266 | 1 | 1 | Acetylation (N-term) |
| 984 | Uncharacterized protein | A0A3R7QM36\|A0A3R7QM36_PENVA | 51653.605 | 34.204266 | 1 | 1 | Acetylation (N-term) |
| 985 | Janus kinase | A0A3R7QGT2\|A0A3R7QGT2_PENVA | 26093.3 | 34.204266 | 1 | 1 | Acetylation (N-term) |
| 986 | Organic cation transporter protein | A0A423TEK6\|A0A423TEK6_PENVA | 59499.203 | 34.204266 | 1 | 1 | Acetylation (N-term) |
| 987 | Uncharacterized protein | A0A3R7MJB2\|A0A3R7MJB2_PENVA | 151921.64 | 34.204266 | 1 | 1 | Acetylation (N-term) |
| 988 | Uncharacterized protein | A0A3R7QLC5\|A0A3R7QLC5_PENVA | 41618.688 | 34.204266 | 1 | 1 | Acetylation (N-term) |
| 989 | Putative organic cation transporter protein-like | A0A423TEI7\|A0A423TEI7_PENVA | 65328.895 | 34.204266 | 1 | 1 | Acetylation (N-term) |
| 990 | Putative organic cation transporter protein-like | A0A3R7N1A2\|A0A3R7N1A2_PENVA | 46479.69 | 34.204266 | 1 | 1 | Acetylation (N-term) |
| 991 | ER lumen protein-retaining receptor | A0A3R7MD70\|A0A3R7MD70_PENVA | 24517.861 | 34.204266 | 1 | 1 | Acetylation (N-term) |
| 992 | Putative cell agglutination protein PB2C8.01 | A0A423TDH3\|A0A423TDH3_PENVA | 91949.45 | 34.204266 | 1 | 1 | Acetylation (N-term) |
| 993 | Gustatory receptor | A0A3R7QKY1\|A0A3R7QKY1_PENVA | 100432.164 | 34.204266 | 1 | 1 | Acetylation (N-term) |
| 994 | Putative UPF0462 protein C4orf33-like (Fragment) | A0A3R7PXW2\|A0A3R7PXW2_PENVA | 95593.92 | 34.204266 | 1 | 1 | Acetylation (N-term) |
| 995 | Putative organic cation transporter protein-like | A0A3R7M798\|A0A3R7M798_PENVA | 50963.855 | 34.204266 | 1 | 1 | Acetylation (N-term) |
| 996 | Uncharacterized protein | A0A3R7M4R6\|A0A3R7M4R6_PENVA | 58328.785 | 28.564062 | 1 | 1 |  |
| 997 | Protein MMS22-like | A0A423TB31\|A0A423TB31_PENVA | 136309.56 | 28.564062 | 1 | 1 |  |
| 998 | Gustatory receptor | A0A3R7LRQ1\|A0A3R7LRQ1_PENVA | 48135.953 | 28.564062 | 1 | 1 |  |
| 999 | Putative mucin-2-like | A0A423TJ68\|A0A423TJ68_PENVA | 97544.75 | 28.564062 | 1 | 1 |  |
| 1000 | Uncharacterized protein | A0A3R7M9U5\|A0A3R7M9U5_PENVA | 64571.54 | 28.564062 | 1 | 1 |  |
| 1001 | Gustatory receptor | A0A423U4N6\|A0A423U4N6_PENVA | 51340.15 | 28.564062 | 1 | 1 |  |
| 1002 | Uncharacterized protein | A0A423TZX4\|A0A423TZX4_PENVA | 77875.14 | 28.564062 | 1 | 1 |  |
| 1003 | 40S ribosomal protein S15 | A0A3R7PII3\|A0A3R7PII3_PENVA | 17556.764 | 48.462593 | 1 | 1 |  |
| 1004 | Protein unc-45 homolog B | A0A3R7MQG8\|A0A3R7MQG8_PENVA | 104474.95 | 47.605278 | 1 | 1 |  |
| 1005 | Uncharacterized protein | A0A423TY65\|A0A423TY65_PENVA | 40416.445 | 47.26094 | 1 | 1 |  |
| 1006 | Electron transfer flavoprotein subunit alpha | A0A3R7QMR5\|A0A3R7QMR5_PENVA | 34628.953 | 46.3934 | 1 | 1 | Deamidation (NQ) |
| 1007 | Cuticle protein CB5 | A0A3R7QK62\|A0A3R7QK62_PENVA | 14763.355 | 46.353573 | 1 | 1 |  |
| 1008 | Cuticle protein CB5 | A0A423TSV6\|A0A423TSV6_PENVA | 10867.836 | 46.353573 | 1 | 1 |  |
| 1009 | Vinculin | A0A423U369\|A0A423U369_PENVA | 11802.772 | 45.958073 | 1 | 1 |  |
| 1010 | RNA-binding protein 8A | A0A423SXF6\|A0A423SXF6_PENVA | 18597.617 | 45.672054 | 1 | 1 | Acetylation (N-term) |
| 1011 | Rab GDP dissociation inhibitor | A0A3R7N6N6\|A0A3R7N6N6_PENVA | 47880.56 | 45.233067 | 1 | 1 |  |
| 1012 | Heterotrimeric GTP-binding protein alpha subunit G-alpha-q | Q6ITD0\|Q6ITD0_PENVA | 41392.254 | 43.29308 | 1 | 1 |  |
| 1013 | NADH dehydrogenase [ubiquinone] flavoprotein 1, mitochondrial | A0A3R7NRV9\|A0A3R7NRV9_PENVA | 50653.11 | 42.36789 | 1 | 1 | Carbamidomethylation |
| 1014 | Flightless-I | S4VUL7\|S4VUL7_PENVA | 148360.9 | 42.168095 | 1 | 1 |  |
| 1015 | Uncharacterized protein | A0A423U987\|A0A423U987_PENVA | 111942.914 | 41.14874 | 1 | 1 |  |
| 1016 | Putative MDS1 and EVI1 complex locus protein EVI1-A | A0A3R7N020\|A0A3R7N020_PENVA | 54148.29 | 40.866695 | 1 | 1 | Carbamidomethylation |
| 1017 | Ferritin 2 | A0A423U462\|A0A423U462_PENVA | 9547.855 | 40.54457 | 1 | 1 |  |
| 1018 | Small ribosomal subunit protein uS4 | A0A3R7P9M4\|A0A3R7P9M4_PENVA | 22628.488 | 40.315334 | 1 | 1 |  |
| 1019 | Crustacean hyperglycemic hormone 2 | A0A3R7PVG1\|A0A3R7PVG1_PENVA | 16355.656 | 40.178726 | 1 | 1 |  |
| 1020 | Putative FERM, RhoGEF and pleckstrin domain-containing protein 2 | A0A3R7PG31\|A0A3R7PG31_PENVA | 120408.836 | 39.94163 | 1 | 1 |  |
| 1021 | Isoform of A0A3R7LVG0, Uncharacterized protein | A0A423SMZ0\|A0A423SMZ0_PENVA | 37363.645 | 38.238518 | 1 | 1 |  |
| 1022 | Putative hemocytin | A0A3R7P7D5\|A0A3R7P7D5_PENVA | 362894.62 | 37.89025 | 1 | 1 | Carbamidomethylation |
| 1023 | Phosphoribosyl pyrophosphate synthetase-associated protein 2 | A0A423SDW4\|A0A423SDW4_PENVA | 21768.482 | 36.59076 | 1 | 1 |  |
| 1024 | Mediator of RNA polymerase II transcription subunit 13 | A0A3R7PG97\|A0A3R7PG97_PENVA | 188665.27 | 36.142464 | 2 | 1 |  |
| 1025 | Putative 60S ribosomal protein L18a-like | A0A423T897\|A0A423T897_PENVA | 25065.244 | 35.2484 | 1 | 1 |  |
| 1026 | Glutathione synthetase | A0A423TBF6\|A0A423TBF6_PENVA | 26246.441 | 34.546455 | 1 | 1 | Carbamidomethylation |
| 1027 | Uncharacterized protein | A0A423TT13\|A0A423TT13_PENVA | 64224.62 | 34.229702 | 1 | 1 |  |
| 1028 | N-chimaerin | A0A423TFE3\|A0A423TFE3_PENVA | 53530.867 | 34.229702 | 1 | 1 |  |
| 1029 | Putative SLIT-ROBO Rho GTPase-activating protein 1-like isoform X3 | A0A423SQ28\|A0A423SQ28_PENVA | 153763.83 | 34.229702 | 1 | 1 |  |
| 1030 | P3 protein | A0A3R7LYD7\|A0A3R7LYD7_PENVA | 53223.992 | 34.229702 | 1 | 1 |  |
| 1031 | Putative ryanodine receptor 44F isoform X1 | A0A3R7T1F9\|A0A3R7T1F9_PENVA | 40891.277 | 33.147263 | 1 | 1 |  |
| 1032 | Putative nucleolar pre-ribosomal-associated protein 1-like | A0A3R7PA56\|A0A3R7PA56_PENVA | 219522.72 | 33.04338 | 2 | 1 |  |
| 1033 | RRM domain-containing protein | A0A3R7PAJ9\|A0A3R7PAJ9_PENVA | 56068.195 | 32.39508 | 1 | 1 |  |
| 1034 | Putative coiled-coil domain-containing protein 77-like | A0A3R7MKU2\|A0A3R7MKU2_PENVA | 55814.03 | 30.669743 | 1 | 1 | Deamidation (NQ); Oxidation (M) |
| 1035 | PRA1 family protein | A0A3R7QF06\|A0A3R7QF06_PENVA | 24065.516 | 29.60027 | 1 | 1 |  |
| 1036 | Putative organic cation transporter protein | A0A423SMJ6\|A0A423SMJ6_PENVA | 47525.594 | 29.529945 | 1 | 1 |  |
| 1037 | Vinculin | A0A423U364\|A0A423U364_PENVA | 25624.941 | 29.469055 | 1 | 1 |  |
| 1038 | glutathione transferase | A0A423TR82\|A0A423TR82_PENVA | 77063.59 | 29.169048 | 1 | 1 | Carbamidomethylation |
| 1039 | Transgelin | A0A423U9T8\|A0A423U9T8_PENVA | 20382.031 | 28.948908 | 1 | 1 |  |
| 1040 | Crustacean calcium-binding protein 23 | A0A3R7QTG8\|A0A3R7QTG8_PENVA | 45915.816 | 28.716358 | 1 | 1 |  |
| 1041 | Putative trifunctional enzyme subunit beta, mitochondrial | A0A423U7W2\|A0A423U7W2_PENVA | 50631.41 | 28.712032 | 1 | 1 |  |
| 1042 | Uncharacterized protein | A0A423SW35\|A0A423SW35_PENVA | 86466.74 | 28.517792 | 1 | 1 |  |
| 1043 | Uncharacterized protein | A0A3R7NP54\|A0A3R7NP54_PENVA | 311813.94 | 28.517792 | 1 | 1 |  |
| 1044 | Uncharacterized protein | A0A423S9Q5\|A0A423S9Q5_PENVA | 41911.676 | 28.517792 | 1 | 1 |  |
| 1045 | Uncharacterized protein | A0A423TWA3\|A0A423TWA3_PENVA | 39845.055 | 28.517792 | 1 | 1 |  |
| 1046 | Protein kinase domain-containing protein | A0A3R7MG08\|A0A3R7MG08_PENVA | 161966.8 | 28.517792 | 1 | 1 |  |
| 1047 | Putative eukaryotic translation initiation factor 4 gamma 2 isoform X1 | A0A3R7NMU4\|A0A3R7NMU4_PENVA | 99931.77 | 28.517792 | 1 | 1 |  |
| 1048 | Putative ubiquitin carboxyl-terminal hydrolase 36 | A0A423TZC4\|A0A423TZC4_PENVA | 141280.4 | 28.517792 | 1 | 1 |  |
| 1049 | Uncharacterized protein | A0A3R7PM26\|A0A3R7PM26_PENVA | 69548.96 | 28.517792 | 1 | 1 |  |
| 1050 | Uncharacterized protein (Fragment) | A0A3R7NB88\|A0A3R7NB88_PENVA | 35923.23 | 28.517792 | 1 | 1 |  |
| 1051 | Calcium release-activated calcium channel protein 1 | A0A423TLB8\|A0A423TLB8_PENVA | 39064.94 | 28.517792 | 1 | 1 |  |
| 1052 | Sodium-dependent phosphate transporter | A0A423TZ68\|A0A423TZ68_PENVA | 62554.78 | 28.156181 | 1 | 1 |  |
| 1053 | Two pore calcium channel protein 1 | A0A3R7QF20\|A0A3R7QF20_PENVA | 89501.64 | 28.156181 | 1 | 1 |  |
| 1054 | Putative histone-lysine N-methyltransferase ash1-like | A0A3R7MN94\|A0A3R7MN94_PENVA | 192360.94 | 28.038177 | 1 | 1 |  |
| 1055 | Transient receptor potential channel pyrexia | A0A423SW15\|A0A423SW15_PENVA | 136611.27 | 28.038177 | 1 | 1 |  |
| 1056 | Vacuolar protein sorting-associated protein 33A | A0A3R7SJD0\|A0A3R7SJD0_PENVA | 64755.188 | 27.962784 | 1 | 1 | Deamidation (NQ) |
| 1057 | Matrix metalloproteinase | A0A423SVD6\|A0A423SVD6_PENVA | 195034.81 | 27.830154 | 1 | 1 |  |
| 1058 | ZAD domain-containing protein | A0A423T6J9\|A0A423T6J9_PENVA | 109680.945 | 27.600967 | 1 | 1 | Acetylation (N-term) |
| 1059 | Surfeit locus protein 4-like | A0A423TAR3\|A0A423TAR3_PENVA | 31026.01 | 27.029255 | 1 | 1 |  |
