# Supplementary material for: Shrimp allergen extract immunotherapy induces prolonged immune tolerance in a gastro-food allergy mouse model
Source: PLoS One. 2024 Dec 27;19(12):e0315312. doi: 10.1371/journal.pone.0315312 (PMC11676511; doi:10.1371/journal.pone.0315312)
Supplement: S2 Dataset — A. Systemic Allergy Scores. B. Evaluation of FcεR1α mRNA Relative Expression. C. Degranulated Mast Cells Analysis. D. Evaluation of IL-4 mRNA Relative Expression. E. IL-4 Serum Level Analysis. F. Evaluation of Foxp3 mRNA Relative Expression. G. Evaluation of IL-10 mRNA Relative Expression. H. Evaluation of IL-10/IL-4 mRNA Ratio. (DOCX) [file pone.0315312.s004.docx]

***S2 Dataset of Experimental Study***

**Shrimp allergen extract immunotherapy induces prolonged immune tolerance in a gastro-food allergy mouse model**

Honey Dzikri Marhaeny^1^, Lutfiatur Rohmah^1^, Yusuf Alif Pratama^1^, Salsabilla Madudari Kasatu^1^, Andang Miatmoko^2^, Rafi Addimaysqi^3^, Geert van den Bogaart^4^, Franz Y. Ho^5^, Muhammad Taher^6^, Junaidi Khotib^1*^

^1^Department of Pharmacy Practice, Faculty of Pharmacy, Airlangga University, Surabaya, Indonesia

^2^Department of Pharmaceutical Science, Faculty of Pharmacy, Airlangga University, Surabaya, Indonesia

^3^Faculty of Medicine, Airlangga University, Surabaya, Indonesia

^4^Department of Molecular Immunology and Microbiology, Groningen Biomolecular Sciences and Biotechnology Institute, Faculty of Science Engineering, University of Groningen, Groningen, The Netherlands

^5^GBB Proteomics, Groningen Biomolecular Sciences and Biotechnology Institute, Faculty of Science Engineering, University of Groningen, Groningen, The Netherlands

^6^Department of Pharmaceutical Technology, Kulliyyah of Pharmacy, International Islamic University Malaysia, Kuantan, Pahang, Malaysia

***Corresponding Author:**

Email: [junaidi-k@ff.unair.ac.id](mailto:junaidi-k@ff.unair.ac.id) (JK)

**S2A Dataset.** Systemic Allergy Scores

| **n** | **1^st^ Challenge** | | | **2^nd^ Challenge** | **3^rd^ Challenge** |  |
| --- | --- | --- | --- | --- | --- | --- |
|  | **1** | **2** | **3** |  |  |  |
| **Untreated Group** | | | | | | |
| Un 1 | 0 | 0 | 0 | 1 | 0 |  |
| Un 2 | 0 | 0 | 0 | 0 | 0 |  |
| Un 3 | 0 | 1 | 1 | 0 | 1 |  |
| Un 4 | 0 | 0 | 0 | 0 | 0 |  |
| Un 5 | 0 | 0 | 0 | 0 | 0 |  |
| Un 6 | 1 | 0 | 0 | 0 | 0 |  |
| **SEM** | **0.167** | **0.167** | **0.167** | **0.167** | **0.167** |  |
| **Negative Control Group** | | | | | | |
| NC 1 | 1 | 2 | 2 | 2 | 2 |  |
| NC 2 | 1 | 1 | 2 | 2 | 2 |  |
| NC 3 | 1 | 2 | 2 | 2 | 2 |  |
| NC 4 | 2 | 2 | 2 | 2 | 2 |  |
| NC 5 | 1 | 2 | 2 | 2 | 2 |  |
| NC 6 | 2 | 2 | 2 | 2 | 2 |  |
| **SEM** | **0.211** | **0.167** | **0.000** | **0.000** | **0.000** |  |
| **High-dose Immunotherapy Group** | | | | | | |
| HI 1 | 1 | 2 | 2 | 1 | 0 |  |
| HI 2 | 2 | 2 | 2 | 0 | 0 |  |
| HI 3 | 1 | 2 | 2 | 0 | 0 |  |
| HI 4 | 1 | 1 | 2 | 1 | 0 |  |
| HI 5 | 1 | 2 | 2 | 0 | 1 |  |
| HI 6 | 1 | 2 | 2 | 0 | 0 |  |
| **SEM** | **0.167** | **0.167** | **0.000** | **0.211** | **0.167** |  |
| **Moderate-dose Immunotherapy Group** | | | | | | |
| MI 1 | 1 | 2 | 2 | 0 | 1 |  |
| MI 2 | 1 | 2 | 2 | 1 | 0 |  |
| MI 3 | 2 | 2 | 2 | 1 | 0 |  |
| MI 4 | 1 | 2 | 2 | 1 | 0 |  |
| MI 5 | 1 | 2 | 2 | 0 | 0 |  |
| MI 6 | 1 | 2 | 2 | 0 | 1 |  |
| **SEM** | **0.167** | **0.000** | **0.000** | **0.224** | **0.211** |  |
| **Low-dose Immunotherapy Group** | | | | | | |
| LI 1 | 1 | 2 | 2 | 1 | 0 |  |
| LI 2 | 2 | 2 | 2 | 1 | 1 |  |
| LI 3 | 1 | 2 | 2 | 0 | 1 |  |
| LI 4 | 2 | 2 | 2 | 0 | 1 |  |
| LI 5 | 2 | 2 | 2 | 1 | 0 |  |
| LI 6 | 1 | 2 | 2 | 1 | 1 |  |
| **SEM** | **0.224** | **0.000** | **0.000** | **0.211** | **0.211** |  |

Systemic allergy symptom assessment data (ordinal data) were analyzed using the Kruskal-Wallis Test.

**S2B Dataset.** Evaluation of FcεR1α mRNA Relative Expression

| **n** | **Fold Change** | **Mean** | **SEM** |
| --- | --- | --- | --- |
| **Untreated Group** | | | |
| Un 1 | 0.085 | 0.085 | 0.019 |
| Un 2 | 0.136 |  |  |
| Un 3 | 0.141 |  |  |
| Un 4 | 0.027 |  |  |
| Un 5 | 0.063 |  |  |
| Un 6 | 0.058 |  |  |
| **Negative Control Group** | | | |
| NC 1 | 0.409 | 1.000^###^ | 0.280 |
| NC 2 | 0.351 |  |  |
| NC 3 | 1.063 |  |  |
| NC 4 | 1.514 |  |  |
| NC 5 | 2.069 |  |  |
| NC 6 | 0.594 |  |  |
| **High-dose Immunotherapy Group** | | | |
| HI 1 | 0.128 | 0.103^***^ | 0.036 |
| HI 2 | 0.099 |  |  |
| HI 3 | 0.034 |  |  |
| HI 4 | 0.011 |  |  |
| HI 5 | 0.262 |  |  |
| HI 6 | 0.084 |  |  |
| **Moderate-dose Immunotherapy Group** | | | |
| MI 1 | 0.607 | 0.221^***^ | 0.085 |
| MI 2 | 0.220 |  |  |
| MI 3 | 0.150 |  |  |
| MI 4 | 0.066 |  |  |
| MI 5 | 0.028 |  |  |
| MI 6 | 0.255 |  |  |
| **Low-dose Immunotherapy Group** | | | |
| LI 1 | 0.057 | 0.300^**^ | 0.131 |
| LI 2 | 0.846 |  |  |
| LI 3 | 0.068 |  |  |
| LI 4 | 0.351 |  |  |
| LI 5 | 0.027 |  |  |
| LI 6 | 0.453 |  |  |

p-values of *FcεR1α* expression were derived from one-way ANOVA test (^###^, p≤0.001 were significant against Untreated and ^***^, p≤0.001; ^**^, p≤0.01 were significant against NC).

**S2C Dataset.** Degranulated Mast Cells Analysis

| **n** | **% Degranulated Mast Cells** | **Mean** | **SEM** |
| --- | --- | --- | --- |
| **Untreated Group** | | | |
| Un 1 | 35.20% | 35.45%^#^ | 0.008 |
| Un 2 | 35.51% |  |  |
| Un 3 | 33.87% |  |  |
| Un 4 | 33.77% |  |  |
| Un 5 | 39.13% |  |  |
| Un 6 | 35.20% |  |  |
| **Negative Control Group** | | | |
| NC 1 | 43.38% | 44.43%^####^ | 0.007 |
| NC 2 | 46.22% |  |  |
| NC 3 | 42.97% |  |  |
| NC 4 | 44.30% |  |  |
| NC 5 | 43.06% |  |  |
| NC 6 | 46.67% |  |  |
| **High-dose Immunotherapy Group** | | | |
| HI 1 | 32.39% | 32.57%^****^ | 0.014 |
| HI 2 | 29.38% |  |  |
| HI 3 | 32.38% |  |  |
| HI 4 | 35.20% |  |  |
| HI 5 | 37.38% |  |  |
| HI 6 | 28.70% |  |  |
| **Moderate-dose Immunotherapy Group** | | | |
| MI 1 | 39.29% | 38.43%^***^ | 0.006 |
| MI 2 | 35.77% |  |  |
| MI 3 | 37.50% |  |  |
| MI 4 | 38.79% |  |  |
| MI 5 | 39.44% |  |  |
| MI 6 | 39.78% |  |  |
| **Low-dose Immunotherapy Group** | | | |
| LI 1 | 35.04% | 39.49%^**^ | 0.013 |
| LI 2 | 40.00% |  |  |
| LI 3 | 43.23% |  |  |
| LI 4 | 37.61% |  |  |
| LI 5 | 38.57% |  |  |
| LI 6 | 42.47% |  |  |

p-values were derived from one-way ANOVA test (^####^, p≤0.0001 were significant against Untreated and ^****^, p≤0.0001; ^***^, p≤0.001; ^**^, p≤0.01; were significant against NC).

**S2D Dataset.** Evaluation of IL-4 mRNA Relative Expression

| **n** | **Fold Change** | **Mean** | **SEM** | |  |
| --- | --- | --- | --- | --- | --- |
| **Untreated Group** | | | | | |
| Un 1 | 0.111 | 0.128 | 0.011 | |  |
| Un 2 | 0.150 |  |  |  |  |
| Un 3 | 0.124 |  |  |  |  |
| **Negative Control Group** | | | | | |
| NC 1 | 0.930 | 1.000^##^ | 0.241 | |  |
| NC 2 | 1.449 |  |  |  |  |
| NC 3 | 0.622 |  |  |  |  |
| **High-dose Immunotherapy Group** | | | | | |
| HI 1 | 0.315 | 0.325^**^ | 0.031 | |  |
| HI 2 | 0.278 |  |  |  |  |
| HI 3 | 0.383 |  |  |  |  |
| **Moderate-dose Immunotherapy Group** | | | | | |
| MI 1 | 0.495 | 0.427^*^ | 0.099 |  |  |
| MI 2 | 0.553 |  |  |  |  |
| MI 3 | 0.232 |  |  |  |  |
| **Low-dose Immunotherapy Group** | | | | | |
| LI 1 | 0.523 | 0.588 | 0.150 | |  |
| LI 2 | 0.367 |  |  |  |  |
| LI 3 | 0.873 |  |  |  |  |

p-values of IL-4 mRNA expression were derived from one-way ANOVA test (^##^, p≤0.01 were significant against Untreated and ^**^, p≤0.01; ^*^, p≤0.05 were significant against NC).

**S2E Dataset.** IL-4 Serum Level Analysis

| **n** | **IL-4 Serum Level (pg/mL)** | **Mean** | **SEM** |  |
| --- | --- | --- | --- | --- |
| **Untreated Group** | | | | |
| Un 1 | 9.567 | 10.345^*^ | 0.806 |  |
| Un 2 | 9.233 |  |  |  |
| Un 3 | 9.900 |  |  |  |
| Un 4 | 7.900 |  |  |  |
| Un 5 | 12.567 |  |  |  |
| Un 6 | 12.900 |  |  |  |
| **Negative Control Group** | | | | |
| NC 1 | 62.125 | 63.167^#^ | 0.995 |  |
| NC 2 | 63.625 |  |  |  |
| NC 3 | 67.625 |  |  |  |
| NC 4 | 60.375 |  |  |  |
| NC 5 | 62.375 |  |  |  |
| NC 6 | 62.875 |  |  |  |
| **High-dose Immunotherapy Group** | | | | |
| HI 1 | 0.000 | 1.942^****^ | 1.942 |  |
| HI 2 | 0.000 |  |  |  |
| HI 3 | 0.000 |  |  |  |
| HI 4 | 11.652 |  |  |  |
| HI 5 | 0.000 |  |  |  |
| HI 6 | 0.000 |  |  |  |
| **Moderate-dose Immunotherapy Group** | | | | |
| MI 1 | 10.130 | 9.250^*^ | 2.152 |  |
| MI 2 | 10.348 |  |  |  |
| MI 3 | 10.565 |  |  |  |
| MI 4 | 8.233 |  |  |  |
| MI 5 | 16.226 |  |  |  |
| MI 6 | 0.000 |  |  |  |
| **Low-dose Immunotherapy Group** | | | | |
| LI 1 | 10.130 | 10.336^*^ | 1.061 |  |
| LI 2 | 12.304 |  |  |  |
| LI 3 | 8.391 |  |  |  |
| LI 4 | 14.567 |  |  |  |
| LI 5 | 8.391 |  |  |  |
| LI 6 | 8.233 |  |  |  |

p-values of IL-4 serum levels were derived from Kruskal-Wallis test (#, p≤0.05 were significant against Untreated and ****, p≤0.0001; *, p≤0.05 were significant against NC).

**S2F Dataset.** Evaluation of Foxp3 mRNA Relative Expression

| **n** | **Fold Change** | **Mean** | **SEM** |
| --- | --- | --- | --- |
| **Untreated Group** | | | |
| Un 1 | 1.773 | 1.396 | 0.199 |
| Un 2 | 1.307 |  |  |
| Un 3 | 0.887 |  |  |
| Un 4 | 1.954 |  |  |
| Un 5 | 1.678 |  |  |
| Un 6 | 0.777 |  |  |
| **Negative Control Group** | | | |
| NC 1 | 1.471 | 1.000 | 0.227 |
| NC 2 | 1.565 |  |  |
| NC 3 | 0.082 |  |  |
| NC 4 | 1.054 |  |  |
| NC 5 | 1.178 |  |  |
| NC 6 | 0.649 |  |  |
| **High-dose Immunotherapy Group** | | | |
| HI 1 | 4.277 | 4.405^****^ | 0.489 |
| HI 2 | 4.018 |  |  |
| HI 3 | 4.745 |  |  |
| HI 4 | 6.218 |  |  |
| HI 5 | 4.648 |  |  |
| HI 6 | 2.525 |  |  |
| **Moderate-dose Immunotherapy Group** | | | |
| MI 1 | 4.102 | 2.312^*^ | 0.457 |
| MI 2 | 3.219 |  |  |
| MI 3 | 1.544 |  |  |
| MI 4 | 1.901 |  |  |
| MI 5 | 1.154 |  |  |
| MI 6 | 1.954 |  |  |
| **Low-dose Immunotherapy Group** | | | |
| LI 1 | 2.508 | 2.086^*^ | 0.298 |
| LI 2 | 3.264 |  |  |
| LI 3 | 1.862 |  |  |
| LI 4 | 1.151 |  |  |
| LI 5 | 1.666 |  |  |
| LI 6 | 2.066 |  |  |

p-values were derived from one-way ANOVA (****, p≤0.0001; *, p≤0.05 were significant against NC).

**S2G Dataset.** Evaluation of IL-10 mRNA Relative Expression

| **n** | **Fold Change** | **Mean** | **SEM** | |
| --- | --- | --- | --- | --- |
| **Untreated Group** | | | | |
| Un 1 | 0.080 | 0.123 | 0.026 | |
| Un 2 | 0.169 |  |  |  |
| Un 3 | 0.121 |  |  |  |
| **Negative Control Group** | | | | |
| NC 1 | 1.282 | 1.000^##^ | 0.192 | |
| NC 2 | 0.632 |  |  |  |
| NC 3 | 1.086 |  |  |  |
| **High-dose Immunotherapy Group** | | | | |
| HI 1 | 2.477 | 2.433^***^ | 0.174 | |
| HI 2 | 2.710 |  |  |  |
| HI 3 | 2.112 |  |  |  |
| **Moderate-dose Immunotherapy Group** | | | | |
| MI 1 | 1.657 | 1.664^*^ | | 0.223 |
| MI 2 | 2.054 |  |  |  |
| MI 3 | 1.282 |  |  |  |
| **Low-dose Immunotherapy Group** | | | | |
| LI 1 | 1.788 | 1.347 | 0.231 | |
| LI 2 | 1.006 |  |  |  |
| LI 3 | 1.247 |  |  |  |

p-values were derived from one-way ANOVA (^##^, p≤0.01; were significant against Untreated and ^***^, p≤0.001; ^*^, p≤0.05 were significant against NC).

**S2H Dataset.** Evaluation of IL-10/IL-4 mRNA Ratio

| **n** | **Fold Change Ratio** | **Mean** | **SEM** | |
| --- | --- | --- | --- | --- |
| **Untreated Group** | | | | |
| Un 1 | 0.714 | 0.941 | 0.121 | |
| Un 2 | 1.128 |  |  |  |
| Un 3 | 0.982 |  |  |  |
| **Negative Control Group** | | | | |
| NC 1 | 1.379 | 1.187 | 0.390 | |
| NC 2 | 0.436 |  |  |  |
| NC 3 | 1.746 |  |  |  |
| **High-dose Immunotherapy Group** | | | | |
| HI 1 | 7.857 | 7.509^****^ | 0.228 | |
| HI 2 | 7.081 |  |  |  |
| HI 3 | 7.589 |  |  |  |
| **Moderate-dose Immunotherapy Group** | | | | |
| MI 1 | 3.349 | 4.194^***^ | | 0.670 |
| MI 2 | 3.716 |  |  |  |
| MI 3 | 5.517 |  |  |  |
| **Low-dose Immunotherapy Group** | | | | |
| LI 1 | 2.048 | 2.391^*^ | 0.200 | |
| LI 2 | 2.739 |  |  |  |
| LI 3 | 2.385 |  |  |  |

p-values were derived from one-way ANOVA (^****^, p≤0.0001; ^***^, p≤0.001; ^*^, p≤0.05 were significant against NC).
